# Assessment of Inpatient Time Allocation Among First-Year Internal Medicine Residents Using Time-Motion Observations

**DOI:** 10.1001/jamainternmed.2019.0095

**Published:** 2019-06-01

**Authors:** Krisda H. Chaiyachati, Judy A. Shea, David A. Asch, Manqing Liu, Lisa M. Bellini, C. Jessica Dine, Alice L. Sternberg, Yevgeniy Gitelman, Alyssa M. Yeager, Jeremy M. Asch, Sanjay V. Desai

**Affiliations:** Department of Medicine, Perelman School of Medicine at the University of Pennsylvania, Philadelphia; Leonard Davis Institute of Health Economics, University of Pennsylvania, Philadelphia; Department of Medicine, Perelman School of Medicine at the University of Pennsylvania, Philadelphia; Leonard Davis Institute of Health Economics, University of Pennsylvania, Philadelphia; Department of Medicine, Perelman School of Medicine at the University of Pennsylvania, Philadelphia; Leonard Davis Institute of Health Economics, University of Pennsylvania, Philadelphia; Corporal Michael J. Crescenz Veterans Affairs Medical Center, Philadelphia, Pennsylvania; Department of Medicine, Perelman School of Medicine at the University of Pennsylvania, Philadelphia; Leonard Davis Institute of Health Economics, University of Pennsylvania, Philadelphia; Department of Medicine, Perelman School of Medicine at the University of Pennsylvania, Philadelphia; Department of Medicine, Perelman School of Medicine at the University of Pennsylvania, Philadelphia; Leonard Davis Institute of Health Economics, University of Pennsylvania, Philadelphia; Department of Epidemiology, Johns Hopkins University, Baltimore, Maryland; Department of Medicine, Perelman School of Medicine at the University of Pennsylvania, Philadelphia; Department of Medicine, Yale-New Haven Hospital, New Haven, Connecticut; Department of Emergency Medicine, University of Pennsylvania, Philadelphia; Department of Medicine, Johns Hopkins University, Baltimore, Maryland

## Abstract

**IMPORTANCE:**

The United States spends more than $12 billion annually on graduate medical education. Understanding how residents balance patient care and educational activities may provide insights into how the modern physician workforce is being trained.

**OBJECTIVE:**

To describe how first-year internal medicine residents (interns) allocate time while working on general medicine inpatient services.

**DESIGN, SETTING, AND PARTICIPANTS:**

Direct observational secondary analysis, including 6 US university-affiliated and community-based internal medicine programs in the mid-Atlantic region, of the Comparative Effectiveness of Models Optimizing Patient Safety and Resident Education (iCOMPARE) trial, a cluster-randomized trial comparing different duty-hour policies. A total of 194 weekday shifts were observed and time motion data were collected, sampled by daytime, nighttime, and call shifts in proportion to the distribution of shifts within each program from March 10 through May 31, 2016. Data were analyzed from June 1, 2016, through January 5, 2019.

**MAIN OUTCOMES AND MEASURES:**

Mean time spent in direct and indirect patient care, education, rounds, handoffs, and miscellaneous activities within a 24-hour period and in each of four 6-hour periods (morning, afternoon, evening, and night). Time spent multitasking, simultaneously engaged in combinations of direct patient care, indirect patient care, or education, and in subcategories of indirect patient care were tracked.

**RESULTS:**

A total of 80 interns (55% men; mean [SD] age, 28.7 [2.3] years) were observed across 194 shifts, totaling 2173 hours. A mean (SD) of 15.9 (0.7) hours of a 24-hour period (66%) was spent in indirect patient care, mostly interactions with the patient’s medical record or documentation (mean [SD], 10.3 [0.7] hours; 43%). A mean (SD)of 3.0 (0.1) hours was spent in direct patient care (13%) and 1.8 (0.3) hours in education (7%). This pattern was consistent across the 4 periods of the day. Direct patient care and education frequently occurred when interns were performing indirect patient care. Multitasking with 2 or more indirect patient care activities occurred for a mean (SD) of 3.8 (0.4) hours (16%) of the day.

**CONCLUSIONS AND RELEVANCE:**

This study’s findings suggest that within these US teaching programs, interns spend more time participating in indirect patient care than interacting with patients or in dedicated educational activities. These findings provide an essential baseline measure for future efforts designed to improve the workday structure and experience of internal medicine trainees, without making a judgment on the current allocation of time.

**TRIAL REGISTRATION:**

ClinicalTrials.gov identifier: NCT02274818

The workday for internal medicine residents in the United States has evolved over time. With the diffusion of the electronic health record, demands for more detailed documentation, and pressures to decrease the length of stay for common clinical conditions,^1,2^ residents may have adapted by reducing time spent with patients and in educational activities. Indeed, prior studies until the late 2000s observed that first-year residents (interns) spent only 9% to 12% of inpatient time with patients,^3-5^ less than half the time observed in the 1990s.^6^ These studies, however, have limited generalizability because they represent data from few trainees, limited the types of shifts, and focused on daytime activities.^7-9^

Understanding how residents spend their time is important because of the likely effect of these activities on the quality and function of the physician workforce and because the United States spends $12 to $14 billion annually on residency training.^10,11^ We present data from direct time-motion observations of interns at 6 internal medicine training programs randomized within in the individualized Comparative Effectiveness of Models Optimizing Patient Safety and Resident Education (iCOMPARE) trial, a cluster-randomized trial comparing 2 different duty-hour policies during the 2015-2016 academic year.^12^ A previous study^13^ reported no differences between duty-hour policy groups in time spent in direct patient care or education as 1 of 4 prespecified hypotheses from the trial pertaining to trainee education; herein we report more detailed descriptions of time allocation to patient care, educational activities, and multitasking. Because the policy groups did not differ on time spent in different activity categories, we aggregated and analyzed time-motion data across all interns from the 6 training programs to comprehensively describe how interns spent their time on general medicine inpatient services. We then examined data from 4 periods of the day (morning, afternoon, evening, and night) to explore variations in how time was spent throughout the day. Finally, we quantified the proportion of time spent multitasking.

## Methods

### Study Design, Setting, and Participants

Time-motion data were collected from March 10 through May 31, 2016. The iCOMPARE programs were randomized to the 2011 Accreditation Council for Graduate Medical Education (ACGME) duty-hour policies (standard arm) or more flexible policies (flexible arm) that did not specify shift length limits or mandated lengths of time off between shifts for interns (eFigure 1 in Supplement 2).^12,13^ Participating programs included university-affiliated and community hospital training programs. Six programs, 3 in each policy group, were recruited for the time-motion substudy. The iCompare trial protocol is available in Supplement 1. The study was approved by the institutional review board at the University of Pennsylvania. All participants provided written informed consent.

Time-motion study programs were located in the mid-Atlantic region to facilitate in-person training of observers and ongoing monitoring of quality across multiple programs during the observation period. Each observer was assigned to 1 intern at a time and was scheduled so that an intern’s entire shift was observed while the intern was working on an inpatient general medicine (ie, nonspecialty) rotation. Observed shifts were limited to shifts starting on a weekday (Monday-Friday) and included daytime (short and long shifts as defined by the training program), nighttime, and call shifts (defined by the training program and typically lasting more than 14 hours). The proportion of shift types observed at each program reflected the program-specific distribution of shifts in terms of shift length and overnight schedules to capture a typical 24-hour weekday for each program. For example, for a given general medicine rotation (ranging from 2 weeks to 1 month) at a specific training program, if interns spent two-thirds of their rotation working day shifts and one-third working night shifts, the ratio of day to night shifts scheduled for observation at that program approximated 2:1. Programs had a variety of shift types with varying start and stop times (eFigure 3 in Supplement 2).

Interns rotating on the general internal medicine inpatient service at 6 mid-Atlantic teaching programs during the time-motion study period were eligible for observation. Interns, as opposed to more senior residents, were chosen for time-motion data collection because the 2 duty-hour policies studied in the iCOMPARE trial had different rules for intern shifts and different work hours for interns, and, generally speaking, interns are the primary, patient-facing physicians for patients receiving care from resident teams in teaching hospitals. Interns were recruited individually and in group settings (eg, during teaching conferences), and in person at each program. Among the 129 interns invited to participate, 120 (93.0%) consented, and 80 were included because they were on a general medicine service when observers were available. We did not record why interns chose not to participate.

### Data Collection Procedures and Measures

Data were collected by 23 observers (including A.M.Y. and J.M.A.). Observers received 4 hours of in-person training to collect data without interfering in interns’ daily workflow and to categorize interns’ activities based on prespecified categories (see below) using a custom-built tablet-based software (eFigure 2 in Supplement 2). We used 2 processes to assess observation reliability. First, after training was completed, each observer recorded activities observed while watching an 8-minute video of actors performing the predefined activities. The median κ coefficient among pairs of observers during training was 0.67. Second, during the study period, 10% of shifts were simultaneously observed by 2 observers. The median κ coefficient among paired observers during the study was 0.74.

The observer began recording activities when the intern arrived at the hospital and stopped when the intern finished their clinical duties for that shift. Shifts longer than 12 hours were typically split between 2 observers; precise split times were coordinated between observers based on their available schedule. As a quality check, we measured expected observation time based on interns self-reporting their arrival and departure times. In total, 97.7% of the total time expected to be observed was observed.

Activities were assigned to 7 major categories (Table 1), adapted from prior time-motion trials.^3,5^ The 7 major categories of activities were split into the following 2 sections: (1) the required section that indicated how an intern’s shift may be subdivided (ie, activities related to education, rounds, work, and handoffs), where at least 1 of 4 categories had to be recorded on the software or else a warning message would appear; (2) a not-required section that included direct patient care, indirect patient care, and miscellaneous (ie, nonpatient or non-clinical activities such as going to the restroom, eating, or sleeping). The not-required section activities were recorded as they occurred. The required section was purposefully incorporated to avoid unrecorded blank periods that could be interpreted as the intern not engaged in any activity or the observer forgetting to record. Three of the major categories–education, direct patient care, and indirect patient care–had subcategories.

More than 1 major or subcategory could be selected if different types of activities occurred simultaneously. For example, an intern could be recording the clinical encounter while talking to a patient. In this situation, the intern would be engaged in indirect patient care (subcategory of interacting with the health record) and direct patient care (subcategory of patient communication). We defined multitasking as periods when simultaneous activities in one of the following combinations of major categories were observed: (1) indirect and direct patient care; (2) indirect patient care and education; (3) direct patient care and education; (4) indirect patient care, direct patient care, and education; or (5) when 2 subcategories of indirect patient care (ie, interacting with the health record, communicating with team members, communicating with nonteam members, or viewing images) were observed because prior studies indicated these were dominant activities in a workday.^3-5^

### Statistical Analyses

Data were analyzed from June 1, 2016, through January 5, 2019. We used a granular approach to characterize time allocation to specific activities. For each second of a 24-hour day, we calculated the proportion of interns engaged in a specific activity by totaling the number of interns who were observed engaged in that activity and then dividing by the total number of interns who could have been observed during that period. For example, to calculate the proportion of interns spending time in direct patient care from 1:00:01 to 1:00:02 pm, we divided the total number of interns observed in direct patient care during that specific 1-second period by the total number of interns who were observed during that 1-second period (ie, an observer was on site). We repeated this procedure for every second of a 24-hour period for every recorded activity and then summed each second to a desired interval length, such as a 6-hour or a 24-hour period. When the percentage of interns engaged in a particular activity is summed across a set period and divided by that interval, each second is adjusted by the number of interns available to be observed. The summed value represents the mean proportion of time spent in a particular activity across all 6 training programs. This granular approach prevents oversampling of interns’ time, adjusting for time when they were not at work (ie, not observable) during a set interval.

We report the mean (SD) number of hours engaged in any recorded activity except work because direct and indirect patient care and their subcategories provide a more detailed description of work-related activities. We parsed our measurements based on the following 4 periods of the day: (1) morning (6 am to 12 pm), (2) afternoon (12 pm to 6 pm), (3) evening (6 pm to 12 am), and (4) night (12 am to 6 am). Clustering within programs was reflected using multilevel mixed-effects models with restricted maximum likelihood estimations using a random intercept for each program cluster.^14^ We calculated mean repeated observations of specific individuals occurring during the same time of day, accounting for within-person correlations A 2-sided *P* value of <.05 was considered statistically significant. All analyses were conducted using Stata software (version 14.1; StataCorp, LLP).

## Results

### Participant and Shift Characteristics

A total of 80 interns from 6 mid-Atlantic teaching programs were observed for 2173 hours. Forty-four (55.0%) of the interns were men, and 36 (45.0%) were women (mean [SD] age, 28.7 [2.3] years), 38 (47.5%) identified as white, and 30 (37.5%) identified as Asian (Table 2). We observed a median of 10.5 hours per shift (interquartile range [IQR], 9.6-12.5 hours). Among the 194 shifts observed, 120 (61.9%) were short daytime shifts (median length of observed time, 9.9 hours; IQR, 9.2-10.5 hours); 33 (17.0%) were long daytime shifts (median length of observed time, 12.2 hours; IQR, 11.0-12.6 hours); 35 (18.0%) were nighttime shifts (median length of observed time, 13.6 hours; IQR, 11.8-13.9 hours); and 8 (4.1%) were overnight call shifts (median length of observed time, 20.9 hours; IQR, 16.7-26.7 hours).

### Activities Within a 24-Hour Period

A mean (SD) of 15.9 (0.7) hours was spent in indirect patient care, reflecting 66% of the day (Table 3) that was mostly interns interacting with the patient’s medical record or recording their work (10.3 [0.7] hours), followed by communicating with core team members (5.9 [0.5] hours) and communicating with nonteam members about patients (3.3 [0.5] hours). Little time was spent viewing radiology images, electrocardiograms, or pathology results (0.3 [0.0] hours). The next most frequently observed activities were rounds (5.0 [0.6] hours; 21% of the day) and direct patient care (3.0 [0.1] hours; 13% of the day), which consisted mostly of communicating with patients (2.6 [0.1] hours). These observed activities were followed by educational activities (1.8 [0.3] hours; 7% of the day), consisting primarily of educational conferences (1.1 [0.2] hours). The least amount of time was spent handing off patient care responsibilities (0.8 [0.2] hours; 3% of the day).

### Activities Across Periods

With a few exceptions, the amount of time spent engaged in particular activities was consistent throughout the day (Table 3). Indirect patient care was the predominant activity during each of the 6-hour time periods (mean [SD] range, 3.3 [0.4] to 4.2 [0.2] hours). Afternoons revealed the least amount of time in direct patient care but the most in education. During evenings and nights, marginally greater time was spent in direct patient care compared with the afternoon period, and very little time was spent in educational activities.

### Multitasking

When multitasking did occur, the combination of direct and indirect patient care (mean [SD], 0.7 [0.1] hours; 3% of the day) and indirect patient care combined with education (0.5 [0.1] hours; 2% of the day) were the most common (Figure, A, and eTable 1 in Supplement 2). Viewed another way, a substantial portion of the time spent in direct patient care or educational activities occurred while performing indirect patient care. Across a 24-hour period, 23% of all direct patient care and 28% of educational activities occurred simultaneously when interns were engaged in indirect patient care. Simultaneous activities in subcategories of indirect patient care were also frequent (Figure, B and eTable 2 in Supplement 2). In particular, interacting with the medical record while communicating with medical teams was the most common, occurring a mean (SD) of 2.1 (0.2) hours (9%) of the day. Multitasking with 2 or more indirect patient care activities occurred for a mean (SD) of 3.8 (0.4) hours of the day (16%).

## Discussion

Our findings from the largest multi-institutional time-motion study of internal medicine interns, to our knowledge, provides an updated snapshot of an intern’s experience in the hospital. Interns are predominantly engaged in indirect patient care, with little variation over 24 hours. Notably, more than 10 hours (43%) of a 24-hour period were spent interacting with the electronic medical record. In contrast, little time was spent in educational activities or direct patient care. When interns were engaged in these activities, indirect patient care often co-occurred.

Internal medicine interns in our study spend a small proportion of time directly engaged with patients. In the 1990s, 25% of inpatient time was spent engaging with patients.^6^ Time-motion studies from 2010 to 2012 observed that the proportion had dropped to 9% to 12%.^3-5^ Our finding that 13% of observed time was spent in direct patient care is consistent with these findings. We add to these studies, first, by describing what occurs in the evenings and nights, whereas prior studies have mostly observed daytime activities.^3-5^ We found little difference in how time is allocated in the evenings and nights compared with the daytime. If anything, fewer hours are spent at patients’ bedsides relative to the morning period, and the least amount of educational activities was observed in the evening and night. These findings may concern educators because dedicated nighttime shifts have become more common after programs adapted to the ACGME’s 2011 duty-hour regulations.^15,16^ Efforts to enhance the educational experiences at night may be warranted.

Second, we provide measures of multitasking, which few studies of training programs have included.^3,5^ Multitasking is important to understand because it may reflect residents trying to compress their clinical and educational demands into a finite number of hours.^17,18^ Multitasking episodes may be challenging in learning environments because time spent engaged in multiple activities may be less efficient than focusing on each activity separately.^19^ In addition, although prior studies have measured multitasking as the co-occurrence of any activity,^4^ we measure and provide more targeted definitions of multitasking. Without a consensus definition of multitasking, we chose to report what we believe is most informative for policy makers, accreditation bodies, and educators^20-22^: (1) the overlap among the 3 major activities of direct patient care, indirect patient care, and education and (2) co-occurring indirect patient care activities. Although time spent multitasking could be interpreted as modest, whether it occurred too frequently or infrequently should be gauged by measures of interns’ educational experience and how the quality of patient care was affected, which we do not measure.

How the medical community responds to these observations will be important moving forward. As trainees report high rates of burnout and depression early in their career,^23,24^ understanding how their workday affects their health and well-being may be an important next step.^25-31^ If the allocation of time, including multitasking, is causally related to the propensity of trainees to develop burnout and depression, then our findings provide a critical baseline from which the medical community can judge future efforts designed to improve the work environment for internal medicine trainees.

### Limitations

Our study has several limitations. First, we limited our study to internal medicine interns. The distributions of activities of interns in other fields, such as surgery or psychiatry, are likely to be different, although many non-internal medicine residents spend a significant portion of their first year rotating on internal medicine inpatient services. Second, our study observed interns at 6 training programs, potentially limiting its generalizability. Nevertheless, we observed 2 and a half times as many hours of intern time and included 3 times as many programs as the next largest time-motion study in the United States.^3^ Third, we observed interns only during general medical inpatient rotations. Although these rotations are arguably the defining experience of a medicine internship, medical interns spend many months in other settings. Finally, these resuits are descriptive. We can observe how interns spend their time and we can compare that with distributions from the past, but no established standards identify what distribution of activities is best for the educational experience of interns or the quality of care patients receive.

## Conclusions

This analysis of time-motion data from a large cohort of interns across multiple US training programs reveals that interns spend substantial time in indirect patient care and little in face-to-face contact with patients or engagement in educational activities. Concluding that this distribution is a problem might be easy, reflecting an appealing and perhaps nostalgic view that the best way to care for patients and the best way to learn from them is with personal contact. A more agnostic view is that even if face-to-face engagement is essential, more may not be necessary given that so much of patient care now occurs in teams, is informed by diagnostic test reports, and is mediated through the work of others. Our results suggest those realities define how medical interns spend their time, and although we cannot be sure whether that is good or bad, our findings provide an essential baseline measurement for future efforts designed to improve the workday structure and experience of internal medicine trainees.

## Supplementary Material

Supplement 3

Supplement 2

Supplement 1

## Figures and Tables

**Figure.**
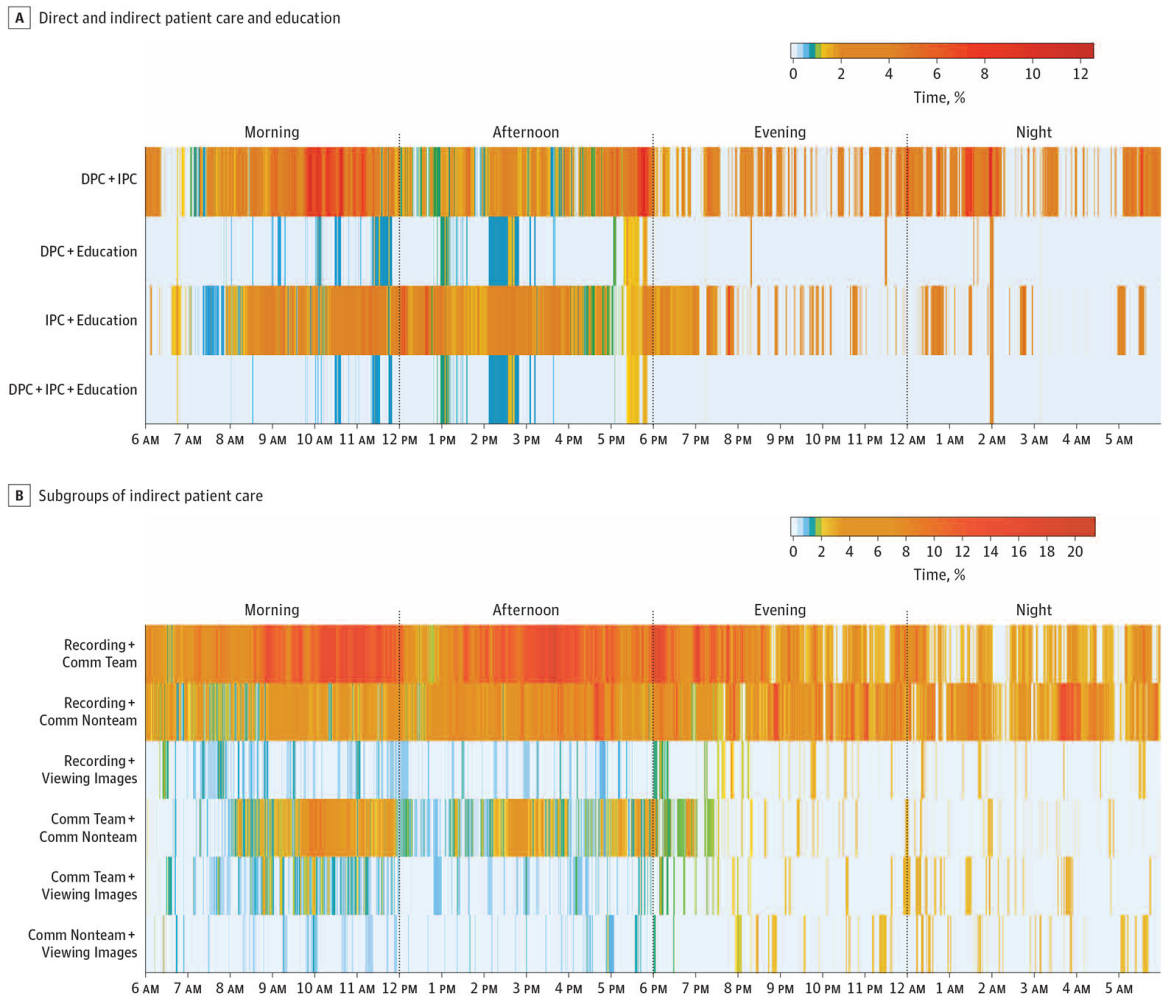
Heat Maps of Time Spent in Multitasking A, Each row represents a form of multitasking based on a combination of 2 or 3 of the following categories: direct patient care (DPC), indirect patient care (IPC), and education. Vertical lines within each row represent a 1-second interval within a 24-hour period. The intensity of the color represents the proportion of observed interns in a specific 1-second interval who were engaged in multitasking by completing 2 or more simultaneous activities (range, 0%-12% of observed interns). B, Each row represents a form of multitasking based on a combination of 2 of the following categories (all subgroups of IPC): communicating with nonteam members (Comm Nonteam), communicating with team members (Comm Team), interacting with medical record (Recording), and viewing image, electrocardiogram, pathology report, or other (Viewing Images). Vertical lines within each row represent a 1-second interval within a 24-hour period. The intensity of the color represents the proportion of observed interns in a specific 1-second interval who were engaged in multitasking by completing 2 simultaneous activities (range, 0%-20% of observed interns).

**Table 1.** Definitions of Time-Motion Categories and Activities

Category	Activity
Required^a^	
Education	Teaching or being taught^b^
	Educational conferences^b^
	Reading about medicine^b^
Rounds	Team rounds at bedside, hallway, or team room
Work	Any work time outside rounds, handoffs, or dedicated teaching (eg, calls to consultation teams, family meetings, procedures, or working in the medical record)
Handoffs	Transfers of patient care between interns
As many as applicable	
Direct patient care	Patient evaluation/management^b^
	Patient communication^b^
	Family communication^b^
	Other (eg, transporting a patient) ^b^
Indirect patient care	Interacting with medical record^b^
	Viewing image, electrocardiogram, pathology report, or other^b^
	Communicating with team^b^
	Communicating with nonteam members^b^
Miscellaneous	Non-work-related activities (eg, eating or using the bathroom)

a Indicates must pick at least one.

b Indicates subcategories for major categories.

**Table 2.** Characteristics of Interns Observed, Training Programs, Shift Types, and Shift Lengths

Characteristic	Values
Male, No. (%)	44/80 (55.0)
Age, mean (SD), y	28.7 (2.3)
Race/ethnicity, No. (%)	
White	38/80 (47.5)
Asian	30/80 (37.5)
Black/African American	1/80 (1.3)
Other	10/80 (12.5)
Missing	1/80 (1.3)
Training program type, No. (%)	
University-affiliated	4/6 (66.7)
Community	2/6 (33.3)
Shift type, No. (%)	
Day, short	120/194 (61.9)
Day, long	33/194 (17.0)
Night	35/194 (18.0)
On call	8/194 (4.1)
Shift length, median (IQR), h observed	
Daytime short	9.9 (9.2-10.5)
Daytime long	12.2 (11.0-12.6)
Nighttime	13.6 (11.8-13.9)
On call	20.9 (16.7-26.7)

Abbreviation: IQR, interquartile range.

**Table 3.** Distribution of Observed Activities Based on the Time of the Day^a,b^

	24-h Period		Morning (6 am to 12 pm)		Afternoon (12 pm to 6 pm)		Evening (6 pm to 12 am)		Night (12 am to 6 am)	
Observed Activity	Mean (SD), h	Proportion, %	Mean (SD), h	Proportion, %	Mean (SD), h	Proportion, %	Mean (SD), h	Proportion, %	Mean (SD), h	Proportion, %
Direct patient care	3.0 (0.1)	13	0.9 (0.1)	15	0.5 (0.1)	8	0.8 (0.1)	13	0.6 (0.1)	10
Patient evaluation or management	0.7 (0.1)	3	0.2 (0.0)	3	0.1 (0.0)	1	0.2 (0.0)	3	0.2 (0.0)	3
Patient communication	2.6 (0.1)	11	0.8 (0.1)	13	0.4 (0.1)	6	0.6 (0.1)	11	0.4 (0.1)	7
Family communication	0.5 (0.1)	2	0.1 (0.0)	1	0.1 (0.0)	3	0.2 (0.0)	3	0.1 (0.0)	1
Other (eg, transporting a patient)	0	0	0	0	0	0	0	0	0	0
Indirect patient care	15.9 (0.7)	66	4.1 (0.2)	69	3.8 (0.3)	63	4.2 (0.2)	70	3.3 (0.4)	54
Interacting with medical record	10.3 (0.7)	43	2.3 (0.1)	38	2.6 (0.3)	44	2.9 (0.3)	48	2.7 (0.3)	45
Viewing image, ECG, pathology report, or other	0.3 (0.0)	1	0.1 (0.0)	1	0	1	0.1 (0.0)	1	0.1 (0.0)	1
Communicating with team members	5.9 (0.5)	25	2.0 (0.2)	34	1.3 (0.2)	21	1.3 (0.3)	22	0.4 (0.1)	6
Communicating with nonteam members	3.3 (0.5)	14	0.8 (0.2)	14	0.8 (0.1)	14	1.1 (0.3)	19	0.7 (0.2)	12
Miscellaneous	1.8 (0.2)	8	0.3 (0.1)	6	0.4 (0.1)	6	0.6 (0.1)	10	1.5 (0.3)	25
Education	1.8 (0.3)	7	0.3 (0.1)	5	0.7 (0.2)	12	0.1 (0.0)	2	0.1 (0.0)	1
Teaching or being taught	0.5 (0.1)	2	0.2 (0.0)	3	0.2 (0.1)	3	0.1 (0.0)	1	0	0
Educational conferences	1.1 (0.2)	4	0.1 (0.1)	3	0.5 (0.1)	9	0	0	0	0
Reading about medicine	0.2 (0.0)	1	0	0	0	1	0.1 (0.0)	1	0.1 (0.0)	1
Rounds	5.0 (0.6)	21	2.3 (0.3)	38	0.3 (0.1)	4	0.4(0.3)	7	0.1 (0.1)	2
Handoffs	0.8 (0.2)	3	0.4(0.1)	7	0.5 (0.2)	8	0.6 (0.2)	11	0	0

Abbreviation: ECG, electrocardiogram.

a The percentage columns represent the proportion of time interns spent engaged in the particular activity in each row. For a 24-hour period, the denominator was 24 hours. For the morning, afternoon, evening, and night, the denominators were 6 hours.

b The sum of hours spent engaged in particular activities across all 4 periods approximate but do not equate the time spent engaged in particular activities across a 24-hour period. This phenomenon occurs because of our granular approach to characterize time allocation to specific activities. We observed interns on many different shifts throughout the day and accounted for those unable to be observed. For each second of a 24-hour period, we divided the number of interns engaged in a specific activity by the number of those who could have been observed (ie, interns were at work and an observer was present). We then summed across a specified interval (eg, 6 hours or 24 hours). The summed value represents the mean proportion of time spent in a particular activity across all 6 training programs. This granular approach prevents oversampling of interns’ time, adjusting for time when they were not at work (ie, not observable) during a set interval. Therefore, when calculating the mean (SD) and proportion of time spent in a particular activity, each 6-hour period and the 24-hour period have different denominators because they are sensitive to the dynamic number of interns observed in each second. We do not report values for the category of work because direct and indirect patient care and their subcategories provide a more detailed description of work-related activities.

## Key Points

### Question

How do current first-year internal medicine residents (interns) allocate time during inpatient training?

### Findings

In this secondary analysis of a cluster-randomized trial including 80 interns, participants spent more time caring for patients indirectly (eg, recording their work) than interacting with patients or in dedicated educational activities. Direct patient care or education frequently occurred when interns were simultaneously performing indirect patient care.

### Meaning

These findings provide a comparison with historical trends and a baseline measure for future efforts designed to improve the workday structure and experience of internal medicine trainees.

## References

[1] Bueno H, Ross JS, Wang Y, Trends in length of stay and short-term outcomes among Medicare patients hospitalized for heart failure, 1993-2006. JAMA. 2010;303(21):2141–2147. doi:10.1001/jama.2010.748 20516414 PMC3020983

[2] Kozma CM, Dickson M, Raut MK, Economic benefit of a 1-day reduction in hospital stay for community-acquired pneumonia (CAP). J Med Econ. 2010;13(4):719–727. doi:10.3111/13696998.2010.536350 21091099

[3] Block L, Habicht R, Wu AW, In the wake of the 2003 and 2011 duty hours regulations, how do internal medicine interns spend their time? J Gen Intern Med. 2013;28(8):1042–1047. doi:10.1007/s11606-013-2376-6 23595927 PMC3710392

[4] Mamykina L, Vawdrey DK, Hripcsak G. How do residents spend their shift time? a time and motion study with a particular focus on the use of computers. Acad Med. 2016;91(6):827–832. doi:10.1097/ACM.0000000000001148 27028026 PMC4879085

[5] Fletcher KE, Visotcky AM, Slagle JM, Tarima S, Weinger MB, Schapira MM. The composition of intern work while on call. J Gen Intern Med. 2012;27 (11):1432–1437. doi:10.1007/s11606-012-2120-7 22865015 PMC3475836

[6] Guarisco S, Oddone E, Simel D. Time analysis of a general medicine service: results from a random work sampling study. J Gen Intern Med. 1994;9(5): 272–277. doi:10.1007/BF02599655 8046530

[7] Gabow PA, Karkhanis A, Knight A, Dixon P, Eisert S, Albert RK. Observations of residents’ work activities for 24 consecutive hours: implications for workflow redesign. Acad Med. 2006;81(8):766–775. doi:10.1097/00001888-200608000-00016 16868436

[8] Leafloor CW, Lochnan HA, Code C, Time-motion studies of internal medicine residents’ duty hours: a systematic review and meta-analysis. Adv Med Educ Pract. 2015;6:621–629. 26604853 10.2147/AMEP.S90568 PMC4655905

[9] Lurie N, Rank B, Parenti C, Woolley T, Snoke W. How do house officers spend their nights? a time study of internal medicine house staff on call. N Engl J Med. 1989;320(25):1673–1677. doi:10.1056/NEJM198906223202507 2725617

[10] Asch DA, Bilimoria KY, Desai SV. Resident duty hours and medical education policy: raising the evidence bar. N Engl J Med. 2017;376(18):1704–1706. doi:10.1056/NEJMp1703690 28402246

[11] Institute of Medicine. Graduate Medical Education That Meets the Nation’s Health Needs. Washington, DC: National Academies Press; 2014. 25340242

[12] Shea JA, Silber JH, Desai SV, ; iCOMPARE Research Group. Development of the individualised Comparative Effectiveness of Models Optimizing Patient Safety and Resident Education (iCOMPARE) trial: a protocol summary of a national cluster-randomised trial of resident duty hour policies in internal medicine. BMJ Open. 2018;8(9): e021711. doi:10.1136/bmjopen-2018-021711 PMC6157525 30244209

[13] Desai SV, Asch DA, Bellini LM, ; iCOMPARE Research Group. Education outcomes in a duty-hour flexibility trial in internal medicine. N Engl J Med. 2018; 378(16):1494–1508. doi:10.1056/NEJMoa1800965 29557719 PMC6101652

[14] Rabe-Hesketh S, Skrondel A. Multilevel and Longitudinal Modeling Using Stata. 2nd ed. College Station, TX: Stata Press; 2008.

[15] Bolster L, Rourke L. The effect of restricting residents’ duty hours on patient safety, resident well-being, and resident education: an updated systematic review. J Grad Med Educ. 2015;7(3):349–363. doi:10.4300/JGME-D-14-00612.1 26457139 PMC4597944

[16] Association of Program Directors in Internal Medicine. APDIM Survey 2011. https://www.im.org/data/apdim-surveys. Accessed March 11, 2019.

[17] Tipping MD, Forth VE, O’Leary KJ, Where did the day go? a time-motion study of hospitalists. J Hosp Med. 2010;5(6):323–328. doi:10.1002/jhm.790 20803669

[18] Choi Y, Kim D, Chong H, Use of a 90-minute admission window and front-fill system to reduce work compression on a general medicine inpatient teaching service. J Grad Med Educ. 2017; 9(2):245–249. doi:10.4300/JGME-D-16-00211.1 28439362 PMC5398156

[19] Charron S, Koechlin E. Divided representation of concurrent goals in the human frontal lobes. Science. 2010;328(5976):360–363. doi:10.1126/science.1183614 20395509

[20] Gustin W, Batra R, Amin A, Rucker L. Education first: reforming the first-year curriculum of the internal medicine residency. Acad Med. 2009;84 (3):368–373. doi:10.1097/ACM.0b013e3181970cf5 19240448

[21] Boex JR, Leahy PJ. Understanding residents’ work: moving beyond counting hours to assessing educational value. Acad Med. 2003;78(9):939–944. doi:10.1097/00001888-200309000-00022 14507629

[22] Meltzer DO, Arora VM. Evaluating resident duty hour reforms: more work to do. JAMA. 2007;298 (9):1055–1057. doi:10.1001/jama.298.9.1055 17785652

[23] Shanafelt TD, Boone S, Tan L, Burnout and satisfaction with work-life balance among US physicians relative to the general US population. Arch Intern Med. 2012;172(18):1377–1385. doi:10.1001/archinternmed.2012.3199 22911330

[24] Rotenstein LS, Ramos MA, Torre M, Prevalence of depression, depressive symptoms, and suicidal ideation among medical students: a systematic review and meta-analysis. JAMA. 2016; 316(21):2214–2236. doi:10.1001/jama.2016.17324 27923088 PMC5613659

[25] Sinsky C, Colligan L, Li L, Allocation of physician time in ambulatory practice: a time and motion study in 4 specialties. Ann Intern Med. 2016; 165(11):753–760. doi:10.7326/M16-0961 27595430

[26] Shanafelt TD, Dyrbye LN, Sinsky C, Relationship between clerical burden and characteristics of the electronic environment with physician burnout and professional satisfaction. Mayo Clin Proc. 2016;91(7):836–848. doi:10.1016/j.mayocp.2016.05.007 27313121

[27] McMahon GT, Katz JT, Thorndike ME, Levy BD, Loscalzo J. Evaluation of a redesign initiative in an internal-medicine residency. N Engl J Med. 2010; 362(14):1304–1311. doi:10.1056/NEJMsa0908136 20375407

[28] Ratanawongsa N, Federowicz MA, Christmas C, Effects of a focused patient-centered care curriculum on the experiences of internal medicine residents and their patients. J Gen Intern Med. 2012; 27(4):473–477. doi:10.1007/s11606-011-1881-8 21948228 PMC3304041

[29] Ludmerer KM, Johns MM. Reforming graduate medical education. JAMA. 2005;294(9):1083–1087. doi:10.1001/jama.294.9.1083 16145029

[30] McMahon GT. Managing the most precious resource in medicine. N Engl J Med. 2018;378(16): 1552–1554. doi:10.1056/NEJMe1802899 29557706

[31] Alvin MD. iCOMPARE: an intern’s perspective. J Grad Med Educ. 2017;9(2):261–262. doi:10.4300/JGME-D-16-00711.1 28439371 PMC5398136

