# Supplementary material for: Assessment of Inpatient Time Allocation Among First-Year Internal Medicine Residents Using Time-Motion Observations
Source: JAMA Intern Med. Author manuscript; Available in PMC 2021 Sep 24. (PMC8462976; doi:10.1001/jamainternmed.2019.0095)
Supplement: Supplement 3 [file NIHMS1739779-supplement-Supplement_3.pdf]

Data Sharing Statement

Chaiyachati. Assessment of Inpatient Time Allocation Among First-Year Internal Medicine Residents Using Time-Motion Observations. *JAMA Intern Med*. Published April 15, 2019.
10.1001/jamainternmed.2019.0095

Data
Data available: Yes
Data types: Deidentified participant data
How to access data: The Data Coordinating Center for the iCOMPARE Trial will prepare de-identified datasets by the end of the funding period for deposit at the NHLBI BioLINCC repository (https://biolincc.nhlbi.nih.gov/home/).
When available: beginning date: 07-01-2022

Supporting Documents
Document types: None

Additional Information
Who can access the data: BioLINCC has an application process; information on how to apply for data is available at the BioLINCC website (https://biolincc.nhlbi.nih.gov/home/).
Types of analyses: For any purpose subject to the BioLINCC approval process.
Mechanisms of data availability: Without investigator support. Individuals can access the data repository after undergoing the BioLINCC approval process.
