# Supplementary material for: Assessment of Inpatient Time Allocation Among First-Year Internal Medicine Residents Using Time-Motion Observations
Source: JAMA Intern Med. Author manuscript; Available in PMC 2021 Sep 24. (PMC8462976; doi:10.1001/jamainternmed.2019.0095)
Supplement: Supplement 2 [file NIHMS1739779-supplement-Supplement_2.pdf]

Supplementary Online Content

Chaiyachati KH, Shea JA, Asch DA, et al. Assessment of inpatient time allocation among first-year internal medicine residents using time-motion observations. *JAMA Intern Med.* Published online April 15, 2019. doi:10.1001/jamainternmed.2019.0095

eFigure 1. PRISMA Diagram

eFigure 2. Images of Custom-Built Web-Based Program Used for Capturing Intern Activities

eFigure 3. Program-Specific Shift Types

eTable 1. Distribution of Multitasking Based on the Time of the Day

eTable 2. Distribution of Multitasking of Indirect Patient Care Based on the Time of the Day

This supplementary material has been provided by the authors to give readers additional information about their work.

© 2019 Chaiyachati KH et al. *JAMA Internal Medicine*.

eFigure 1. PRISMA Diagram

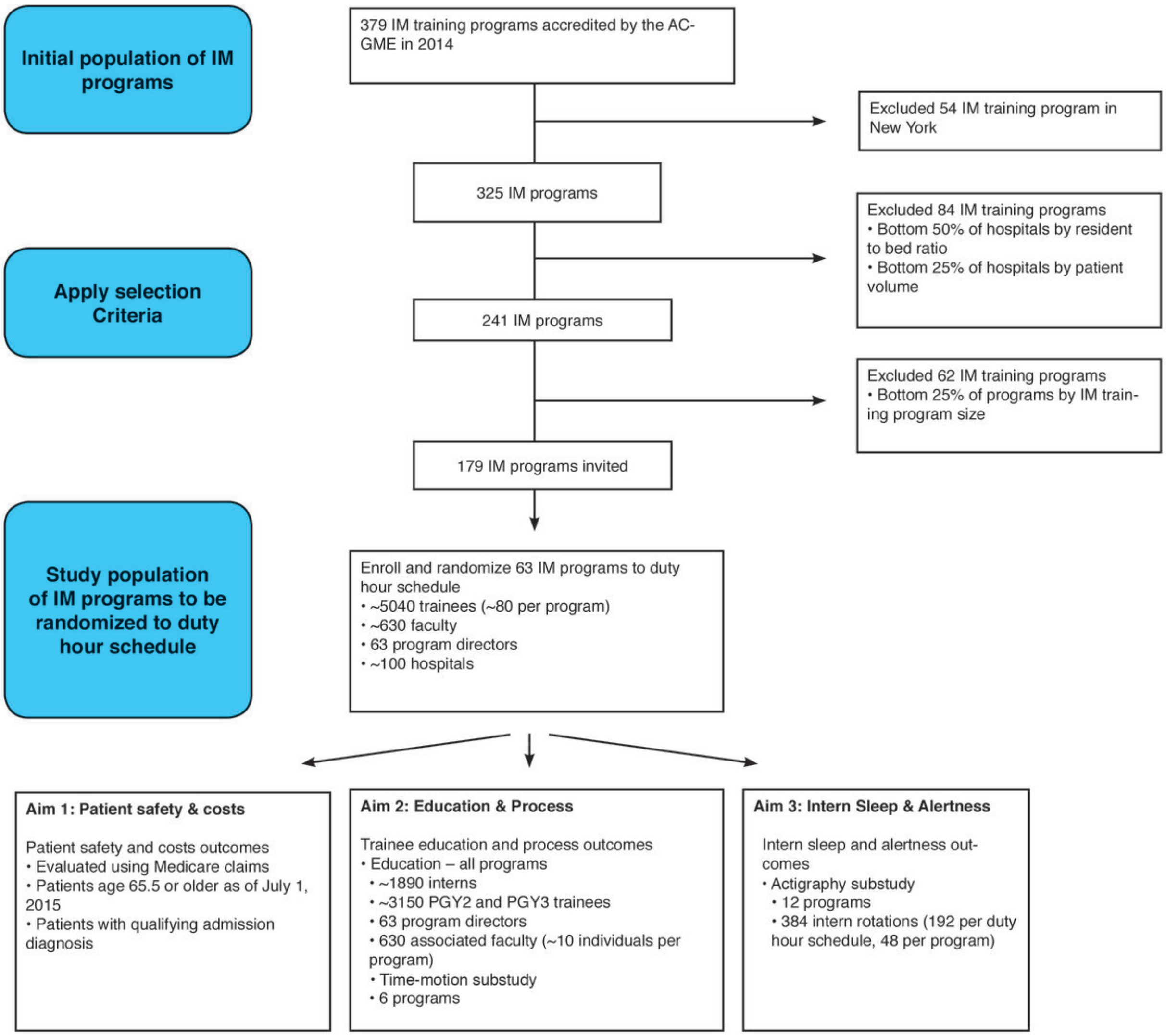

© 2019 Chaiyachati KH et al. *JAMA Internal Medicine*.

eFigure 2. Images of Custom-Built Web-Based Program Used for Capturing Intern Activities

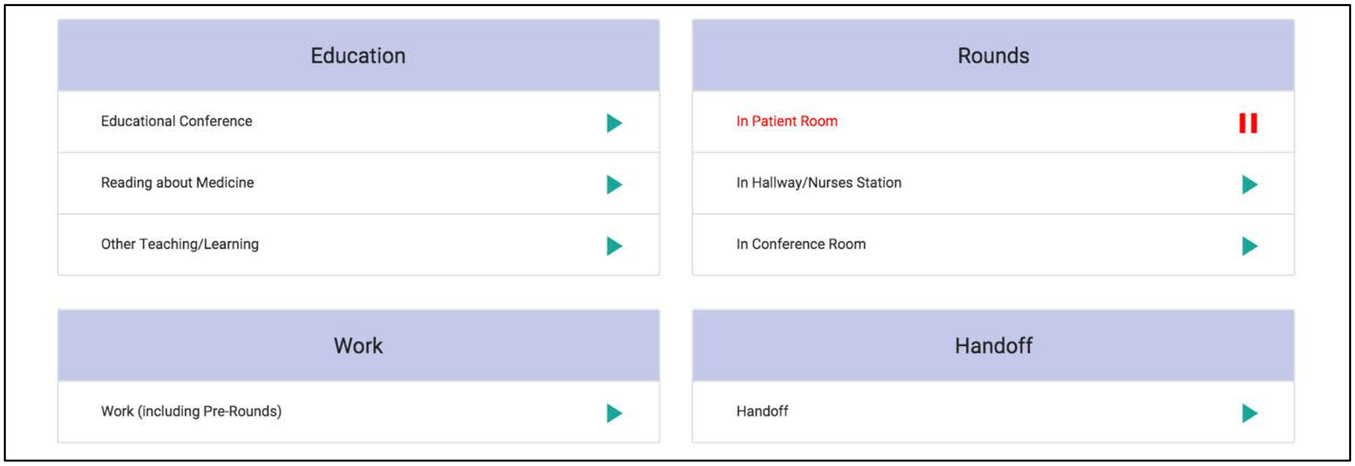

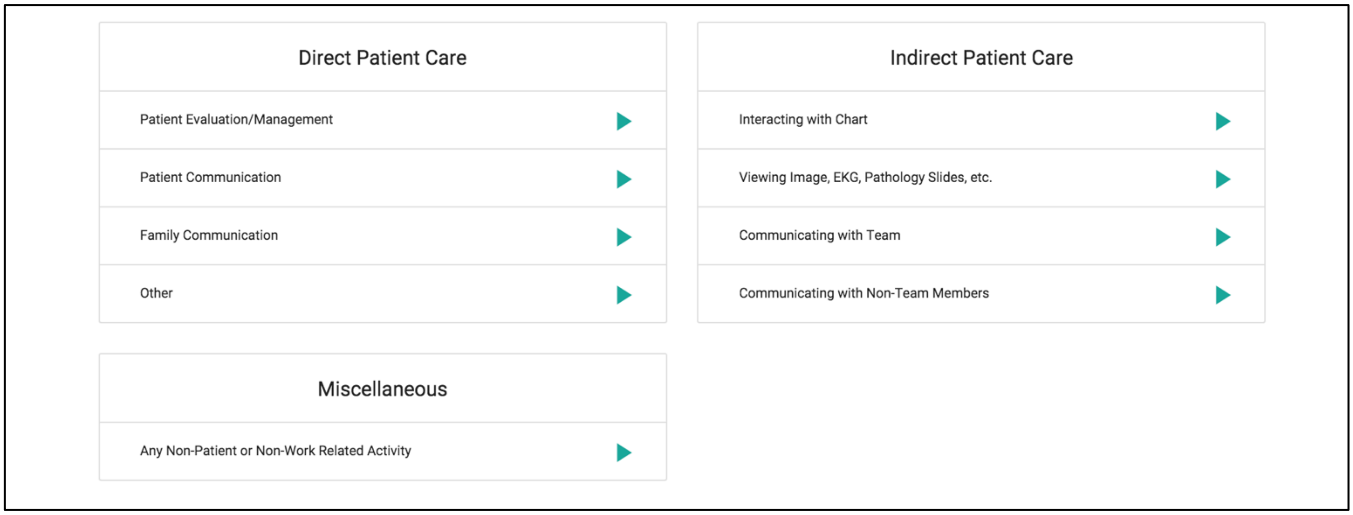

Note: Green arrows indicate an activity needs to be selected for it to be recorded. A red pause symbol indicates that an activity is being recorded and pushing pause would stop the recording. In these images, only "Rounds" is being recorded by capturing rounds occurring in the patient's room.

© 2019 Chaiyachati KH et al. *JAMA Internal Medicine*.

eFigure 3. Program-Specific Shift Types

Note: These panels describe the program-specific guidance for length of shifts and weekday activities (e.g., morning report, rounds, and conference) at each site. Program directors at each site self-reported this information. Sites 1-3 were from training programs without call shifts, corresponding to the standard arm in the individualized Comparative Effectiveness of Models Optimizing Patient Safety and Resident Education (iCOMPARE) trial. Sites 4-6 were from training programs with call shifts, corresponding to the flexible arm in the iCOMPARE trial.

Legend

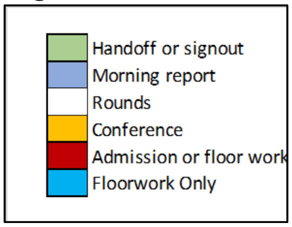

Panel A. Site 1

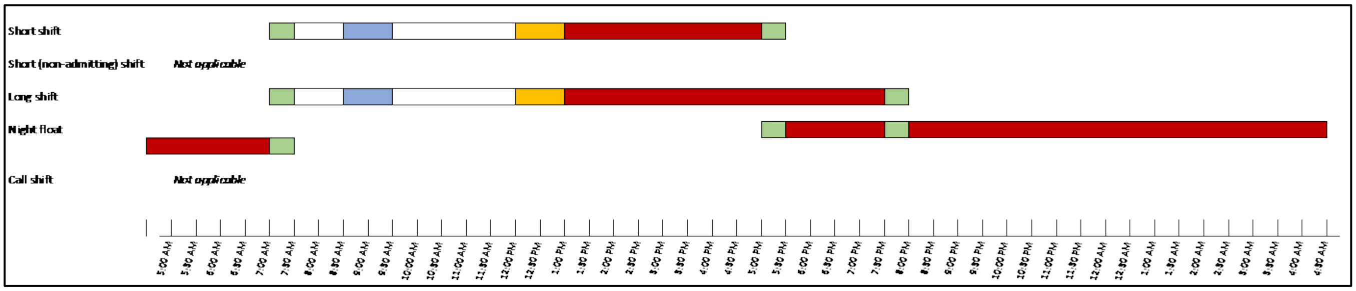

Panel B. Site 2

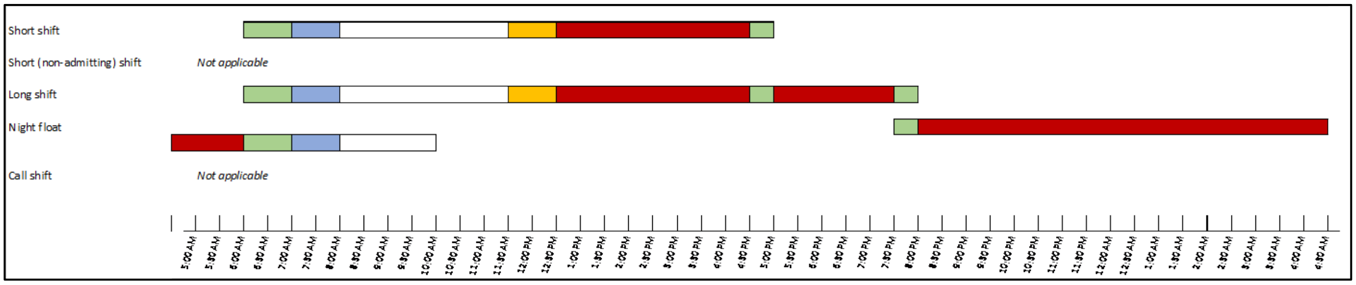

Panel C. Site 3

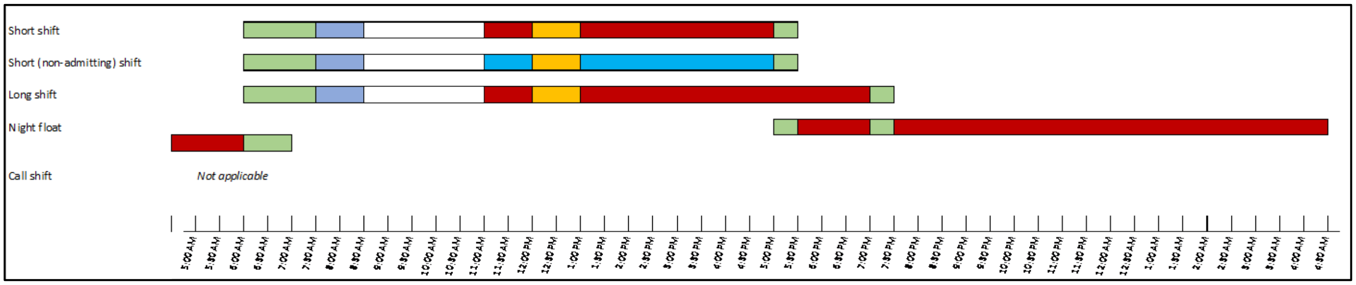

© 2019 Chaiyachati KH et al. *JAMA Internal Medicine*.

Panel D. Site 4

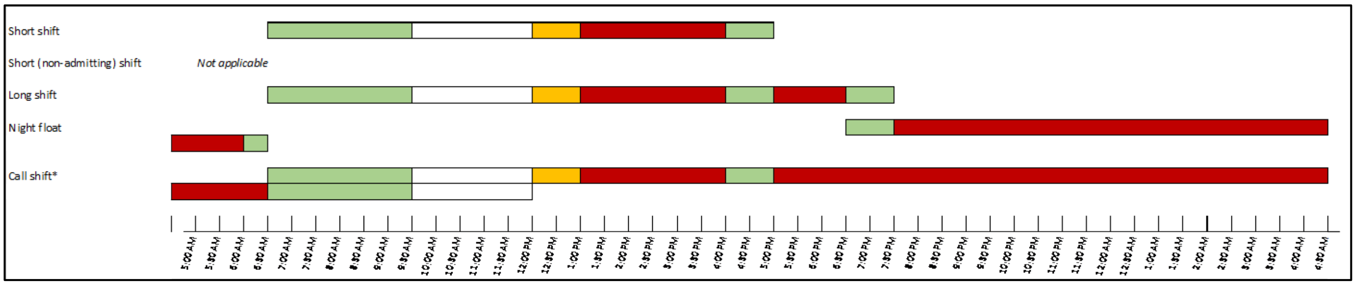

Panel E. Site 5

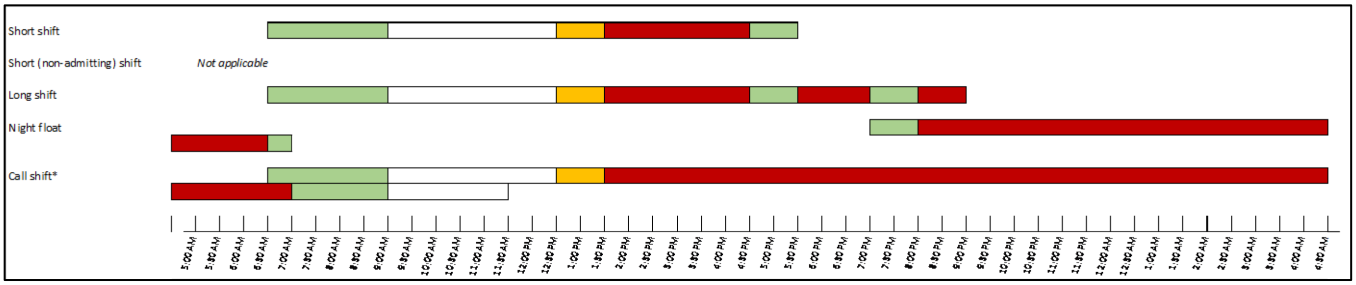

Panel F. Site 6

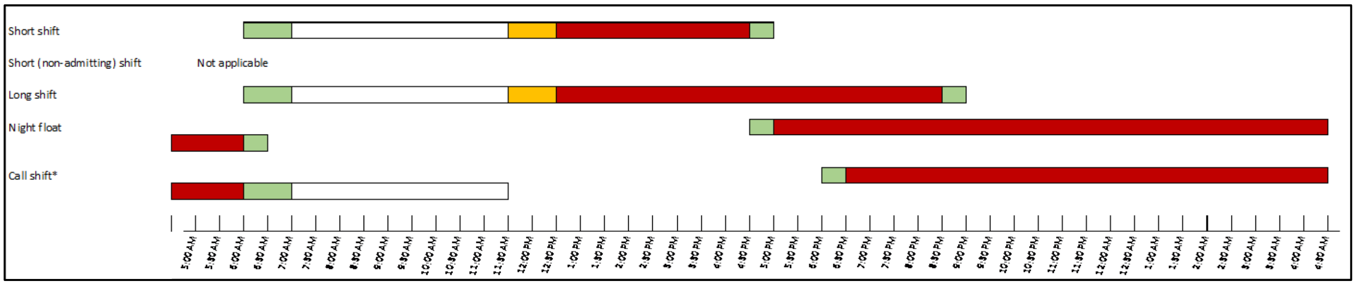

© 2019 Chaiyachati KH et al. *JAMA Internal Medicine*.

eTable 1. Distribution of Multitasking Based on the Time of the Day

	24-hour period		Morning (6A to 12P)		Afternoon (12P to 6P)		Evening (6P to 12A)		Night (12A to 6A)	
Observed Activities	Mean (SD) hours	%	Mean (SD) hours	%	Mean (SD) hours	%	Mean (SD) hours	%	Mean (SD) hours	%
Direct patient care (DPC) + Indirect Patient Care (IPC)	0.7 (0.1)	3	0.2 (0.0)	4	0.1 (0.0)	2	0.2 (0.1)	3	0.1 (0.1)	2
DPC + Education	0.0 (0.0)	0	0.0 (0.0)	0	0.0 (0.0)	0	0.0 (0.0)	0	0.0 (0.0)	0
IPC + Education	0.5 (0.1)	2	0.1 (0.0)	2	0.1 (0.0)	2	0.1 (0.0)	1	0.0 (0.0)	1
DPC + IPC + Education	0.0 (0.0)	0	0.0 (0.0)	0	0.0 (0.0)	0	0.0 (0.0)	0	0.0 (0.0)	0

Notes: The percentage columns represent the proportion of time interns spent engaged in particular multitasking activities which are represented by rows. For a 24-hour period the denominator was 24 hours. For the morning, afternoon, evening, and night time periods, the denominator was 6 hours. The sum of hours spent engaged in particular activities across all four time periods approximate but do not equate the time spent engaged in particular activities across a 24-hour period. This phenomenon occurs because we observed interns on many different shifts throughout the day. Therefore, the total number of observed interns varies each second of the day, impacting calculations for means and proportions.

© 2019 Chaiyachati KH et al. *JAMA Internal Medicine*.

eTable 2. Distribution of Multitasking of Indirect Patient Care Based on the Time of the Day

	24-hour period		Morning (6A to 12P)		Afternoon (12P to 6P)		Evening (6P to 12A)		Night (12A to 6A)	
Observed Activities	Mean (SD) hours	%	Mean (SD) hours	%	Mean (SD) hours	%	Mean (SD) hours	%	Mean (SD) hours	%
Interacting with health record (Recording) + Communicating with Team (Comm Team)	2.1 (0.2)	9	0.6 (0.1)	10	0.6 (0.1)	9	0.5 (0.1)	9	0.2 (0.1)	3
Recording + Communicating with Non-team Members (Comm Nonteam)	1.3 (0.3)	5	0.3 (0.1)	4	0.3 (0.0)	5	0.4 (0.2)	7	0.3 (0.1)	5
Recording + Viewing image, EKG, pathology, other (Viewing Image)	0.1 (0.0)	0	0.0 (0.0)	0	0.0 (0.0)	0	0.0 (0.0)	0	0.0 (0.0)	0
Comm Team + Comm Nonteam	0.4 (0.1)	2	0.1 (0.0)	2	0.1 (0.0)	1	0.2 (0.1)	3	0.0 (0.0)	0
Comm Team + Viewing Image	0.1 (0.0)	0	0.0 (0.0)	1	0.0 (0.0)	0	0.0 (0.0)	0	0.0 (0.0)	0
Comm Nonteam + Viewing Image	0.0 (0.0)	0	0.0 (0.0)	0	0.0 (0.0)	0	0.0 (0.0)	0	0.0 (0.0)	0

© 2019 Chaiyachati KH et al. *JAMA Internal Medicine*.
