# Supplementary material for: Assessment of Inpatient Time Allocation Among First-Year Internal Medicine Residents Using Time-Motion Observations
Source: JAMA Intern Med. Author manuscript; Available in PMC 2021 Sep 24. (PMC8462976; doi:10.1001/jamainternmed.2019.0095)
Supplement: Supplement 1 [file NIHMS1739779-supplement-Supplement_1.pdf]

This pdf contains the following items:

1. Original iCOMPARE protocol
2. Final iCOMPARE protocol
3. Summary of protocol changes
4. Final iCOMPARE statistical analysis plan (same as original; DSMB approved only 1 version)
5. Summary of statistical analysis plan changes

Protocol Details

Basic Info

Confirmation Number:	**bgegifai**
Protocol Number:	
Created By:	**SHEA, JUDY A**
Principal Investigator:	**ASCH, DAVID A**
Protocol Title:	**Individualized Comparative Effectiveness of Models Optimizing Patient Safety and Resident Education (iCOMPAR**
Short Title:	**iCOMPARE**
Protocol Description:	**iCOMPARE (Comparative Effectiveness of Models Optimizing Patient Safety and Resident Education) cluster randomized trial in a representative sample 58 Internal Medicine (IM) training programs that are randomly assigned to either the current duty hour standards (control programs) or less restricted flexible duty hour standards (intervention programs). Outcomes focus on patient safety assessed through claims data and education assessed through surveys.**
Submission Type:	**Social and Biological Sciences**

Resubmission*
No

Study Personnel

Principal Investigator

Name:	**ASCH, DAVID A**
Dept / School / Div:	**4239 - DM-General Internal Medicine**
Campus Address Mail Code	**6218**
Address:	**BLOCKLEY HALL<br>Room 1223**
City State Zip:	**PHILADELPHIA PA 191046021**
Phone:	**215-898-0102**
Fax:	
Pager:	
Email:	**ASCH@WHARTON.UPENN.EDU**
HS Training Completed:	**Yes**
Training Expiration Date:	**02/02/2017**
Name of course completed :	**CITI Protection of Human Subjects Research Training - ORA**

Study Contacts

Name:	**SHEA, JUDY A**
Dept / School / Div:	**4239 - DM-General Internal Medicine**
Campus Address Mail Code	**6021**
Address:	**BLOCKLEY HALL<br>423 GUARDIAN DR**
City State Zip:	**PHILADELPHIA PA 191046021**
Phone:	**215-573-5111**
Fax:	
Pager:	
Email:	**SHEAJA@MAIL.MED.UPENN.EDU**
HS Training Completed:	**Yes**
Training Expiration Date:	**12/09/2015**
Name of course completed :	**CITI Protection of Human Subjects Research Training - ORA**

Other Investigator

Name:	**SHEA, JUDY A**
Dept / School / Div:	**4239 - DM-General Internal Medicine**
Campus Address Mail Code	**6021**
Address:	**BLOCKLEY HALL<br>423 GUARDIAN DR**
City State Zip:	**PHILADELPHIA PA 191046021**
Phone:	**215-573-5111**
Fax:	
Pager:	
Email:	**SHEAJA@MAIL.MED.UPENN.EDU**
HS Training Completed:	**Yes**
Training Expiration Date:	**12/09/2015**
Name of course completed :	**CITI Protection of Human Subjects Research Training - ORA**

Responsible Org (Department/School/Division):

4239 - DM-General Internal Medicine

Key Study Personnel

Name:	**EVEN-SHOSHAN, ORIT**
Department/School/Division:	**PE-Pediatrics**
HS Training Completed:	**No**
Training Expiration Date:	
Name of course completed:	

Name:	**BELLINI, LISA M**
Department/School/Division:	**DM-Department of Medicine**
HS Training Completed:	**No**
Training Expiration Date:	
Name of course completed:	

Name:	**SILBER, JEFFREY H**
Department/School/Division:	**PE-Pediatrics**
HS Training Completed:	**No**
Training Expiration Date:	
Name of course completed:	

Name:	**DINGES, DAVID F**
Department/School/Division:	**PS-Unit for Experimental Psychiatry**
HS Training Completed:	**Yes**
Training Expiration Date:	**01/09/2015**
Name of course completed:	**CITI Protection of Human Subjects Research Training - ORA**

Name:	**BASNER, MATHIAS**
Department/School/Division:	**PS-Unit for Experimental Psychiatry**
HS Training Completed:	**Yes**
Training Expiration Date:	**11/02/2015**
Name of course completed:	**CITI Protection of Human Subjects Research Training - ORA**

Name:	**SMALL, DYLAN S**
Department/School/Division:	**Statistics**
HS Training Completed:	**Yes**
Training Expiration Date:	**05/28/2016**
Name of course completed:	**CITI Protection of Human Subjects Research Training - ORA**

Disclosure of Significant Financial Interests*
Does any person who is responsible for the design, conduct, or reporting of this research protocol have a **FINANCIAL INTEREST**?
No

Certification
I have reviewed the *Financial Disclosure and Presumptively Prohibited Conflicts for Faculty Participating in Clinical Trials* and the *Financial Disclosure Policy for Research and Sponsored Projects* with all persons who are responsible for the design, conduct, or reporting of this research; and all required Disclosures have been attached to this application.
Yes

Social and Biological Sciences

Study Instruments
Discuss the particulars of the research instruments, questionnaires and other evaluation instruments in detail. Provide validation documentation and or procedures to be used to validate instruments. For well know and generally accepted test instruments the detail here can be brief. More detail may be required

for a novel or new instrument. For ethnographic studies identify any study instruments to be used (i.e. for deception studies) and describe in detail where, when and how the study will be conducted and who or what are the subjects of study. Note: For more information on how to conduct ethical and valid ethnographic research, follow the link For oral histories or interviews provide the general framework for questioning and means of data collection. If interviews or groups settings are to be audio taped or video taped describe in detail the conditions under which it will take place. Include a copy of any novel or new test instruments with the IRB submission.
There will be two types of data for this study. 1) Medicare claims data - for the sites that are participating in this study we will analyze deidentified Medicare claims data for targeted diagnosis of patients seen within hospitals affiliated with the residency program. 2) Surveys - Direct data collection by iCOMPARE will include a beginning and an end of year survey sent to each of the participating program directors and all of the trainees at participating sites. Most of the other measures iCOMPARE will use to test education hypotheses are already collected as part of routine graduate medical education processes, and have essentially 100% data completion because they are required for program accreditation. Data collected outside of iCOMPARE and provided to iCOMPARE includes ITE (InTraining Examination) scores obtained from the ACP, data from the ACGMEs year end survey of trainees (interns, PGY2, PGY3), data from the ACGMEs year end survey of core faculty, and data from APDIMs fall survey of program directors.

Group Modifications
Describe necessary changes that will or have been made to the study instruments for different groups.
none

Method for Assigning Subjects to Groups
Describe how subjects will be randomized to groups.
Randomization is at the level of the Internal Medicine (IM) training program. The Data Coordinating Center (DCC) housed at Johns Hopkins University will randomize programs to control or intervention arms. Randomization will occur in the fall of 2014 to allow each program sufficient time to prepare training schedules. Assignment to duty hour schedule will be 1 to and there will be no stratification.

Administration of Surveys and/or Process
Describe the approximate time and frequency for administering surveys and/or evaluations. For surveys, questionnaires and evaluations presented to groups and in settings such as high schools, focus group sessions or community treatment centers explain how the process will be administered and who will oversee the process. For instance, discuss the potential issues of having teachers and other school personnel administer instruments to minors who are students especially if the content is sensitive in nature. Describe the procedure for audio and videotaping individual interviews and/or focus groups and the storage of the tapes. For instance, if audio tape recording is to be used in a classroom setting, describe how this will be managed if individuals in the class are not participating in the study. Explain if the research involves the review of records (including public databases or registries) with identifiable private information. If so, describe the type of information gathered from the records and if identifiers will be collected and retained with the data after it is retrieved. Describe the kinds of identifiers to be obtained, (i.e. names, social security numbers) and how long the identifiers will be retained and justification for use.
The only new surveys to be administered as part of the iCOMPARE are an end of year survey sent to each of the participating program directors and all of the trainees at participating sites. They will be brief (less than 5 minutes). Invitations will be sent by iCOMPARE project staff using an electronic option such as RedCAP or Survey Monkey. They will be administered in Spring 2015 as baseline and again in Spring 2016. The email requesting completion will include a reminder about iCOMPARE, a brief summary of the type of information about to be queried, a summary of how iCOMPARE will use and share the information, a statement about strategies for data and identity security, a statement about participation implying consent for COMPARE to use the data, and a statement that participation is voluntary. The email will also include a link to the data collection website. Data provided by an intern will be identified by a unique iCOMPARE intern ID number and code.

Data Management
Describe how and who manages confidential data, including how and where it will be stored and analyzed. For instance, describe if paper or electronic report forms will be used, how corrections to the report form will be made, how data will be entered into any database, and the person(s) responsible for creating and maintaining the research database. Describe the use of pseudonyms, code numbers and

how listing of such identifiers will be kept separate from the research data.
The survey data collected for the study will be collected via a web-based system. Data provided by an intern will be identified by a unique iCOMPARE intern ID number and code. A member of the Data Coordinating Center housed at Johns Hopkins University will be the sole person who is able to link program and person identity to the study id and arm. The master file of identities is limited to one used and will be password proteted, stored on a secure server, and storied separate from study data.

Radiation Exposure*
Are research subjects receiving any radiation exposure (e.g. X-rays, CT, Fluoroscopy, DEXA, pQCT, FDG, Tc-99m, etc.) that they would not receive if they were not enrolled in this protocol?
No

Human Source Material*
Does this research include collection or use of human source material (i.e., human blood, blood products, tissues or body fluids)?
No

CACTIS and CT Studies*
Does the research involve Center for Advanced Computed Tomography Imaging Services (CACTIS) and CT studies that research subjects would not receive if they were not part of this protocol?
No

CAMRIS and MRI Studies*
Does the research involve Center for Advanced Magnetic Resonance Imaging and Spectroscopy (CAMRIS) and MRI studies that research subjects would not receive if they were not part of this protocol?
No

Cancer Related research not being conducted by an NCI cooperative group*
Does this protocol involve cancer-related studies in any of the following categories?
No

Medical Information Disclosure*
Does the research proposal involve the use and disclosure of research subject's medical information for research purposes?
No

If the answer is YES, indicate which items is is provided with this submission:

Use of UPHS services*
Does your study require the use of University of Pennsylvania Health System (UPHS) services, tests or procedures*, whether considered routine care or strictly for research purposes?
No

Primary Focus*
Research on human data sets (e.g. medical records, clinical registries, existing research data sets, medical administrative data, etc.)

Protocol Interventions

	**Sociobehavioral (i.e. cognitive or behavioral therapy)**
	**Drug**
	**Device - therapeutic**
	**Device - diagnostic (assessing a device for sensitivity or specificity in disease diagnosis)**
	**Surgical**
	**Diagnostic test/procedure (research-related diagnostic test or procedure)**
	**Obtaining human tissue for basic research or biospecimen bank**
x	**Survey instrument**
	**None of the above**

The following documents are currently attached to this item:

There are no documents attached for this item.

Sponsors

Business Administrator

Name:	**MOLLI, PEGGY A**
Dept / School / Div:	**4239 - DM-General Internal Medicine**
Phone:	**215-898-1720**
Fax:	**215-898-9924**
Pager:	
Email:	**peggy50@pobox.upenn.edu**

Funding Sponsors

Name:	**ACCREDITATION COUNCIL FOR GRADUATE MEDICAL EDUCATION**
Type:	**UPENN Other Non-Profit Organizations**

Regulatory Sponsor

IND Sponsor

none

Industry Sponsor

None

Project Funding*
Is this project funded by or associated with a grant or contract?
Pending

Sponsor Funding
Is this study funded by an industry sponsor?
No

Status of contract

The following documents are currently attached to this item:

Grant Application (icompare_acgmeprotocolaugust2014.pdf)

Multi-Site Research

Other Sites

Site:	**Johns Hopkins University**
Contact:	
Pi:	**James Tonascia, PhD**
Mail:	**Department of Biostatistics**<br>**615 North Wolfe Street, Room W5025**<br>**Baltimore, MD 21205**
Phone:	**410-955-3704**
Email:	**Jtonasc1@jhu.edu**

Management of Information for Multi-Center Research

The iCOMPARE study investigators are organized into two distinct but collaborating centers, the Clinical Coordinating Center (CCC), at the University of Pennsylvania led by David Asch, and the Data Coordinating Center (DCC), at the Johns Hopkins University led by James Tonascia. Each of these centers has separate areas of responsibility but will work together to achieve the aims of the project. Each Center is going through their own institutional IRB. We include a list of the JHU partners and their CITI certificates as an appendix. The CCC will have primary responsibility to manage and implement the protocol; to recruit and manage the participating programs; to oversee the timely collection and quality control of relevant study data; to ensure compliance with IRB and other regulatory bodies; and to distribute supplies and funds as appropriate. The DCC will have primary responsibility to receive and manage all study data; to maintain a project website and facilitate project communications; to prepare interim and final reports of the studys progress and results; and to perform statistical analyses to support conclusions drawn from the study. The Centers will work together to establish and maintain quality assurance in the participating residency programs and to provide timely highquality publications of the studys results. A productive collaboration between the CCC and DCC toward a common mission has already developed. iCOMPARE also includes many other participants program directors, trainees, and faculty. The study has launched a website (iCOMPAREstudy.com) already being used to build a community among these groups and to facilitate program recruitment. Later, the website will host study news and a forum for participants to provide feedback on the process. We will use social media channels, including email, SMS, Twitter, and Facebook to push information individually or collectively about survey due dates, incentives, or other matters. The primary leadership body for the trial will be a Steering Committee composed of key investigators from both the CCC and the DCC. The Steering Committee will appoint a smaller Executive Committee to facilitate decision making. An Advisory Board will be assembled to provide additional scientific input and guidance. The Steering Committee (SC) is the principal decision-making body for iCOMPARE and will be chaired by David Asch, Principal Investigator of the CCC, with James Tonascia, PI of the DCC, serving as vice-chair. The remainder of the SC will be composed of core study team members from the CCC and the DCC. The SC will have the responsibility to approve the project protocol and any subsequent amendments and to vote on other important decisions. A quorum of the SC will be seven members with decisions made by agreement of a majority of those participating. It is expected that the SC will appoint subcommittees, possibly to include nonmembers of the SC, to make recommendations in areas such as protocol implementation issues, publications, and ancillary studies. The SC will meet monthly by teleconference or in person. The Executive Committee (EC) will manage day-to-day issues in iCOMPARE and will be chaired by Sanjay Desai of the CCC, with Judy Shea of the CCC serving as vice-chair. It will make decisions between SC meetings, and will organize and prepare agendas for the SC meetings. The EC will be composed of a sub group of the SC membership and will meet weekly by teleconference, although the frequency of meetings may vary depending on circumstance. We will not appoint a Data and Safety Monitoring Board (DSMB). The trial will produce no interim systematic data

on which to judge its safety until after the intervention has concluded. An Advisory Board (AB) will be appointed to make regular recommendations about the design and conduct of the project and will be chaired by Lisa Bellini of the CCC. The remaining AB members will not otherwise be part of the study team and will include the following leaders in graduate medical education, and policy: Vineet Arora, MD, MA Assistant Dean for Scholarship and Discovery at the University of Chicago and a national leader in medical education; Ruth Benca, MD, PhD Professor of Psychiatry at the University of Wisconsin and past President of the Sleep Research Society and the Associated Professional Sleep Societies. Karl Y Bilimoria, MD, MS a surgical oncologist at Northwestern who is leading an ACGME/ American Board of Surgery/American College of Surgeonsfunded study on resident duty hours in surgical training (FIRST trial); Patrick Conway, MD Deputy Administrator of the Center for Medicare and Medicaid Services (CMS) and Director of the CMS Innovation Center in the US Department of Health and Human Services; Michael ME Johns, MD Immediate past Chancellor of Emory University and Chair of the 2009 IOM Committee Report on Resident Duty Hours. The Advisory Board will make its reports directly to the Steering Committee. Program Directors from participating Internal Medicine residency programs represent site leaders for this multicenter trial. A deadline (fall 2014) will be set for receipt of application materials from candidate training programs. We require a minimum of 58 randomized programs to meet our statistical design specifications, but will accept and randomize more programs, if they apply and qualify by our deadline in fall 2014. Programs are submitting their applications now and thus cannot be listed. The CCC will host monthly conference calls for all participating program directors during the ramp up to the intervention start (Y1Q1) through the conclusion of the intervention period (Y2Q2) and ad hoc calls thereafter. Given the contributions required by participating program directors, efforts will be made to acknowledge them appropriately in publications as authors or other contributors as consistent with conventions and contributions. The Research Group for iCOMPARE consists of investigators and staff from the CCC and the DCC, members of the Advisory Board, the Program Directors of the participating training programs, study coordinators based at the programs, and faculty and trainees participating in the project. The CCC and the DCC will share responsibility for oversight and management of the participating residency programs. The CCC will have the primary role coordinating protocol implementation at each program, and will review intern duty schedules to ascertain compliance with the appropriate iCOMPARE intervention arm. The DCC will create and manage an internet-based data management system for the remote entry of surveys and any other data to be collected from the sites, and for later merging those data with those from other sources (such as CMS claims data and ACGME survey data). The DCC will also implement a data review system to perform quality assurance checks on data collected from the sites. As noted, the CCC will run monthly group calls with the program directors (and separate and weekly calls with site coordinators involved in the sleep and time motion evaluations). The CCC and DCC will together establish systems for monitoring protocol implementation and clinic performance, and for determining the composition and frequency of any for cause site visits.

The following documents are currently attached to this item:

There are no documents attached for this item.

Protocol

Abstract
In 2011, the Accreditation Council for Graduate Medical Education (ACGME) limited first year trainees (interns) to 16 hours of continuous duty. The implementation of these standards has raised safety concerns regarding fragmented continuity and increased patient handoffs between physicians, as well as educational concerns for trainees, given the same loss of continuity and the compressed time for learning and direct patient care. We propose the iCOMPARE study as the first national multicenter randomized controlled trial to address these pressing questions in internal medicine resident education. iCOMPARE is timed to complement the ongoing FIRST (Flexibility in Duty Hour Requirements for Surgical Training Trial) designed to evaluate more flexible duty hour scheduling in surgical resident education. iCOMPARE is designed as a cluster randomized trial in a representative sample 58 Internal Medicine (IM) training programs that are randomly assigned to either the current duty hour standards (control programs) or with less restricted flexible duty hour standards (intervention programs). We hypothesize that the intervention programs will experience (a) 30day patient mortality that does not exceed that observed under control programs and (b) educational outcomes (based on standardized testing) that are not inferior to those obtained in the control programs and satisfaction (as judged by

both faculty and trainees) that is higher than that reported in the control programs. This study has immediate policy relevance because it compares, in a highly naturalized setting, patient safety and education outcomes of switching from the current duty hour standards to a flexible approach tailored to local environments.

Objectives

Overall objectives

Since 2003, resident physician duty hours have been regulated across the US in the interest of reducing resident fatigue and promoting patient safety. Continuous duty hours for first year trainees (interns) were restricted further in 2011. However, recent studies have associated the 2011 standards with less direct patient contact, increased medical errors, increased transitions of care, decreased educational opportunities, and only modestly increased sleep. Program directors and trainees have expressed significant concern about the negative impact these rules have on patient safety and quality of training. And so it seems, what was intended as a way to reduce error by managing resident fatigue is now felt by others to promote error, by compressing schedules and increasing handoffs, and felt to decrease the educational opportunities and professionalization required to produce independent physicians. No existing research helps navigate resident duty hour policy between these competing considerations. The goal of the iCOMPARE study is to fill these gaps with specific aims listed below.

Primary outcome variable(s)

Specific Aim 1: Patient Safety Outcomes: Examine patient safety under the intervention and control conditions. We hypothesize: H1: 30day mortality under intervention conditions will not exceed (will not be inferior to) patient mortality under control conditions. Mortality as assessed via Mediciare claims data in the primary outcome variable.

Secondary outcome variable(s)

Specific Aim 2: Trainee Education Outcomes: Examine the quality of education under the intervention and control conditions. We hypothesize: H2a: Trainees in intervention conditions will have higher satisfaction with their educational experience than trainees in control conditions. H2b: Faculty in intervention conditions will have higher satisfaction with their clinical teaching experiences than faculty in control conditions. H2c: Faculty in intervention conditions will have a higher perception of safety, teamwork and supervision than faculty in control conditions. H2d: Standardized test scores for trainees in intervention conditions will not be lower than (will not be inferior to) those for interns Primary outcome variables will be satisfaction and perceptions gathered through surveys in Spring 2015 and 2016 and exam scores from Fall 2015 and 2016.

Background

A 1971 study that found fatigued interns tended to misinterpret electrocardiograms prompted discussion on duty hours, but no action [1]. The well-publicized death of Libby Zion prompted the first state-level regulation of duty hours in 1989 in New York [2]. Under increasing public and legislative pressure to restrict duty hours for graduate medical trainees, ACGME implemented duty hour standards for all accredited training programs effective July 1, 2003 [3]. These standards represented one of the largest national efforts ever undertaken to reduce errors in teaching hospitals. The intent of these standards was to improve patient safety; however, the preponderance of data after their implementation much of it produced by members of our team [4-16]demonstrated no definite benefit in safety, and concerns for increased risks[8,1015], and no clinically important improvements in Internal Medicine Board Scores subsequent to the 2003 reform [4]. Subsequently, and in response to a Congressional request, an IOM Committee was charged with making recommendations to optimize resident work hours to improve patient safety. In 2009, the IOM published its report recommending naps for any trainee working over 16h [17]. The ACGME then revised the national standards in 2011 mandating rest periods between duty periods, increased supervision for junior trainees, and a 16h limit on continuous duty hours for PGY1 trainees (interns)[18]. However, since the 2011 standards have been implemented, concerns have been raised regarding the impact on patient safety, trainee education, and health care costs. Studies have associated the new standards with less direct patient contact, increased medical errors, increased transitions of care, decreased educational opportunities, and only modestly increased sleep[19-21]. Furthermore, significant dissatisfaction has been reported by program directors and trainees about the negative impact on patient safety and quality of training [22-24]. Data from single center randomized clinical trials in internal medicine suggest some alternative models may be equal or superior in relevant patient and trainee outcomes. One study randomized internal medicine interns to a schedule with 16h

limits or 30h limits [20]. During the window on which interns were on their longest shifts (a 48h period comprising either the 16h shift or the 30h shift), interns on the 16h schedule slept approximately 3 hours more than interns on the 30hour schedule. However during a 4week clinical rotation, interns on the 16h schedule did not sleep significantly more on average than interns on the 30h schedule. Additionally, transitions in care were 130200% higher in the 16h schedule. These data make a compelling case that the current policies might be improved to meet the complex and competing needs of the public and the medical communities. iCOMPARE will use a cluster randomized design to compare two alternative duty hour standards for trainees in 58 programs in Internal Medicine for one year. The intervention programs will construct schedules that comply to the following duty hour standards: no more than 80 hours of work per week, one day off in seven (when averaged over four weeks), and in-house call no more frequently than every third night (when averaged over four weeks). The outcomes in the intervention programs will be compared to the outcomes from the control programs using schedules compliant with the current ACGMEmandated duty hour standards, which includes limitations in work duration to 16 hours for PGY1 trainees and 28 hours for PGY2,3 trainees. We will evaluate the extent to which the intervention programs preserves clinical and educational outcomes. [references provided as an attachment at the end]

Study Design

Phase*
Not applicable

Design
iCOMPARE is a cluster randomized trial comparing two alternative duty hour standards for interns in 58 programs in Internal Medicine. The intervention programs will have less restrictive standards and will be compared to the current ACGMEmandated standards, which limits work duration to 16 hours. We will evaluate the extent to which the intervention programs preserve clinical and educational goals. Randomization is at the level of the Internal Medicine (IM) training program. The Data Coordinating Center (DCC) will randomize programs to control or intervention standards. Randomization will occur in the fall of 2014 to allow each program sufficient time to prepare training schedules. Assignment to duty hour schedule will be 1:1 and there will be no stratification.

Study duration
The intervention is technically one year, AY 15-16 (July 2015-June 2016). Pretrial work such as recruitment and collection of baseline data will begin in Fall 2014 and analyses and writing will extend into 2018.

Resources necessary for human research protection
Describe research staff and justify that the staff are adequate in number and qualifications to conduct the research. Describe how you will ensure that all staff assisting with the research are adequately informed about the protocol and their research related duties. Describe access to a population that would allow recruitment of the targeted number of subjects. If medical or psychological services as a consequence of the research, describe how the subject will be referred to those services. Describe your facilities and justify that the facilities are adequate. Verify that there is sufficient time to conduct and complete the research.
The study team led by Dr. Asch is a group of accomplished researchers in safety and education. To assist the investigators (Asch, Shea, Basner, Bellini, Dinges, Silber, Small) and the DCC team we plan to hire a programmer to work primarily with Dr. Silber and his team on the Medicare claims data analyses and a project director to work primarily with Drs. Shea and Asch on the education outcomes and overall project administration. All investigators and support staff will maintain appropriate certifications and follow protocol procedures. In terms of recruitment, Drs. Bellini (from Penn) and Desai (from JHU) are internal medicine program directors who have visible roles int he national organization - Association of Program Directors in Internal Medicine (APDIM). They have been advertising the study at their national meeting and been received with much enthusiasm. The application process is well-underway. Most of the Medicare analyses (aim 1) will be done at CHOP under the direction of Dr. Silber and his team, using procedures and equipment standard for working with large Medicare claims data sets. Specifically the deidentified data will be housed in a secure password protected work environment accessible only by designated programmers and task-related investigators. The education collection and analyses (Aim 2) will be shared between Penn and the DCC at JHU. As above the deidentified data will be housed in a secure password protected work environment

accessible only by designated and task-related staff and investigators. Transfer of data between sites will be via Penn approved options such as DropBox.

Characteristics of the Study Population

Target population
For Aim 1 (safety) we will be using Medicare claims data. Our study population for evaluation of patient safety will be Medicare fee for service (FFS) beneficiaries at least age 65.5 at admission and admitted to one of the acute care hospitals affiliated with the 195 IM programs eligible for randomization to control or intervention duty hour standards between July 1, 2015 and June 30, 2016 with any of the eligible principal diagnoses -see attachement] . Data from before the intervention period will be used as a baseline for safety analyses before the paired data are available. Approximately 80% of hospital patients have claims from Medicare. The analyses are limited to FFS beneficiaries because CMS reports only Medicare fee for service patients. All FFS Medicare patients will have complete, linked data: inpatient (Medicare Part A), outpatient, physician (Medicare Part B), and associated denominator files. Patients will be included if they were not enrolled in a managed care program six months before admission and one month post discharge. The minimum age is 65.5 and FFS status in the 6 months prior to admission are required so that claims are available for 6 months prior to the qualifying admission. The diagnoses were chosen to reflect the vast majority of patients on the typical IM service. Examining all patients, rather than just Medicare aged FFS patients, would be ideal but is not feasible because 30day mortality, as well as the secondary outcomes, require linkable data to events occurring outside the hospital, something not practical to obtain outside the Medicare system (i.e., it would be impractical to consent patients to be able to see their data, or to rely on numerous insurance companies or various state databases due to the scale of this trial). No patients will be excluded based on gender or race/ethnicity. We expect very little change in year to year demographic and clinical characteristics of the patients admitted to each hospital as we observed in previous studies. Patients will not be recruited or contacted by iCOMPARE in any way for collection of data. Patient data will be obtained from CMS using the processes set up by CMS for leveraging their data for research purposes. For Aim 2 (education) we will recruit 58 internal medicine programs from the total of 379. New data for this study will some from the 58 program directors and the average of 80 trainees at each sites. We estimate a total of 4640 total trainees at the 58 participating IM programs, so with a 70% response rate we estimate at least 3248 trainee respondents. We assume there are 10 faculty per program so a total of 580; with a 60% response rate to surveys we expect 348 participants.

Subjects enrolled by Penn Researchers
160

Subjects enrolled by Collaborating Researchers
3436

Accrual
Our unit of analysis is the training program. We will recruit 58 of the 379 existing internal medicine programs.Sample size calculations were performed on the basis of mortality - the primary safety variable. The PASS 11 software is well suited for iCOMPARE since it has implemented the complex statistical calculations needed to allow for superiority or noninferiority hypotheses, and correlations in responses due to clustering on the IM programs. The primary hypotheses for aim 1 (mortality) is of the noninferiority type. Since the estimated 30day mortality rates (2008 data) in the iCOMPARE target population was 11%, the consensus noninferiority mortality margin among the iCOMPARE investigators was assumed to be 1%. We performed the calculations with both 80% and 90% power to gauge any gains in power by recruiting beyond the N=58 IM programs required for 80% power. While 80% power is acceptable for noninferiorty hypothesis testing, if we increase from 58 to 80 randomize programs the power rises to 90%. Since the sample sizes for aim 2 are at the program director or resident level, there will be more than adequate statistical power to detect effects as small as 0.25 SD in the target outcome measures. We will used mixed models as outlined in Diggle et al. (see Reference 56).

Key inclusion criteria
- Current internal medicine program in the United States Continued Accreditation status with the ACGME - Program director agrees to: 1. randomization to one of the two study arms. 2. develop, and share with the study team, institutional duty hour schedules and regulatory policies/procedures that will ensure adherence to, and enforcement of, the duty hour regulations that apply to their study arm 3. allow access and analysis of de-identified resident duty hour adherence data/logs, call schedules, and rotation schedules to the Study Team. 4. participate, and encourage trainee participation at your institution, in beginning and end of year iCOMPARE surveys.

Key exclusion criteria
We exclude 119 programs that comprise the bottom 50% in resident-to-bed ratio and the bottom 25% in patient volume related to the diagnoses by which the patient population will be selected for evaluation of safety outcomes . Within the 260 programs that remain, we exclude the 65 in the lowest quartile of program size to ensure we can feasibly obtain sufficient trainee measurements. The 195 remaining programs are eligible for inclusion

Vulnerable Populations

Children Form

Pregnant women (if the study procedures may affect the condition of the pregnant woman or fetus) Form

Fetuses and/or Neonates Form

Prisoners Form

Other

x **None of the above populations are included in the research study**

The following documents are currently attached to this item:

There are no documents attached for this item.

Populations vulnerable to undue influence or coercion
none

Subject recruitment
Subjects for aim 1 (safety) are Medicare Fee-For-Service recipients seen as inpatients in the hospitals affiliated with the 58 study programs who had one of the relevant diagnoses. Patients will not be recruited or contacted by iCOMPARE in any way for collection of data. Patient data will be obtained from CMS using the processes set up by CMS for leveraging their data for research purposes. Subjects for aim 2 (education) are the program directors and associate program directors, and trainees at the 58 participating programs. Program directors will agree to participate on a voluntary basis. The materials for recruitment are attached. On two occasions (spring 2015 and 2016) we will invite trainees in these 58 programs to voluntarily take a survey. Completion of the assessment will be voluntary and provision of data will be considered to be provision of consent. We will emphasis the data will be part of a deidentified dataset and that no individual level data will go to program directors or anyone else. We will make it clear that we may link data at the individual level from our multiple data sources (those talks are in progress).

Will the recruitment plan propose to use any Penn media services (communications, marketing, etc.) for outreach via social media avenues (examples include: Facebook, Twitter, blogging, texting, etc.) or does the study team plan to directly use social media to recruit for the research?
Yes

Please identify which method(s) of social media you will utilize, the content of the text to be used, and the method(s) for posting this information (i.e., using Penn supported communication services). When proposing the text to utilize, please be aware of any social media limitations (i.e., number of characters allowed in a tweet) and any appropriate confidentiality practices necessary to be compliant with posting research recruitment text.*

The study has launched a website (iCOMPAREstudy.com) already being used to build a community among these groups and to facilitate program recruitment. Later, the website will host study news and a forum for participants to provide feedback on the process. We will use social media channels, including email, SMS, Twitter, and Facebook to push information individually or collectively about survey due dates, incentives, or other matters.

The following documents are currently attached to this item:

Subject recruitment (icomparedioandpdforms_101.docx)

Subject compensation*
Will subjects be financially compensated for their participation?
No

The following documents are currently attached to this item:

There are no documents attached for this item.

If there is subject compensation, provide the schedule for compensation per study visit or session and total amount for entire participation, either as text or separate document

Study Procedures

Procedures
iCOMPARE is a cluster randomized trial comparing two alternative duty hour standards for interns in 58 programs in Internal Medicine. The intervention programs will have less restrictive standards and will be compared to the current ACGMEmandated standards, which limits work duration to 16 hours. We will evaluate the extent to which the intervention programs preserve clinical and educational goals. Randomization is at the level of the Internal Medicine (IM) training program. The Data Coordinating Center (DCC) will randomize programs to control or intervention standards. Randomization will occur in the fall of 2014 to allow each program sufficient time to prepare training schedules. Assignment to duty hour schedule will be 11 and there will be no stratification. The ACGME has agreed to waive duty hour standards for participating programs. During the application process, each program must identify the hospitals affiliated with the program. All hospitals covered by a participating program are expected to participate. Exceptions are limited to hospitals that are covered by more than one participating program. A deadline (fall 2014) will be set for receipt of application materials from candidate training programs. The 58 selected programs are expected to include 4640 internal medicine residents: 1740 interns (approximately 30 interns per program) and approximately 1450 PGY2 trainees (approximately 25 PGY2 per program) and 1450 PGY3 trainees (approximately 25 PGY3 per program). Each program will include one program director (total of 58) and approximately 10 associated faculty (total of about 580 faculty). We will supplement the iCOMPARE data collected directly program from program directors with data collected on individuals and programs by national organizations (ACGME, ACP and APDIM). Patient safety (30day mortality) will be derived from Medicare claims database using methods proven in many other outcomes research studies by Drs. Silber, Small and his iCOMPARE team. The ACGME would grant programs randomized to the intervention arm waivers from the current duty hour standards. The three remaining standards would include: a. 1 day off in 7 averaged over 4 weeks, b. maximum of 80 hours/week of work, and c. inhouse overnight call no more frequent than q3 nights. All trainees rotating on services in the participating program would also be permitted to follow the duty hour rules of the internal medicine program. This includes rotators from other internal medicine programs as well as rotators from other departments, e.g. emergency medicine. To prevent the need for multiple programmatic schedule changes for intervention programs, and thus make the study feasible, the duty hour waivers will extend beyond the intervention year until action is taken by the ACGME on the duty hour policy. If action is not taken by 30 June 2019, the waivers will expire. Data are expected from the iCOMPARE study in calendar year 2018.

The following documents are currently attached to this item:

There are no documents attached for this item.

Analysis Plan
The 30day mortality outcome measure is defined, for each IM program, as the difference between 30day mortality rate in the trial year minus the 30day mortality rate in the pretrial year. This approach permits the use of a simple model (twosample ttest) for the set of N=29 pairs of test year vs. pretest year differences in each group (intervention vs. test) in annual 30day mortality that obviates the need for complex risk adjustment models, since in adjusts each outcome for secular trends in 30day mortality as well as IM program population risk profiles that are likely to cancel out comparing successive years. Similar between arm comparisons will be made for the education outcomes.

The following documents are currently attached to this item:

There are no documents attached for this item.

Deception
Does your project use deception?
No

Are you conducting research outside of the United States?
No

Data confidentiality

x **Paper-based records will be kept in a secure location and only be accessible to personnel involved in the study.**

x **Computer-based files will only be made available to personnel involved in the study through the use of access privileges and passwords.**

x **Prior to access to any study-related information, personnel will be required to sign statements agreeing to protect the security and confidentiality of identifiable information.**

x **Wherever feasible, identifiers will be removed from study-related information.**

A Certificate of Confidentiality will be obtained, because the research could place the subject at risk of criminal or civil liability or cause damage to the subject's financial standing, employability, or liability.

A waiver of documentation of consent is being requested, because the only link between the subject and the study would be the consent document and the primary risk is a breach of confidentiality. (This is not an option for FDA-regulated research.)

x **Precautions are in place to ensure the data is secure by using passwords and encryption, because the research involves web-based surveys.**

Audio and/or video recordings will be transcribed and then destroyed to eliminate audible identification of subjects.

Subject Confidentiality
For study aim 1 (patient safety), the analyses will be restricted to deidentified Medicare analytic files. For study aim 2 (education) the greatest risk to participants is the risk to confidentiality. Individually identifiable information will be available only for the patients who are Medicare beneficiaries. Individuallevel data for patients will be kept confidential and stored only on the highly secure servers available for patientlevel data at the main DCC site (Johns Hopkins). No data will be stored on PCs or laptops. Only authorized project personnel will have access to the data as overseen by the DCC. All data will be reported at units of aggregation which make impossible the identification of individual patients, residents, or faculty.

Sensitive Research Information*
Does this research involve collection of sensitive information about the subjects that should be excluded from the electronic medical record?
No

Subject Privacy
Privacy refers to the person's desire to control access of others to themselves. Privacy concerns people, whereas confidentiality concerns data. Describe the strategies to protect privacy giving consideration to the following: The degree to which privacy can be expected in the proposed research and the safeguards that will be put into place to respect those boundaries. The methods used to identify and contact potential participants. The settings in which an individual will be interacting with an investigator. The privacy guidelines developed by relevant professions, professional associations and scholarly disciplines (e.g., psychiatry, genetic counseling, oral history, anthropology, psychology).
There will be no contact with the Medicare beneficiaries in who deidentified data are analyzed for study aim 1. Program directors will be directly recruited via email invitations in and announcements at professional meetings in Fall 2014. Once they apply and are found to meet study criteria they will be randomized. At the beginning of the year we will ask them for program related data - a roster of their trainees with email addresses to enable direct invitations for surveys, a schedule for the rotations that will be included in the study, a list of associate program directors and other key education faculty and a contact person who can be contacted once a quarter to discuss any program or logistical changes. At the end of academic year 14-15 and 15-16 we will send electronic invitations to program directors, key faculty and trainees to complete a survey. Nonresponders will get two email reminders.

Data Disclosure
Will the data be disclosed to anyone who is not listed under Personnel?
no

*Data Protection**

	**Name**
x	**Street address, city, county, precinct, zip code, and equivalent geocodes**
x	**All elements of dates (except year) for dates directly related to an individual and all ages over 89**
	**Telephone and fax number**
x	**Electronic mail addresses**
	**Social security numbers**
	**Medical record numbers**
	**Health plan ID numbers**
	**Account numbers**
x	**Certificate/license numbers**
	**Vehicle identifiers and serial numbers, including license plate numbers**
	**Device identifiers/serial numbers**
	**Web addresses (URLs)**
	**Internet IP addresses**
	**Biometric identifiers, incl. finger and voice prints**
	**Full face photographic images and any comparable images**
	**Any other unique identifying number, characteristic, or code**
	**None**

Does your research request both a waiver of HIPAA authorization for collection of patient information and involve providing Protected Health Information ("PHI") that is classified as a "limited data set" (city/town/state/zip code, dates except year, ages less than 90 or aggregate report for over 90) to a recipient outside of the University of Pennsylvania covered entity?
Yes

The following documents are currently attached to this item:

There are no documents attached for this item.

Tissue Specimens Obtained as Part of Research*
Are Tissue Specimens being obtained for research?
No

Tissue Specimens - Collected during regular care*
Will tissue specimens be collected during regulator clinical care (for treatment or diagnosis)?
No

Tissue Specimens - otherwise discarded*
Would specimens otherwise be discarded?
No

Tissue Specimens - publicly available*
Will tissue specimens be publicly available?
No

Tissue Specimens - Collected as part of research protocol*
Will tissue specimens be collected as part of the research protocol?
No

Tissue Specimens - Banking of blood, tissue etc. for future use*
Does research involve banking of blood, tissue, etc. for future use?
No

Genetic testing
If genetic testing is involved, describe the nature of the tests, including if the testing is predicative or exploratory in nature. If predictive, please describe plan for disclosing results to subjects and provision of genetic counseling. Describe how subject confidentiality will be protected Note: If no genetic testing is to be obtained, write: "Not applicable."
Not applicable

Consent

1. Consent Process

Overview
Individuals consent for direct iCOMPARE data collection: Subjects are assumed to provide consent upon completion of the iCOMPARE surveys. Individuals consent for iCOMPAREs use of data originally provided to ACP, ACGME, and APDIM: We require only data identifiable at the level of the program. No individual will be linked to his or her data. Programs need not be identified except insofar as we can allocate them to arm; however, we will retain program identifiers in case events require us to remove specific programs from analysis. Consent issues related to patient data: Waiver of patient informed consent and HIPAA consent will be requested, for three reasons. First, since the trial will involve thousands of patients, it would not be feasible to consent patients. Second, the risk to patients from participation is minimal. earlier retrospective analyses conducted by members of the current team demonstrated no difference in patient outcomes with even longer duty shifts than those tested in iCOMPARE. Third, the data are not collected directly from patients; all patient data will be obtained from Medicare claims, requiring no active involvement by the patient.

Children and Adolescents
Not applicable

Adult Subjects Not Competent to Give Consent
Not applicable

2. Waiver of Consent

Waiver or Alteration of Informed Consent*
Waiver of written documentation of informed consent: the research presents no more than minimal risk of harm to subjects and involves no procedures for which written consent is normally required outside of the research context

Minimal Risk*

Impact on Subject Rights and Welfare*

Waiver Essential to Research*

Additional Information to Subjects

Written Statement of Research*
No

If no written statement will be provided, please provide justificiation

The proposed research is retrospective, involves thousands of physicians and hundreds of thousands of patients, poses only a minimal risk for physician or patient confidentiality and could not be practically executed if informed consent were required, consistent with Section 46.116(d). As such, we will obtain a waiver of informed consent regarding surgeons and patients from the Institutional Review Board at the University of Pennsylvania

The following documents are currently attached to this item:

There are no documents attached for this item.

Risk / Benefit

Potential Study Risks
The greatest risk to participants is the risk to confidentiality. For patients, we use Medicare claims data to analyze clinical outcomes. Analysis of these administrative data, which are routinely collected, is felt to be the least intrusive method of measuring these outcomes and, given high standards of information security described below, also the most secure. For trainees in general, we will see educational assessment scores that are individually deidentified and so should present little to no risk to confidentiality. For faculty, no information is collected that is individually identifiable. Lastly, all program directors will communicate to their trainees that participation in the iCOMPARE surveys will have zero effect on their trainee assignments or evaluations. Additionally, program directors will not know the survey completion status or responses of individual trainees

Potential Study Benefits
There are no substantial direct benefits to research participation other than contributing to the development of new knowledge as described below. However, risks are also low and managed. If the hypotheses are supported, the policy changes that are expected to result will be of benefit to patients, faculty, and trainees. This study has immediate policy relevance because it compares, in a highly naturalized setting, sleep, education, and safety outcomes of potential duty hour reform. The implementation of these standards has raised safety concerns associated with fragmented continuity and increased patient handoffs between physicians, as well as educational concerns for trainees, given the same loss of continuity and the compressed time for learning and direct patient care. Because physician training affects not only the care of patients in teaching hospitals today, but also the care of all of the future patients of the graduating trainees as they move into independent practice, training policy has a potentially highly leveraged and enduring impact on health care quality.

Alternatives to Participation (optional)
Program directors can choose not to participate in the study. If a program is in the study, program directors, faculty and trainees can choose to not complete the iCOMPARE administered surveys. However, their data collected by ACP and ACGME would be part of the program level data as we do

not have individual identifiers and are not able to delete it.

Data and Safety Monitoring
The Steering Committee will establish a procedure for timely reporting and addressing of ad hoc reports of safety issues or concerns arising as the trial proceeds

The following documents are currently attached to this item:

There are no documents attached for this item.

Risk / Benefit Assessment
The immediate risks of this study for patient, program director and resident participants are minimal. Potential benefits are described above. Overall the risk benefit ratio is favorable given the long term potential of this study to significantly contribute to our knowledge of the impact of duty hour rules on education, and patient outcomes. All trainees in participating programs will be included; thus, women and minorities will be included according to their representation in these populations. All trainees are over the age of 18.

General Attachments

The following documents are currently attached to this item:

Questionnaires (eoytrainees_views_icompare_012514.docx.doc)

Questionnaires (eoy_pdsurvey_icompare_012514.doc)

Recruitment materials (icomparedioform_101.docx)

Additional forms (citi_shaded.pdf)

Additional forms (citi_tonasciaj.pdf)

Cover Letter (aschtomeagherreicompare10114.docx)

Recruitment materials (icomparepdenrollmentform-10114.docx)

Additional forms (jhu_dcclist_100214.docx)

Additional forms (citi_sternberga.pdf)

Additional forms (citi_dryel.pdf)

individualized Comparative Effectiveness of

Models Optimizing Patient Safety and Resident Education

(iCOMPARE)

Protocol

Version 1.4

22 December 2015

Administrative information

Website: http://www.jhcct.org/icompare/default.asp

Email: icompare@jhcct.org

Clinicaltrials.gov registration: NCT02274818

Coordinating Centers:

Clinical Coordinating Center (CCC)
Blockley Hall 1317
423 Guardian Drive
Philadelphia, PA 19104
215-746-2705

Data Coordinating Center (DCC)
Johns Hopkins University
Bloomberg School of Public Health
415 North Washington Street, 2nd floor
Baltimore, Maryland 21231
(443) 287-3170
(443) 287-5797 (fax)

Funding received from:
Accreditation Council for Graduate Medical Education (ACGME)
515 North State Street
Suite 2000
Chicago, Illinois 60654
(312) 755-5000
(312) 755-7498
www.acgme.org

National Heart, Lung, and Blood Institute (NHLBI)
National Institutes of Health
NHLBI Office of the Director
Building 31, Room 5A52
31 Center Drive MSC 2486
Bethesda, Maryland 20892
(301) 592-8573
http://www.nhlbi.nih.gov/

Document distribution

Version	Version date	Distribution	Distribution date
1.1	July 15, 2015	NHLBI	July 16, 2015
1.2	September 24, 2015	NHLBI	September 25, 2015
1.3	October 6, 2015	DSMB, NHLBI	October 6, 2015
1.4	December 22, 2015	DSMB, NHLBI	December 22, 2015

Document history

Version 1.2 – implements additional details on substudy protocols, adverse event reporting, corrections to references, miscellaneous typo corrections.

Version 1.3 – addresses queries raised by NHLBI during their review of Version 1.2 (funding, clarification of length of stay measures, and further detailing of substudy plans).

Version 1.4 – addresses queries raised by DSMB during their review of Version 1.3 and corrects miscellaneous typographical errors.

Contents

Administrative information i

Document distribution ii

Document history iii

Contents iv

Abstract 1

1. Background and rationale 2
2. Aims and hypotheses 4
3. Organization, staffing and administration 6
4. Trial design overview 8
5. Study population 9
   - 5.1. Overview 9
   - 5.2. Internal Medicine training programs 9
   - 5.3. Patients 9
   - 5.4. Program directors, faculty and trainees 10
   - 5.5. Time and Motion Substudy 10
   - 5.6. Sleep and Alertness Substudy 11
6. Recruitment 12
   - 6.1. Recruitment of programs 12
   - 6.2. Recruitment of faculty and trainees 12
   - 6.3. Recruitment of Time and Motion Substudy programs and interns 12
   - 6.4. Recruitment of Sleep and Alertness Substudy programs and interns 13
   - 6.5. Recruitment of patients 13
7. Interventions 14
8. Randomization 15
9. Data collection 16
   - 9.1. Overview and timeline 16
   - 9.2. Patient safety and costs 16
   - 9.3. Trainee education 17
     - 9.3.1. Program director end of year surveys 17
     - 9.3.2. Trainee end of year surveys 17
     - 9.3.3. Trainee just in time surveys 17

9.3.4. Data provided by ACP ... 17
9.3.5. Data provided by ACGME ... 18
9.3.6. Data provided by APDIM ... 18
9.4. Time and Motion Substudy data ... 18
9.5. Sleep and Alertness Substudy data ... 18
10. Data management ... 20
10.1. Overview ... 20
10.2. Patient safety and costs ... 20
10.3. End of year and just in time surveys ... 21
10.4. Data provided by ACP, ACGME, and APDIM ... 21
10.5. Time and Motion Substudy data ... 21
10.6. Sleep and Alertness Substudy data ... 22
11. Biostatistical considerations ... 23
11.1. Sample size and power ... 23
11.2. Data analysis ... 24
11.2.1. Overview ... 24
11.2.2. Patient safety and costs ... 24
11.2.2.1.1. Patient safety hypothesis H1a - 30-day mortality ... 24
11.2.2.1.2. Other safety hypotheses (H1b-H1e) ... 25
11.2.3. Trainee education hypotheses (H2a-H2d) ... 25
11.2.4. Intern sleep and alertness hypotheses (H3a-H3b) ... 26
12. Data monitoring ... 28
13. Ethics ... 29
13.1. Research ethics approval ... 29
13.2. Consent ... 29
13.2.1. Randomization ... 29
13.2.2. Consent for use of data provided by ACGME, APDIM and ACP ... 29
13.2.3. Consent for use of patient data ... 29
13.2.4. Consent for end of year and just in time survey data ... 30
13.2.5. Consent for Time and Motion Substudy ... 30
13.2.6. Consent for Sleep and Alertness Substudy ... 30
13.3. Protections against risk ... 31
13.3.1. Overview ... 31

13.3.2. Patient safety and costs 31

13.3.3. Trainee education 31

13.3.4. Intern sleep and alertness 31

13.4. Confidentiality 32

14. Dissemination and data sharing 33

14.1. Data sharing 33

14.2. Dissemination of study results 33

15. Tables 34

15.1. Trial organization 34

15.2. Committees and centers 35

15.3. Design synopsis 36

15.4. Derivation of the study population (CONSORT diagram) 40

15.5. ICD-9 codes for qualifying principal diagnosis on hospital admission 41

15.6. Education and process outcomes 46

15.7. Data collection schedule 47

15.8. Data collection by when raw data accrue and when raw data are collected, requested, received by iCOMPARE 49

16. References 50

Abstract

In the United States and other countries, policy limiting duty hours in graduate medical education has undergone significant revision in the last decade and become a central point of debate. Evidence from human chronobiology and sleep argues for shorter shifts because fatigue leads to errors. However, evidence from operations research argues for more continuity because patient handoffs also lead to errors and may reduce the effectiveness of education necessary to produce independent clinicians. The evidence from both fields is compelling, resulting in uncertainty regarding how to best configure duty hour standards for fatigue management, high quality patient care, and trainee education. In 2011, the Accreditation Council for Graduate Medical Education (ACGME) imposed more restrictive duty hour standards for all trainees. The new duty hours added that post-graduate year 1 (PGY1) trainees (interns) work no more than 16h of duty periods in a day. This change greatly increased the frequency of patient handoffs. As a result, alternative work schedules have been proposed that combine longer shifts to maintain continuity of patient care with efforts to manage fatigue.

The iCOMPARE trial is a cluster randomized trial of at least 58 Internal Medicine (IM) training programs to compare the current duty hour standards ("**Curr**" throughout this document) with a more flexible schedule ("**Flex**") that is grounded in contemporary understanding of sleep and patient safety and defined by three rules, each averaged over 4 weeks:

1. Work no more than 80 hours per week;
2. Call no more frequent than every 3rd night;
3. 1 day off in 7.

Our primary hypothesis addresses patient safety:

1. *30-day patient mortality under* **Flex** *will not exceed (will not be inferior to) mortality under* **Curr**.

Our secondary hypotheses address education and sleep and fatigue:

2. *Interns in* **Flex** *will spend greater time in direct patient care and education compared to interns in* **Curr**;
3. *Average daily sleep obtained by interns in* **Flex** *will not be less than (will not be inferior to) that of interns in* **Curr**.

iCOMPARE (individualized Comparative Effectiveness of Models Optimizing Patient Safety and Resident Education) will provide the rigorous comparative effectiveness data essential to setting duty hour policies that optimize quality of care and the competency of our future physicians. Moreover, the same two schedules, **Curr** vs. the novel **Flex** scheme, are being compared in the ongoing FIRST Trial (https://clinicaltrials.gov/ct2/show/NCT02050789) in residents in general surgery. The combination of well-designed separate trials in both primarily procedural and non procedural fields will fill the unmet need for a high-quality, generalizable body of evidence to inform national duty hour policy.

---

1. Background and rationale

A 1971 study [1] that found fatigued interns tended to misinterpret electrocardiograms prompted discussion on duty hours, but no action. The well-publicized death of Libby Zion [2] prompted the first state-level regulation of duty hours in 1989 in New York. Under increasing public and legislative pressure to restrict duty hours for graduate medical trainees, the Accreditation Council for Graduate Medical Education (ACGME) implemented duty hour standards for all accredited training programs effective July 1, 2003 [3]. These standards represented one of the largest national efforts ever undertaken to reduce errors in teaching hospitals. The intent of these standards was to improve patient safety; however, the preponderance of data after their implementation demonstrated no definite benefit in safety, concerns for increased risks [4-10], and no clinically important improvements in Internal Medicine Board scores subsequent to the 2003 reform [11]. Subsequently, and in response to a Congressional request, an Institute of Medicine (IOM) committee was charged with making recommendations to optimize resident work hours to improve patient safety. In 2009, the IOM published its report recommending naps for any trainee working over 16h [12]. The ACGME then revised the national standards in 2011 mandating rest periods between duty periods, increased supervision for junior trainees, and a 16h limit on continuous duty hours for interns [13]. However, since the 2011 standards have been implemented, concerns have been raised regarding their impact on patient safety, trainee education, and health care costs. Studies have associated the new standards with less direct patient contact, increased medical errors, increased transitions of care, decreased educational opportunities, and only modestly increased sleep [14-16]. Furthermore, significant dissatisfaction has been reported by program directors and trainees about the negative impact on patient safety and quality of training [17-19].

One of the reasons the ACGME limited continuous PGY1 work to 16h was to increase sleep time and thereby prevent fatigue-related errors. However, limiting work hours to increase sleep time does not appear to have been effective. Aggregate Actiwatch® + sleep diary data from 301 IM interns contributing >8,000 days reveal that their mean daily total sleep time is comparable across all duty-hour schedules that we have investigated prior to and following the 2011 limit of 16h [Dinges DF and Basner M, unpublished data]. In agreement with this conclusion are data from single center randomized clinical trials in internal medicine that also suggest some alternative work-hour models may be equal or superior in relevant patient and trainee outcomes [20, 21]. In one study that randomized IM interns to a schedule with 16h limits or 30h limits [15], during the window on which interns were on their longest shifts (a 48h period comprising either the 16h shift or the 30h shift), interns on the 16h schedule slept approximately 3 hours more than interns on the 30-hour schedule. However during a 4-week clinical rotation, interns on the 16h schedule did not sleep significantly more on average than interns on the 30h schedule. Additionally, transitions in care were 130-200% higher in the 16h schedule. These data make a compelling case that the current policies might be improved to meet the complex and competing needs of the public and medical communities.

In 2013, researchers at the University of Pennsylvania School of Medicine and Harvard Medical School began developing the protocol for a 2-year crossover trial of a 28-hour duty hour regimen including a protected sleep period of 4 hours versus the current duty hour regimen and assembling a study team to prepare an application for funding to the National Heart, Lung, and Blood Institute (NHLBI). Application to the ACGME for a waiver from current duty hour standards for programs participating in the proposed trial and for funding to support the work of preparing the application was also initiated and ultimately approved. With expansion of the research team to include investigators at the Johns Hopkins University Bloomberg School of Public Health, an R01 grant application for the trial was submitted to NHLBI in February 2014. This funding application was not successful. After discussion and regrouping, the iCOMPARE investigators decided to request funding from the ACGME for a 1-year trial protocol that focused on the patient safety (mortality) hypothesis and compared the same duty hour standards being

compared in the FIRST Trial, namely, flexible standards with 3 governing rules versus the current standards. The ACGME approved this revised protocol in September 2014 and agreed to fund the patient safety aim. Work to recruit and randomize IM programs to be ready to implement the flexible duty hour standards versus current standards in academic year 2015-2016 was initiated in fall 2014. In November 2014, the iCOMPARE investigators submitted a revision to their unfunded R01 application to NHLBI, requesting funding to support data collection and analysis to evaluate additional patient safety hypotheses and education and sleep and alertness hypotheses, within the trial funded by the ACGME. The additional data collection and analysis tasks include additional analyses of Medicare claims data, additional surveys of trainees regarding training and education experiences, and two substudies, "Time and Motion" and "Sleep and Alertness", each to be conducted at a subset of the participating IM programs. This revised application was approved for funding by the NHLBI in July 2015. The protocol described herein is the ongoing ACGME protocol expanded to include the additional aims and hypotheses approved and funded by the NHLBI in July 2015.

---

2. Aims and hypotheses

Since 2003, resident physician duty hours have been regulated across the US in the interest of reducing resident fatigue and promoting patient safety. Continuous duty hours for first year trainees (interns) were restricted further in 2011. However, recent studies have associated the 2011 standards with less direct patient contact, increased medical errors, increased transitions of care, decreased educational opportunities, and only modestly increased sleep [14-16]. Program directors and trainees have expressed significant concern about the negative impact they perceive these rules have on patient safety and quality of training [17-19]. And so it seems that what was intended as a way to reduce error by managing resident fatigue is now felt by many to promote error through the compression of schedules and increased handoffs as well as decreased educational opportunities and professionalization required to produce independent physicians. No existing research helps navigate resident duty hour policy between these competing considerations. The goal of the iCOMPARE study is to fill these gaps. We will randomize internal medicine training programs to one of two duty hour schedules: the current standard (**Curr**ent; **Curr**) or a flexible schedule (**Flex**ible**; Flex**) and complete the following specific aims:

Specific Aim 1: Examine patient safety and costs under **Curr** and **Flex** duty hour schedules.
Specific Aim 2: Examine the quality of education under **Curr** and **Flex** duty hour schedules.
Specific Aim 3: Examine intern sleep time and alertness under **Curr** and **Flex** duty hour schedules.

iCOMPARE has one primary hypothesis:

H1a: 30-day patient mortality under **Flex** will not exceed (will not be inferior to) mortality under **Curr**.

iCOMPARE will test related and complementary secondary hypotheses regarding:

Patient safety and costs:

H1b: 7-day and 30-day hospital readmission rates under **Flex** will not exceed (will not be inferior to) the rates under **Curr**.

H1c: Complication rates defined by selected AHRQ Patient Safety Indicators (http://www.qualityindicators.ahrq.gov/modules/psi_resources.aspx) under **Flex** will not exceed (will not be inferior to) complication rates under **Curr**.

H1d: The rate of prolonged length of stay, defined as a stay that exceeds the Hollander-Proschan prolongation point by one day [7, 22, 23], under **Flex** will not exceed (will not be inferior to) the rate of prolonged length of stay under **Curr**. The Hollander-Proschan prolongation point is a statistic calculated for a given condition that identifies the day during the hospital stay when the discharge rate begins to decline.

H1e: Overall costs, as indicated by total Medicare payments, under **Flex** will not exceed (will not be inferior to) overall costs under **Curr**.

Trainee education:

H2a: Interns in **Flex** will spend greater time in direct patient care and education compared to interns in **Curr**.

H2b: Trainees in **Flex** will report greater satisfaction with their educational experience (greater ownership, greater continuity and lower burnout) than trainees in **Curr**.

H2c: Faculty in **Flex** will report greater satisfaction with their clinical teaching experiences and greater perceptions of safety, teamwork and supervision than faculty in **Curr**.

H2d: Standardized test scores for interns in **Flex** will not be less than (inferior to) those for interns in **Curr**.

and Intern sleep and alertness:

H3a: Average daily sleep obtained by interns in **Flex** will not be less than (will not be inferior to) that of interns in **Curr**, as determined by a 14-day period of sleep monitoring using actigraphy and daily sleep diaries.

H3b: Interns in **Flex** will not have (will not be inferior to) greater average subjective sleepiness via Karolinska Sleepiness Score (KSS) [24], or lower average behavioral alertness via psychomotor vigilance test (PVT) [25] than interns in **Curr**, as determined by a 14-day period of morning sleepiness-alertness monitoring.

The iCOMPARE primary outcome (30-day mortality) was chosen to ensure that any policy change in resident duty hours will not result in inferior patient safety. However, additional patient safety measures, as well as costs, education and fatigue management, are critically important considerations which our study addresses. The results of iCOMPARE will help the ACGME in its ongoing deliberations about optimal resident duty hour schedules. Changes in ACGME policies affect every teaching hospital in the United States, and as a consequence, every patient.

---

3. Organization, staffing and administration

The iCOMPARE investigators are organized into two distinct but collaborating centers, the Clinical Coordinating Center (CCC), at the University of Pennsylvania, and the Data Coordinating Center (DCC), at the Johns Hopkins Bloomberg School of Public Health. Each of these centers has separate areas of responsibility; both will work together to achieve the aims of the project. The CCC will have primary responsibility to manage and implement the protocol; to recruit, train and manage the participating programs; to oversee the timely collection of relevant study data; to ensure compliance with IRB and other regulatory bodies; and to distribute supplies and funds as appropriate. The DCC will have primary responsibility to receive and manage all study data files; to maintain a project website and facilitate project communications; to prepare interim and final reports of the study's progress and results; and to perform statistical analyses of the study data. The Centers will work together to establish and maintain quality assurance in the participating residency programs and to provide timely high-quality publications of the study's results.

The CCC and the DCC will share responsibility for oversight and management of the participating residency programs. The CCC will coordinate protocol implementation at each program, will review intern duty schedules to ascertain compliance with the appropriate iCOMPARE intervention arm, and will develop and administer surveys. The DCC will create and manage an internet-based data management system for the receipt of survey and other data collected from trainees and program directors, and for later merging those data with data from other sources (such as CMS claims data and ACGME survey data). The CCC will run periodic conference calls with the program directors and separate periodic calls with site coordinators involved in the sleep and time and motion evaluations. The CCC and DCC will together establish systems for monitoring protocol implementation and site performance, and for determining the composition and frequency of any "for cause" site visits.

Table 15.1 displays the organizational structure of the team conducting the trial. The primary leadership body for the trial is the Steering Committee, composed of key investigators from both the CCC and the DCC. A smaller Executive Committee, appointed by the Steering Committee, facilitates decision making.

The Steering Committee (SC) is the principal decision-making body for iCOMPARE and is chaired by David Asch, the Principal Investigator of the CCC; James Tonascia, the Principal Investigator of the DCC serves as vice-chair. Eleven other investigators from the CCC and DCC and the NHLBI Project Officer comprise the members at large. The SC is responsible for approval of the trial protocol and any subsequent amendments and for votes on other important decisions. A quorum of the SC will be seven members, with decisions made by agreement of a majority of those participating. It is expected that the SC will appoint sub-committees, possibly to include non-members of the SC, to make recommendations in areas such as protocol implementation issues, publications, and ancillary studies. The SC will meet monthly by teleconference or in-person.

The Executive Committee (EC) will manage day-to-day issues in iCOMPARE and will make decisions between SC meetings. The EC will organize and prepare agendas for the SC meetings. Sanjay Desai serves as chair of the EC; Judy Shea serves as EC vice-chair. The five members at large are a subgroup of the SC membership. The EC will meet weekly by teleconference, although the frequency of meetings may vary depending on circumstances.

The ACGME is supporting iCOMPARE by providing a waiver from currently mandated duty hour standards for IM programs randomized to the **Flex** arm in iCOMPARE and by providing funding to

support work related to aims H1a, H2b, H2c, and H2d. The ACGME does not participate in iCOMPARE conduct, data analysis, nor preparation of publications.

The National Heart, Lung, and Blood Institute (NHLBI) funding is supporting work related to aims H2a, H3a, and H3b as well as the work of the Data and Safety Monitoring Board (DSMB). The DSMB will be appointed by and advisory to NHLBI. The DSMB will approve the protocols for the Time and Motion and Sleep and Alertness Substudies and will monitor the trial conduct. The trial will produce no interim patient outcome data on which to judge its safety until after the intervention has concluded, but the DSMB may monitor accumulating performance data and may monitor reports of safety issues experienced by trainees. The data monitoring is described in Section 12.

An Advisory Board (AB) has been appointed by the SC to make regular recommendations about the design and conduct of the project. The AB is chaired by Lisa Bellini, MD, a SC member at large. The remaining AB members will not otherwise be part of the study team and will include leaders in graduate medical education, and policy. The AB will make its reports directly to the SC.

Program Directors from participating Internal Medicine residency programs represent site leaders for this multicenter trial. The CCC will host conference calls for all participating program directors during the intervention period. Given the contributions required by participating Program Directors, efforts will be made to acknowledge them appropriately in publications as authors or other contributors as consistent with conventions and contributions.

The Research Group for iCOMPARE consists of investigators and staff from the CCC and the DCC, members of the Advisory Board, the Program Directors of the participating training programs, site coordinators based at the programs, and faculty and trainees participating in the project.

Table 15.2 summarizes the role and membership of the trial committees and centers.

---

4. Trial design overview

The iCOMPARE study design is summarized in Table 15.3. iCOMPARE will use a one-year randomized cluster randomized design to compare two alternative work schedules for interns in at least 58 IM programs. The control schedule (**Curr**) reflects current duty hour standards. The intervention schedule (**Flex**) has three conditions, each averaged over four weeks: (1) work no more than 80 hours per week; (2) call no more frequent than every third night; (3) one day off in seven. The ACGME has agreed to waive duty hour standards for participating programs randomized to **Flex**. The interventions are described in more detail in Section 7. We will evaluate the differences between these alternative duty hour regimens in terms of patient safety and costs, trainee education, and trainee sleep and alertness.

The interventions will be administered in parallel with a target allocation ratio of 1:1. iCOMPARE is designed to be a pragmatic trial. We selected a flexible set of rules for the intervention in response to input from the community of internal medicine residency directors. Our intervention arm is relevant for all PGY levels. The test of the primary hypothesis (patient safety) will be a non-inferiority test. The trial is designed to have at least 80% power to detect a difference in one year change (trial year – pretrial year) of 1% in 30-day mortality with 5% type I error.

The trial includes a main protocol in which all randomized IM programs participate and two substudies, “Time and Motion” and “Sleep and Alertness”, each conducted at a subset of IM programs and focusing on more detailed data collection at the intern level. The Time and Motion Substudy addresses hypothesis H2a in detail. The Sleep and Alertness Substudy addresses hypotheses H3a and H3b in detail.

5. Study population

5.1. Overview

In terms of randomization unit, the iCOMPARE study population is comprised of Internal Medicine training programs. In terms of entities providing data used to address the iCOMPARE hypotheses, iCOMPARE has 4 study subpopulations: the program directors leading the IM programs randomized to duty hour regimen, the faculty teaching at those programs, the trainees at those programs, and the patients cared for by faculty and trainees of these programs. iCOMPARE will obtain data both directly and indirectly from program directors, program faculty, and trainees (e.g., directly by survey or observation by iCOMPARE staff and indirectly by ACGME survey data shared with iCOMPARE) and indirectly from patients (e.g., patient data will be obtained from Medicare claims records).

5.2. Internal Medicine training programs

Because the outcomes for the patient safety and cost aims will be determined using Medicare data, the IM programs participating in iCOMPARE and experiencing the study duty hour standards must meet criteria relevant to Medicare patient volume. Because the treatment is applied at the IM program level, we need sufficient trainee presence in the care of these patients and hence participating programs must meet criteria related to program size. Table 15.4 displays a CONSORT style diagram of derivation of the iCOMPARE population of IM training programs. There are 379 IM training programs in the country. We applied the following eligibility criteria to identify programs that would be invited to apply:

1. At least one hospital with resident to bed ratio > 0.105 (excluded bottom 50% of hospitals by resident to bed ratio)
2. Sufficient Medicare patient volume (excluded bottom 25% of hospitals by patient volume)
3. In upper 75% of programs by program size

119 programs reflecting the bottom 50% in resident-to-bed ratio and the bottom 25% in patient volume related to the diagnoses in which mortality will be measured were excluded. Within the 260 programs that remained, the 65 in the lowest quartile of program size were excluded to ensure feasibility of obtaining sufficient trainee measurements. 195 remaining programs were eligible for recruitment to participate in iCOMPARE. Recruitment is discussed in Section 6. These 195 programs averaged about 30 interns, 25 PGY2 trainees, 25 PGY3 trainees, and 10 faculty per program.

5.3. Patients

Our study population for evaluation of patient safety and costs will be Medicare fee for service (FFS) beneficiaries at least age 65.5 years at hospital admission and admitted to one of the acute care hospitals affiliated with the randomized IM programs and at which the IM program implements the randomly assigned duty hour schedule between July 1, 2015 and June 30, 2016 and with any of the eligible principal diagnoses (see Table 15.5). These diagnoses apply to the majority of patients on a typical medical service and account for most of the deaths and other safety indicators. [26] While patients who are not Medicare beneficiaries are also cared for by trainees, 67.3% of patients on the medical services are Medicare beneficiaries and 72.2% of the mortality in medical admissions is in Medicare beneficiaries.[27, 28] The analyses are limited to FFS beneficiaries because CMS claims data are available for Medicare FFS patients only. All FFS Medicare patients will have complete, linked data: inpatient (Medicare Part A), outpatient, physician (Medicare Part B), and associated denominator files. Patients will be included if they were not enrolled in a managed care program six months before admission and one month post

discharge. The minimum age is 65.5 years and FFS status in the 6 months prior to admission is required so that claims are available for 6 months prior to the qualifying admission. The diagnoses were chosen to reflect the vast majority of patients on the typical IM service. Examining all patients, rather than just Medicare aged FFS patients, would be ideal but is not feasible because 30-day mortality, as well as the secondary outcomes, require 'linkable' data to events occurring outside the hospital, something not practical to obtain outside the Medicare system (i.e., it would be impractical to consent patients to be able to see their data, or to rely on numerous insurance companies or various state databases due to the scale of this trial). No patients will be excluded based on gender or race/ethnicity. We expect very little change in year to year demographic and clinical characteristics of the patients admitted to each hospital, as we observed in previous studies [9, 29].

We will address the change in ICD codes as follows. We will use the official crosswalk between the two coding systems and ensure that the principal diagnosis codes for the index admissions include all the possible codes identified in the crosswalk in both systems. Secondly we will have a separate dataset in which we will experiment and test the validity of the crosswalk, which is not part of the safety outcomes and cost analysis. This dataset will serve as the lab for the investigators to define the medical admissions in the ICD10 system in a way that is consistent with the ICD9. The lab data set will comprise patients admitted to hospitals affiliated with the remaining 137 eligible IM programs that did not elect to participate in iCOMPARE. This way we ensure that our experimental data are very similar to the data obtained for the programs participating in iCOMPARE. After we are satisfied with the validity of the crosswalk, we apply it to claims at hospitals affiliated with the randomized programs. It is helpful that the list of possible complications in medical admissions is limited.

5.4. Program directors, faculty and trainees

The directors, faculty and trainees affiliated with the participating IM programs will provide data to address iCOMPARE aims. All of these individuals are adult and there is no selection for gender or race/ethnicity. Depending on the aim being addressed, the iCOMPARE data collection focus may be all program directors, all program faculty, all trainees, all interns, or subsets of any of these or combinations of any of these.

5.5. Time and Motion Substudy

Six of the participating IM programs will be recruited to participate in the Time and Motion Substudy; the program director will consent to their program's participation in the Time and Motion Substudy. We will describe the substudy to all program directors and develop a list of those interested in participation. Within that list, in order to maximize efficiency, we will prioritize **Flex** programs with the largest numbers of interns on a **Flex** schedule per each rotation/block. Once the three **Flex** programs are identified, we will select three **Curr** programs from that list; the **Curr** programs must approximate key **Flex** program characteristics in terms of size, type of program, geography of program, and similarity of rotations. At each **Flex** site, we will randomly select 10 interns who are on **Flex** rotations for recruitment; we will continue to randomly select interns until 10 interns have consented to observations that will occur over a 2-4 week period. At the **Flex** sites we will recruit interns who are on **Flex** rotations. At the **Curr** sites we will identify the interns on rotations comparable to the **Flex** rotations and we will randomly select interns for recruitment from that pool. The only criteria for interns to be recruited into the substudy at a **Flex** IM program are being on a **Flex** rotation and consenting to participation (i.e., consenting to observation). The only criteria for interns to be recruited into the substudy at a **Curr** IM program are being on a rotation similar to a rotation observed at a **Flex** program and consenting to participation (i.e., consenting to observation).

5.6. Sleep and Alertness Substudy

From the IM programs that agreed to participate in the main protocol and that have already been randomized to **Curr** or **Flex**, we will identify comparable programs. These comparable programs will be asked to agree to also participate in the Sleep and Alertness Substudy. We plan to recruit 384 interns from participating IM training programs (50% randomized to **Flex**, 50% randomized to **Curr**) for the 14-day sleep and alertness evaluations (see section 6.4).

6. Recruitment

6.1. Recruitment of programs

The target for program enrollment in iCOMPARE was 58 IM programs in fall 2014. This timing allowed program staff to discuss the potential for iCOMPARE participation with prospective academic year 2015-2016 interns while they were interviewing for residency appointments and also sufficient time to prepare schedules meeting the **Flex** criteria if assigned to **Flex**. Starting in April 2014, CCC leadership began publishing the plan for iCOMPARE and soliciting program directors for interest and input. A presentation was made at the spring 2014 APDIM meeting and a summary description of iCOMPARE procedures and requirements was provided to interested program directors. Interested program directors could sign up for additional information by registering with the iCOMPARE website.

In summer 2014, the DCC and CCC sent application forms to interested program directors. The application form requested information regarding the hospitals at which the program planned to implement the iCOMPARE assigned duty hour schedule and acknowledgement of the responsibilities of participation. To confirm consent to participate at the program level, each program director was required to provide written institutional agreement to participate signed by the designated institutional official. Care was taken with the randomization of programs that operated in the same hospital to ensure that either the hospital would be active in iCOMPARE under only one of the programs or that the programs were randomized as a cluster. Based on program size data derived from the ACGME year book, these programs are estimated to have a mean of 30 interns and a mean of 50 PGY 2-3 trainees (residents).

Programs meeting randomization criteria were randomized to duty hour regimen starting in November 2014. Randomization ended in April 2015 with 63 IM programs randomized.

6.2. Recruitment of faculty and trainees

The recruitment process for IM programs involved the consent of the program director and the institution associated with the program, but did not involve consent of program faculty nor trainees in PGY2 or PGY3 years nor the incoming interns. While the individuals comprising these groups have no choice about their program's iCOMPARE participation, each of these individuals may opt in or out of individual participation in each iCOMPARE survey and in or out of substudy participation. Each iCOMPARE survey is prefaced with a statement that participation is voluntary and consent for the survey is assumed if the survey is completed. Recruitment of faculty and trainees in the main iCOMPARE protocol thus becomes an effort to solicit completion of surveys. Strategies to be employed to maximize participation in surveys include exhortatory emails from program directors, iCOMPARE leaders and others influential groups, and lottery type awards of token incentives (e.g., $20 gift cards).

6.3. Recruitment of Time and Motion Substudy programs and interns

We will describe the substudy to all program directors and develop a list of those interested in participation. The program director will consent to his/her program's participation in the substudy.

Recruitment of interns for the substudy will be completed centrally so that program directors are not involved in consent of interns who are training under them. Program directors will be asked to provide

information to interns by way of presentations and to encourage participation but will not be part of the consent process and will not be privy to the electronic sign up sheet by which interns will initiate their individual recruitment for the substudy. Interns at the participating programs will also be recruited by email solicitation by iCOMPARE leaders. Interns will be asked to indicate interest and initiate the consent process by providing their contact information through an electronic application on the iCOMPARE website. Interns providing contact information will be sent the consent by a central substudy staff member; the central substudy staff member will review the consent with the intern during a telephone conversation. The intern will be asked to sign the consent statement electronically once all his/her questions have been answered. The intern will be provided with a copy of the signed consent statement. Interns who complete the Time and Motion Substudy data collection will receive a $50 giftcard.

6.4. Recruitment of Sleep and Alertness Substudy programs and interns

From the IM programs that agreed to participate in the main protocol and that have already been randomized to **Curr** or **Flex**, we will identify comparable programs (50% randomized to **Flex**, 50% randomized to **Curr**). These comparable programs will be asked to agree to also participate in the Sleep and Alertness Substudy. From those programs agreeing to participate in the Sleep and Alertness Substudy, we will recruit a sample size of 384 interns (see power calculations) for the 14-day sleep and alertness evaluations.

Site coordinators and program directors will facilitate the interns' participation in the Sleep and Alertness Substudy, but they will be instructed not to influence whether an intern elects to participate or not participate in the Sleep and Alertness Substudy. Site coordinators will provide interns with an information package that includes the following items: (a) an information flyer that briefly summarizes the study, (b) the informed consent form (together with information on how to contact the study team with any questions), (c) a gift card worth up to $140 ($10/day for each day of completed Smartphone and acitgraphy data), and (d) a pre-paid return envelope for mailing the consent form and the brief survey (alternatively, interns can hand the consent form and the brief survey to the site coordinator for mailing to the study team). Only site coordinators and the study team will know which interns consented to participate in the study. However due to the fact that interns have to wear actiwatches continuously during one 14-day period, they can be identified as study participants during this period (this is explicitly mentioned in the informed consent form). After written informed consent is received by the study team, the intern will be scheduled for a 14-day data collection period. In the week prior to this collection period, the study team will mail an actigraph, a Smartphone, and a copy of the informed consent form signed by both the intern and the study principal investigator to the site coordinator, who will hand them to the intern before the start of the data collection period. After the 14-day collection period, the intern will either return the equipment to the site coordinator who will mail it back to the study team or return it themselves in the prepaid envelope.

6.5. Recruitment of patients

Since patient data will be obtained exclusively through purchase of Medicare claims files from ResDAC, individual patients will not be contacted by iCOMPARE for consent nor for data collection – i.e., patients are not recruited for iCOMPARE participation.

---

7. Interventions

Participating IM programs will be assigned to one of the following groups:

1. The control schedule (**Curr**) reflects current duty hour standards established by ACGME.
2. The intervention schedule (**Flex**) has three conditions, each averaged over 4 weeks:

   (1) work no more than 80 hours per week;
   (2) call no more frequent than every third night;
   (3) one day off in seven.

The ACGME has agreed to grant programs randomized to the **Flex** arm waivers from the current duty hour standards. The waiver applies to programs that meet the ACGME's standards for accreditation. While only IM programs in good standing with the ACGME could be randomized, there is potential for a program to lose that standing at any time during the conduct of the trial. Any **Flex** program that loses ACGME accreditation must revert to the **Curr** duty hour schedule as of the loss of accreditation, regardless of iCOMPARE participation or timeline.

The intervention period begins in July 2015 and ends in June 2016. While **Flex** programs are encouraged to use their **Flex** schedule on all rotations, each program has discretion to choose the rotations to which the **Flex** intervention will be applied. The intervention can be used on selected rotations (e.g., ICU only) instead of all rotations through which IM trainees cycle. All trainees rotating on services in the participating IM program are permitted to follow the duty hour rules assigned to the IM program by iCOMPARE. This includes rotators from other (non iCOMPARE) IM programs, as well as rotators from other departments, e.g. emergency medicine.

Program directors, faculty and trainees cannot be masked to intervention group. While there is no prohibition against discussion of iCOMPARE with patients, discussions are unlikely. Patients are likely to be masked to intervention group.

8. Randomization

The DCC generated the random treatment assignment schedule using SAS version 9.3. The randomization schedule was designed to yield an expected assignment ratio of 1:1 for **Curr** and **Flex** and employed a permuted block design, with block sizes documented at the DCC. Documentation of all these processes are retained at the DCC and are accessible only to authorized personnel. Adjustment for residual or other imbalances in the baseline composition of **Curr** and **Flex** groups, if needed, will be done using multiple regression techniques at the time of data analysis rather than through stratification in the design.

IM program eligibility was confirmed by the CCC, including receipt of institutional agreement to participate signed by the designated institutional official. After confirmation of eligibility, each IM program's ID was irrevocably linked to the next ordered treatment assignment using a program accessible to DCC personnel. If more than one program was to be randomized in a session, the set of programs to be randomized in the session was put in random order by a DCC staff member who was not the DCC staff member generating each program's treatment assignment. The data system automatically stored the date and time of assignment, the identity of the DCC staff person making the assignment, the program's ID, and the treatment assignment. Eligible programs that share a hospital were randomized together (i.e., to the same treatment group) because some residents will be rotating through both hospitals.

Treatment assignments were e-mailed to program directors at participating IM programs and posted on the iCOMPARE website.

9. Data collection

9.1. Overview and timeline

iCOMPARE is collecting data to address its 3 specific aims: examination of patient safety and costs, examination of the quality of trainee education, and examination of intern sleep time and alertness. Data on patient outcomes and costs of health care will come from Medicare. Data collected directly from trainees and program directors by iCOMPARE will be supplemented with data collected on trainees, program directors and faculty by national organizations such as the ACGME, the American College of Physicians (ACP) and the Association of Program Directors in Internal Medicine (APDIM). As described below, the time period of active data collection by iCOMPARE survey or observation will be May 2015 through June 2016.

9.2. Patient safety and costs

The patient safety and cost data that will be used to address hypotheses H1a through H1e will be obtained from Medicare claims records. These records will be obtained through application to and purchase from the Research Data Assistance Center located at the University of Minnesota School of Public Health (ResDAC; http://www.resdac.org/). All requests for Medicare data proceed through ResDAC. We will obtain claims data from CMS for calendar years 2013 through 2016 and will construct three analysis cohorts, each including patients with a qualifying admission diagnosis: Baseline 1 (admission between 7/1/2013-6/30/2014), Baseline 2 (admission between 7/1/2014-6/30/2015), and Trial year (admission between 7/1/2015-6/30/2016). For each patient in each analysis cohort, we will obtain their encounters with the medical system for at least 6 months before and 6 months after the qualifying admission; hence the minimum age at qualifying admission is 65.5 years. Medicare data for the previous calendar year (Jan-Dec) are first made available by CMS to researchers each year around October. Data needed for creation of each analysis cohort of patients are shown per the table. We will request the following file types: MEDPAR (for inpatient encounters), Carrier for physician bills, Outpatient file (includes ED visits), Durable medical equipment, Hospice care, and Home care.

**Analysis cohort (range of possible dates of qualifying admission)**	**Calendar year of CMS data of interest (earliest date available)**
Baseline 1 (7/1/2013 - 6/30/2014)	2013 (Fall 2014)<br>2014 (Fall 2015)
Baseline 2 (7/1/2014 - 6/30/2015)	2014 (Fall 2015)<br>2015 (Fall 2016)
Trial year (7/1/2015 - 6/30/2016)	2015 (Fall 2016)<br>2016 (Fall 2017)

Use of 2 baseline periods provides for more stable estimates of the safety outcome measures and costs, especially for the low rate measures, such as 30-day mortality. Having more stable estimates helps with the power calculations to detect differences between **Flex** and **Curr**. The claims for Baseline 1 will be finalized in April 2016, allowing us to prepare analysis files, define and construct outcomes and develop risk adjustment models, such that when Baseline 2 claims are finalized in April 2017, we are able to apply the code we have developed for constructing and analyzing the data.

9.3. Trainee education

The data that will be used to address hypotheses H2a through H2d will be obtained from surveys completed by program directors and trainees, collected by other groups (ACP, ACGME, and APDIM) and shared with iCOMPARE, and collected under the Time and Motion Substudy.

9.3.1. Program director end of year surveys

Program directors will be surveyed twice, in May 2015 and May 2016. The email requesting completion will include a reminder about iCOMPARE, a brief summary of the type of information about to be queried, a summary of how iCOMPARE will use and share the information, a statement about strategies for data and identity security, a statement about participation implying consent for iCOMPARE to use the data, and a statement that participation is voluntary. The email will also include a link to the data collection website.

The surveys will query program characteristics and perceptions and satisfaction with training and supervision. Since there is only one program director per program, anyone privy to the raw data will be able to identify the respondent. When data are presented, effort will be made to anonymize responses to the extent possible and avoid disclosure of details that may identify a particular program.

9.3.2. Trainee end of year surveys

Trainees will be surveyed twice, in May 2015 and May 2016. The email requesting completion will include a reminder about iCOMPARE, a brief summary of the type of information about to be queried, a summary of how iCOMPARE will use and share the information, a statement about strategies for data and identity security, a statement about participation implying consent for iCOMPARE to use the data, and a statement that participation is voluntary. The email will also include a link to the data collection website. The surveys will query perceptions and satisfaction with work and supervision. The data collection will be such that program is identifiable for a set of responses but not the individual responding.

9.3.3. Trainee just in time surveys

These surveys will be administered throughout the intervention year and will be directed to a random sample of the interns in target IM rotations. The email requesting completion will include a reminder about iCOMPARE, a brief summary of the type of information about to be queried, a summary of how iCOMPARE will use and share the information, a statement about strategies for data and identity security, a statement about participation implying consent for iCOMPARE to use the data, and a statement that participation is voluntary. The email will also include a link to the data collection website. The surveys will query training experiences in the prior 24 hours – e.g., number and types of patient encounters and participation in education activities. The data collection will be such that program is identifiable for a set of responses but not the individual responding.

9.3.4. Data provided by ACP

The ACP has agreed to provide the In-Training Examination (ITE) scores for 2015 and 2016. Most commonly, PGY2 trainees take this exam in the fall of the PGY2 year. We expect 80% of the interns in each year to proceed to the PGY2 year. We estimate that the ITE scores will be provided by the ACP to

iCOMPARE in the winter of each year. The ACP has agreed to share these data, de-identified at the level of the respondent, but identifiable at the level of the program.

9.3.5. Data provided by ACGME

The ACGME has agreed to provide iCOMPARE with portions of the data it collects routinely from trainees and faculty. The ACGME has agreed to provide data related to attitudes and perceptions of training from its year end survey of trainees (interns, PGY2, PGY3) and data related to perceptions of safety, teamwork, supervision, and costs from its year end core faculty survey. The data will be de-identified at the level of the respondent, but identifiable at the program level. Response rate for the trainee survey must exceed 70% for ACGME accreditation. Response rate for the faculty survey is required to be above 60% for ACGME accreditation. Faculty response rates generally exceed 80%.

9.3.6. Data provided by APDIM

The APDIM has agreed to share their survey data regarding perceptions of morale, continuity of care, attendance at conferences, burnout, existing nap opportunities and schedules with iCOMPARE. The data will include program identifiers.

9.4. Time and Motion Substudy data

Observations will occur over a 2-4 week period mid-year (duration depends on availability of observers). Medical students and nursing students on vacation or other nonscheduled blocks will be recruited to be observers. Observers will be trained in the categorization of intern activities and will undergo quality control assessments. Handheld applications (e.g., iTouch) will be used to record time-in-motion assessments. This methodology has been used by our investigators recently in a multi-institutional study [14]. Observers will follow participating interns through a variety of shifts to quantify the amount of time they spend in various activities. Our primary outcome is time spent in direct patient care. Interns will be followed over the duration of their shifts; shifts will be sampled proportionate to the amount of time interns spend in them. Our goal is to observe 2-4 shifts per participating intern, varying the position in the call cycle and sampling both days and nights. Each intern enrolled in the substudy will be assigned a unique identification number known only to the central staff member who consents the intern. The identification number will be used to identify the intern's individual level data.

9.5. Sleep and Alertness Substudy data

After providing informed consent, interns will be asked to wear a wrist actigraph for 14 consecutive days. Such wristwatch-like devices are safe and now widely available and used to remotely monitor sleep-wake patterns of people. Each morning of the 14 days, interns will be asked to complete the following on the Smartphone sometime between 6 AM and 9 AM. Completion of all Smartphone tasks will require no more than 5 minutes each day. The tasks include: answer a few brief questions about the current work shift and the last sleep period; rate their sleepiness and report periods of excessive sleepiness; and complete a reaction-time-based 3-minute Psychomotor Vigilance Test (PVT).[25] If interns have not completed these assessments by 9 AM, the research staff may contact them to remind them. Interns will be compensated with a gift card worth up to $140 ($10/day for each day of completed Smartphone and actiwatch data) that will be activated after completion of the study. Sleep–wake data acquisition will not occur in June or July due to high variation in activities and rotations. Each intern enrolled in the substudy will be assigned a unique identification number known only to the central staff

member who consents the intern. The identification number will be used to identify the intern's individual level data.

---

10. Data management

10.1. Overview

The iCOMPARE data management system is readily accessible, secure, robust and reliable. It accommodates many data types and modes of capture. The data are stored in SQL-style architecture. This allows all necessary data manipulation functions including data linkage on program, treatment group, or other linkage key. It combines server-side and client-side programming, to allow for efficient entry and management of data. We use proven technologies including Microsoft's web server languages (.NET) and database technologies (SQL Server, Jet), standard off-the-shelf browsers (Internet Explorer, Safari, Chrome, and Firefox are supported), and widely used client-side tools including JavaScript and jQuery. We use SSL-encryption for all data transmission. Every data element is tagged with its source (individual user ID or external) and a date-time stamp indicating the date and time of entry or modification. Thus, we have a complete and auditable trail for every data element in the system.

Data are saved on a dedicated server maintained in a guarded, key lock-entry data center with appropriate fire suppression and redundant power. In our experience, server downtime has been near-zero. The server will remain fully patched with updates and will have all unnecessary services, programs, and user accounts deleted or disabled. All portions of the data system website will be password protected using a standard challenge/response system coupled with a user-specific identity system requiring users to log in with their personal PIN and password, which are checked before the login is completed. Once the user is logged in, all activities are stamped with the user's PIN and date-time stamp.

iCOMPARE servers are backed up daily from the web server to dedicated backup devices within the data center. We also separately download study databases three times daily to time-specific files on a separate computer located within Johns Hopkins. These downloads allow us to roll back the system to any previous state within approximately eight hours in the event of a catastrophic failure. These backups are periodically burned to both optical disks and external hard disks for semi-permanent, locked off-site storage. Finally, our web servers are mirrored on dedicated machines within the Johns Hopkins firewall for complete and immediate restoration of website services in the event of a failure. All backups and databases are stored in secure locations and on password-protected computers, and backups are kept offsite from the primary computer systems. Backups are tested to ensure that they are working properly when and if needed.

10.2. Patient safety and costs

The Medicare files will be stored at the Center for Outcomes Research (COR) at The Children's Hospital of Philadelphia (CHOP). COR's user accounts and access-controlled, protected server are managed by senior staff under the supervision of the Director and Associate Director. Each user is assigned a unique user ID and password. Sharing of access credentials is prohibited. Automated mechanisms are in place to enforce password controls, including password length and complexity requirements, minimum/maximum age, re-use limitations, and failed attempt/lockout requirements. Idle timeout features are configured to activate after 15 minutes of inactivity. The server is configured as a Trusted HP-UX server; therefore, all activities for critical systems and services are logged as part of normal maintenance operations and to monitor for unauthorized activities. All applications using the original data files from CMS are run on the offline, private server, thereby eliminating the need to house the original data on desktop or laptop computers and reducing the risk of security breach. Once uploaded

to the COR server, original data are kept in a fire-rated safe within a card-protected storage room within the COR offices, to which only the Director and the Senior Systems Analyst have access.

Access to the server housing the CMS data is provided by encrypted VPN separate from the hospital's main network; COR personnel connect via dedicated PC workstations running X11 servers to the remote server system. Cisco firewalls are utilized for network segregation. In addition, intrusion detection and prevention technologies are deployed throughout the network to identify and protect against malicious code, denial of service attacks, and viruses. A variety of tools and techniques are used to conduct regular internal and external vulnerability scans so that security vulnerabilities can be quickly identified and addressed, in accordance with CHOP policies and regulatory requirements.

All data received from Medicare are Standard Analytic Files that are finalized. These bills are audited by CMS before they are released [27], and error rates in coding are audited by CMS for accuracy. We will track the timeline of requests to and responses from CMS.

10.3. End of year and just in time surveys

The CCC will administer the end of year surveys (trainees, faculty) and just in time surveys (trainees) using online survey software platforms such as Qualtrics or SurveyMonkey. The CCC will send the files with responses to the DCC for import into the data system; files related to tracking which recipients have not responded will not be forwarded to the DCC. The files will be uploaded to the iCOMPARE data system using a secure FTP portal customized to securely upload and tag (date, time, source, and operator) data elements into the data management system. The data transmitted to the DCC will not include individual level identifiers but will include program level identifiers.

10.4. Data provided by ACP, ACGME, and APDIM

The data management system will import and merge data files from the ACP, ACGME and APDIM into the master iCOMPARE database. The files will be uploaded using a secure FTP portal customized to securely upload and tag (date, time, source, and operator) data elements into the system. The data transmitted to the DCC from ACP, ACGME and APDIM will have been stripped of personal identifiers before transmission but will be identifiable at the program level.

10.5. Time and Motion Substudy data

The data system will import and merge data from Time and Motion Substudy demographics survey and the observation files into the master iCOMPARE database. The files are uploaded to the system using a secure FTP portal customized to securely upload and tag (date, time, source, and operator) data elements into the system.

The iCOMPARE Time and Motion Substudy survey and observation data will be identified at the intern level by study identification number rather than name or other personal identifier. Each staff member observing an intern or transferring data will also be assigned a unique iCOMPARE ID number and this ID will be associated with data entered or uploaded to the system.

10.6. Sleep and Alertness Substudy data

Interns participating in the Sleep and Alertness Substudy will be asked to continuously wear a wrist actigraph for 14 consecutive days. They will also receive a Smartphone to complete a brief survey and perform a 3-minute Psychomotor Vigilance Test (PVT) on the Smartphone, once each morning of the 14-day period. Data from the actiwatch will be transferred to a Smartphone app once daily, which will then automatically and remotely transfer the data back to Pulsar Infomatics, where the data will be stored on a secure server. The survey and PVT data also will be automatically transferred to Pulsar (via the Smartphone) after completion each morning. Interns will be contacted when actigraphy, survey, and/or PVT data are not received. Pulsar will send the data to members of the research team (Dr. David F Dinges and Dr. Mathias Basner) at the CCC for quality control purposes. The quality control process assures that interns are compliant (i.e., fill out surveys and perform the PVT each morning), and that there are no technical issues with the equipment (i.e., that valid data are collected). Sleep times will be extracted from the wrist actigraph and sleep survey data by Pulsar staff who are blind to **Curr** and **Flex** conditions. Because the extraction involves a human judgment of when daily sleep occurred relative to combining the two sources of data, CCC sleep experts at the University of Pennsylvania will do a final review of the extracted sleep times, blind to condition, after Pulsar de-identifies the data. Based on previous trials completed by the investigators, it is anticipated that less than 5% of the extracted sleep and wake times will require reclassification. The final extracted sleep times derived while blind to condition will be analyzed by the DCC.

The data themselves will not be analyzed by members of the CCC. The Smartphone will have a data plan only (i.e., no calling capability). The Smartphone is configured and managed by a secure role-based permission system. Administrative access to the app configuration and data management functions are granted to administrators with user-specific accounts and passwords. Administrator authentication is performed against a central server. Data are securely transmitted from the app to a central data collection server using 128-bit SSL encryption. The sole participant identifier used by the app, and associated with all data collected by the app, is a numeric participant ID. The data management system will import and merge data from the Sleep and Alertness Substudy surveys and actigraphy files into the master iCOMPARE database. The files will be uploaded to the data management system using a secure FTP portal customized to securely upload and tag (date, time, source and operator) data elements in to the data management system.

The iCOMPARE actigraphy and sleep survey data will be identified using study IDs rather than personal identifiers. Each staff member and each participant will be assigned a unique iCOMPARE ID number and this ID will be associated with data entered or uploaded to the data management system.

---

11. Biostatistical considerations

11.1. Sample size and power

We approached the statistical design by designating the non-inferiority mortality hypothesis as the primary hypothesis for which sample size calculations were based. The PASS 11 software for sample size and power analysis was used to calculate the sample size required for the mortality hypothesis. The PASS 11 software is well suited for iCOMPARE since it has implemented the complex statistical calculations needed to allow for superiority or non-inferiority hypotheses, and correlations in responses such as those we will see due to clustering on the IM programs. Our primary outcome (H1a; 30-day mortality) is based on a noninferiority hypothesis. The estimated 30-day mortality rate [2007/2008 data; personal communication from Dr. Silber] in the iCOMPARE target population was 11%, (11.1% in 2007 and 11.5% in 2008) and an SD for the pairs of rate differences of 1.5%. The consensus noninferiority mortality margin among the iCOMPARE investigators was assumed to be 1%. The 30-day mortality outcome measure is defined, for each IM program, as the difference between the 30-day mortality rate in the trial year minus the 30-day mortality rate in the pre-trial year. This approach permits the use of a simple model (two-sample t-test) for the set of at least N=29 pairs of test year vs. pre-test year differences in each group (**Curr** vs. **Flex**) in annual 30-day mortality that obviates the need for complex risk adjustment models, since it adjusts each outcome for secular trends in 30-day mortality as well as in IM program population risk profiles that are likely to cancel out by comparing successive years. The variability (pooled standard deviation (SD)) of each of the paired mortality rate differences was estimated using Medicare data from 2007/2008 for the population target IM programs. We performed the calculations with both 80% and 90% power to gauge any gains in power by recruiting beyond the N=58 IM programs required for 80% power. The results of the calculations for mortality noninferiority from PASS 11 are as follows, where Type-1 error (alpha) is based on a one-sided test as is appropriate for a non-inferiority design [30].

		Non-inferiority Margin	Actual Difference	Significance Level		Standard Deviation 1 (Curr)	Standard Deviation 2 (Flex)
Power	N1 (Curr) /N2 (Flex)	(NIM)	(D)	(Alpha)	Beta	(SD1)	(SD2)
0.8059	29/29	0.01	0	0.05	0.1941	0.015	0.015
0.9050	40/40	0.01	0	0.05	0.0950	0.015	0.015

Although sample size calculations were based on the mortality outcome, this number of programs will give excellent power for other study hypotheses. The 58 randomized programs are expected to include 4640 internal medicine residents: 1740 interns (approximately 30 interns per program) and approximately 1450 PGY2 trainees (approximately 25 PGY2 per program) and 1450 PGY3 trainees (approximately 25 PGY3 per program). Each program will include one program director (total of 58) and approximately 10 associated faculty (total of about 580 faculty). For example, for H2b, with 90% power, Type I error of 0.05, and minimum superiority mean difference 0.2 SD (0.14 points on the 5-point educational satisfaction scale), the required sample size is N=1052 interns. For H3a, with 90% power, one-sided Type I error of 0.05, and a noninferiority margin of 0.5 hours, the required sample size is 290 interns. The proposed sample sizes are higher: 1740 interns (30 at each of 58 programs) for H2b and 384 interns (48 at each of 8 programs) for H3a. Student's t-tests were used in the calculations to approximate the results from the mixed effects regression models proposed for analyses for H2b.

11.2. Data analysis

11.2.1. Overview

The DCC will work with iCOMPARE leadership to develop a statistical analysis plan (SAP) to supplement the analyses proposed here. The SAP will cover, in detail, the methods to be used to address the primary hypothesis and the 10 secondary hypotheses. These will include methods for descriptive, primary, secondary, and sensitivity analyses. The SAP will also specify methods for handling missing data (descriptive patterns of missing-ness, likelihood methods, sensitivity analyses with varying missing-ness assumptions–such as best case, worst case, and multiple imputation). Analyses to determine the consistency of effects across subgroups of trainees and IM programs will be specified in the SAP prior to conducting the analyses.

All primary analyses will be based on the "intention to treat" principle. Every effort will be made to collect data at the protocol-defined measurement time points, even for programs or participants who have discontinued the intervention. In general, non-inferiority tests will be one-sided and superiority tests will be two-sided. Two DCC biostatisticians will independently perform the primary analyses and resolve any discrepancies in results.

Since program directors at programs assigned to **Flex** have considerable latitude in design of schedules, we expect variation amongst the duty hour schedules followed in the Flex group. Information on the actual schedules implemented will be collected and the nature of the schedules and the degree of difference from **Curr** schedules will be characterized.

11.2.2. Patient safety and costs

11.2.2.1.1. Patient safety hypothesis H1a - 30-day mortality

The primary outcome will be the difference in the pre-trial year and trial year mortality rates. The SD of the set of paired annual differences (2008 vs. 2007) in 30-day mortality from the preliminary data was equal to 1.5%. The mortality rates were similar across the two years: 11.1% and 11.5% for 2007 and 2008, respectively, consistent with minimal secular trends in mortality. The noninferiority sample size calculations described above show high power and low one-sided type-I error for the noninferiority hypothesis with a 1% margin.

The program level data needed for Specific Aim 1 outcome measures (patient safety and costs) will be aggregated into rates or other measures at the program level across two 1-year periods – the rates in the pre-trial year and the rates in the year of the trial. The outcome measure will be the change in these rates from the pre-trial year to the trial year and will be compared by treatment group using the same non-inferiority test proposed in the sample size justifications above.

The model (model A1) is:

$Y_i = \gamma + \beta_1 x_i + \varepsilon_i, \ \ i = 1, \ldots, n_p \ \ where$
$Y_i =$ Outcome measure in IM program i ,
$\gamma =$ the intercept in the reference group (Curr),
$\beta_1 =$ the difference in intercepts between Flex and Curr,
$x_i = 1$ if the ith IM program is in **Flex**, 0 if the ith IM program is in **Curr**
$\varepsilon_i =$ i.i.d. random Gaussian errors with mean 0 and variance σ^2
$n_p =$ Number of clusters (IM programs)

Tests of β_1 (or $\beta_1 - nim$, where *nim* is the noninferiority margin) estimated using linear regression will be used to test this hypothesis, since β_1 is the expected difference in outcome: **Flex** vs. **Curr**. All randomized programs will be included in this model and we expect no missing data.

11.2.2.1.2. Other safety hypotheses (H1b-H1e)

The following outcomes are the remaining outcomes for the patient safety and cost hypotheses:

a) Patient safety and costs hypothesis H1b:
   - Measure: 7-day and 30-day hospital readmission rates
   - Non-inferiority margin: 1%

b) Patient safety and costs hypothesis H1c:
   - Measure: complications rates, defined by selected AHRQ Patient Safety Indicators
   - Non-inferiority margin: 1%

c) Patient safety and costs hypothesis H1d:
   - Measure: The rate of prolonged length of hospital stay
   - Non-inferiority margin: 1%

d) Patient safety and costs hypothesis H1e:
   - Measure: Overall resources utilized and Medicare payments for patient care
   - Non-inferiority margin: 1%

Analyses for H1b-e will use the same approach described for model A1 with either linear, logistic, or Poisson models depending on whether the outcome measure is measured/ordered, a proportion, or a count. The model estimates, 95% CIs, and p-values will be derived using Stata, R or SAS.

11.2.3. Trainee education hypotheses (H2a-H2d)

The following outcomes are the outcomes for the trainee education hypotheses:

a) Education hypothesis H2a:
   - Measure: Direct patient care and education measured from Time and Motion Substudy, specifically percent of time spent by the intern in direct patient care
   - Minimum important difference is 3% (0.75 SD)

b) Education hypothesis H2b:

- Measure: Trainee satisfaction with their educational experience measured from surveys, primarily the trainee's perception of having an 'appropriate balance for education' on an ordinal scale and is expected to have a mean of 3.7 (SD 0.7) in the **Curr** schedule [31-34]
- Minimum important difference is 0.175

c) Education hypothesis H2c:
- Measure: Faculty satisfaction with their clinical teaching experiences measured from surveys, primarily the faculty ranking on 'residents workload exceeds capacity to do the work' from the ACGME survey measured on an ordinal scale with expected mean in the **Curr** schedule of 4.1 (SD 0.7) [31-34]
- Minimum important difference is 0.175

d) Education hypothesis H2d:
- Measure: Standardized test scores for interns on the In-Training Examination (ITE) measured as the percent correct with expected the mean score in the **Curr** schedule of approximately 65 (SD = 18) [Lisa Bellini, personal communication].
- Noninferiority margin is 2%

The trainee education analyses will be modeled using the model (model A2):

$Y_{ij} = \gamma_i + \beta_1 x_{ij} + \varepsilon_{ij}, \; i = 1, \dots, n_p; \; j = 1, \dots, n_i, \quad where$
$Y_{ij} =$ Mean outcome measure in IM for intern (or faculty or director) j in program i ,
$x_{ij} = 1$ if the ith IM program is in **Flex**, 0 if the ith IM program is in **Curr**
$\gamma_i =$ i.i.d. random Gaussian intercept for the IM program i with mean β_0 and variance σ_1
$\beta_1 =$ difference in intercepts in Flex and Curr
$\varepsilon_{ij} =$ i.i.d. random Gaussian errors with mean 0 and variance σ^2
$n_p =$ Number of clusters (IM programs)
$n_i =$ Number of interns in program i

Note that γ_i is the random intercept needed to account for clustering. Model A2 is a multilevel mixed effects model that may be estimated using the Stata software mixed command with REML estimates, R (lme4 package) or SAS (PROC MIXED). The hypotheses will be tested using model A2 with either linear, logistic, or Poisson mixed effects models depending on whether the outcome measure is measured/ordered, a proportion, or a count.

11.2.4. Intern **sleep and alertness hypotheses (H3a-H3b)**

The following outcomes are the outcomes for the intern sleep and alertness hypotheses:

a) Sleep hypothesis H3a:
- Measure: Average daily sleep measured by a 14-day period of sleep monitoring using actigraphy (verified by daily sleep diaries) with expected average sleep in **Curr** of 6.946 hours (SD=1.451 hours) [David Dinges, personal communication].
- Non-inferiority margin is 0.5 hours.

b) Sleep hypothesis H3b:
- Measure: Average subjective sleepiness measured by Karolinska Sleepiness Scale (KSS) [24]
- Non-inferiority margin: 1 unit on KSS Likert scale

The intern sleep and alertness hypotheses will be tested using Model A2 described above.

---

12. Data monitoring

iCOMPARE data and safety will be monitored by the Steering Committee and by an independent Data and Safety Monitoring Board (DSMB) as required by NIH guidelines for multicenter trials. The Steering Committee will monitor accumulating safety and performance data. The DSMB will also monitor safety and performance data. The DSMB members will be appointed by NHLBI. The DSMB will be advisory to the NHLBI. NHLBI will provide investigators with a summary report after each DSMB meeting, with recommendations for the trial. Program directors will forward these recommendations to their site's IRB or the IRB of record for the trial. The University of Pennsylvania IRB has agreed to function as a central IRB for the trial, and sites may choose to use the central IRB or their own institutional IRB.

The Steering Committee will monitor accumulating safety and performance data at regularly scheduled intervals to help assure participant safety and for quality assurance. During the implementation stage of the trial, the Steering Committee will monitor the 1) timeline and progress of refinement of the protocol and other study documents, survey development, database development; 2) enrollment of programs; 3) attainment of IRB approval at each participating site; and 4) training of study staff. As data collection begins, the Steering Committee will begin to monitor progress of 1) harvesting of data from Pulsar on the intern sleep measures and ACGME, ACP, and APDIM for education measures; 2) harvesting of data from time and motion observation sessions; 3) completion of surveys by trainees and faculty; 4) data requests and receipts from Medicare; and 5) reports of safety concerns. Reports of safety concerns may be received by the CCC or DCC directly from site staff or as noted by investigators upon review of performance data reports; reports of concerns will be reviewed by the CCC and DCC directors upon receipt and will be reviewed by the Steering Committee in a timely fashion.

The DSMB will review the protocol for the iCOMPARE trial and make recommendations to the NHLBI regarding content and trial activities. Once the trial starts, the DSMB will monitor the accumulating performance data and review education and sleep outcomes acquisition and quality. Reports may include data tables, graphs, and figures and will include the most recent data available at the time the report was prepared or analyses completed. The patient safety and cost outcomes (mortality, length of stay, complications, readmissions) are generated from Medicare data, and each calendar year of claims data is generally available 9 months after the end of the relevant calendar year. Because of this delay, the DSMB will not review any interim patient safety and cost outcomes. The DSMB charter will include more information on data monitoring.

13. Ethics

13.1. Research ethics approval

The University of Pennsylvania IRB (Penn IRB) has agreed to be the IRB of record for all iCOMPARE centers and programs that wish to use a central IRB. Individual Internal Medicine training programs may choose to use the Penn IRB for that purpose or they may seek approval from their local institutional review board. If a center or program elects to use the Penn IRB, then documentation of both the local IRB's acceptance of this arrangement and documentation of the Penn IRB's acceptance of the responsibility for that center or program are required.

IRB approvals will be monitored by the CCC. Protocol amendments and changes to the consent forms and other study documents will be distributed from the CCC to the Penn IRB and to the programs that are using their local IRBs.

13.2. Consent

13.2.1. Randomization

The decision to participate in iCOMPARE and to be randomized to duty hour schedule will be made by the program director and other leadership at each participating program. The trainees and faculty in the programs do not consent to randomization—they will follow the decision made by their governing person or group. These approaches are consistent with routine operations of residency programs, in which program directors decide on program structure.

13.2.2. Consent for use of data provided by ACGME, APDIM and ACP

The trainees and faculty also do not consent to use of their ACGME and APDIM survey responses by iCOMPARE, nor do trainees consent to iCOMPARE's use of their ITE data obtained from ACP. ACGME, APDIM and ACP will provide data identifiable at the program level but not at the respondent level. The Penn IRB has granted iCOMPARE waiver of the requirement to obtain informed consent from trainees and faculty for these data under HHS regulations at 45 CFR 46.116(c). The Penn IRB recognized that iCOMPARE could not practicably be carried out without the waiver and is designed to study, evaluate, or otherwise examine possible changes in or alternatives to current standards for graduate medical education. Thus iCOMPARE meets the criteria for waiver of consent.

13.2.3. Consent for use of patient data

Patient data used to test iCOMPARE hypotheses will be limited to Medicare claims data. All requests for Medicare claims data are made through the University of Minnesota Research Data Assistance Center (ResDAC; http://www.resdac.org/). There will be no direct data collection by iCOMPARE from patients. ResDAC requires these approvals before approving release of Medicare data to the requestor: IRB approval of the proposed data analysis, approval of a Data Use Agreement between CMS and the requestor, approval of the requestor's data management plan for protecting the data from abuse and inappropriate disclosure, and approval of the project and plans from the CMS Privacy Board.

The Penn IRB has granted iCOMPARE waiver of the requirement to obtain informed consent from patients for these data under HHS regulations at 45 CFR 46.116(c). The Penn IRB recognized that iCOMPARE could not practicably be carried out without the waiver and is designed to study, evaluate, or otherwise examine possible changes in or alternatives to current standards for graduate medical education. Thus iCOMPARE meets the criteria for waiver of consent.

13.2.4. Consent for end of year and just in time survey data

Trainees participating in iCOMPARE include interns (PGY1) and PGY2 and PGY3 trainees. Trainees providing data to iCOMPARE can be divided into two groups. Group 1 are trainees at the participating programs in May 2015 and June 2015, and Group 2 are trainees at the participating programs in July 2015 through June 2016.

In May through June 2015, Group 1 trainees will be asked to complete iCOMPARE assessments querying their attitudes and burnout. The surveys will be conducted via email to their IM program email address; the email will include a link to the survey. Participation will be encouraged, but voluntary, and tacit consent will be used for these surveys. Additionally, de-identified data from the ACGME end-of-year survey of Group 1 trainees (conducted in May 2015) will be provided by the ACGME to iCOMPARE. The ACP has agreed to provide iCOMPARE with de-identified In-Training Examination (ITE) scores for Group 1 interns.

Group 2 trainees will participate in iCOMPARE from June 2015 through June 2016. Group 2 trainees at participating programs will be given an introduction to the trial during orientation weeks in June 2015. These trainees will be asked to complete assessments querying their attitudes and burnout at the end of the intervention year. Additionally, Group 2 interns will be periodically surveyed about their educational and clinical experiences during the intervention year while on key study rotations (just in time surveys). All of these surveys will be emailed to the trainees' program email addresses; the email will include a link to the survey. Participation will be encouraged, but voluntary, and tacit consent will be used for these surveys.

13.2.5. Consent for Time and Motion Substudy

Interns participating in the Time and Motion Substudy will provide written consent to permit observers to follow them for a subset of their work periods for time in motion assessments. Prior to start of the substudy at each of the 6 participating sites, we will explain to the interns that a sample of interns on pre-specified rotations will be asked to consent to being observed during their work on the rotation. Before obtaining consent, interns will be given opportunities to ask questions and will be informed that they may ask for the observation to stop at any time—or to pause if for any reason more personal privacy is desired. We will emphasize that the choice to consent is their own and their decision will have no consequences in terms of training assignments or evaluations. Site coordinators and Program Directors will facilitate the interns' participation in the Time and Motion Substudy, but they will be instructed not to influence whether an intern elects to participate or not participate in the substudy. Interested and willing interns will be asked to provide written consent to participate in this portion of the study. Participation will be voluntary and written consent will be obtained. An iCOMPARE staff person will be responsible for obtaining consent.

13.2.6. Consent for Sleep and Alertness Substudy

Interns participating in the Sleep and Alertness Substudy will provide written consent to perform actigraphy and Smartphone assessments (i.e., sleep, sleepiness, and PVT performance) for a two week period in an iCOMPARE chosen rotation. Prior to start of the substudy, the site coordinator will explain to the interns in the programs recruited for the substudy that during some months of the year, a sample of interns on pre-specified rotations will be asked to consent to provide 14-day periods of data while on the specified rotations. Participating interns will be given opportunities to ask questions of the study team prior to being asked to provide consent. We will emphasize that the choice to consent is their own and their decision will have no consequences in terms of training assignments or evaluations. Site coordinators and Program Directors will facilitate the interns' participation in the Sleep and Alertness Substudy, but they will be instructed not to influence whether an intern elects to participate or not participate in the substudy. Interns will be told that they are not responsible for equipment loss or damage, with the exception that should either occur they should inform the site coordinator and study team as soon as possible. Interested and willing interns will be asked to provide written consent to participate in this portion of the study. An iCOMPARE staff person will be responsible for obtaining consent.

13.3. Protections against risk

13.3.1. Overview

Potential risks are described below. Overall the risk benefit ratio is favorable given the long term potential of this study to significantly contribute to our knowledge of the impact of duty hour rules on patient safety and cost outcomes, education and performance outcomes, and intern sleepiness and alertness outcomes.

13.3.2. Patient safety and costs

For patients, we use Medicare claims data to analyze clinical outcomes. Analysis of these administrative data, which are routinely collected, is felt to be the least intrusive method of measuring these outcomes and, given high standards of information security described below, also the most secure.

13.3.3. Trainee education

For trainees in general, iCOMPARE will collect educational assessments that are individually de-identified and so should present little to no risk to confidentiality. Similarly, information provided by faculty will also be de-identified at the respondent level. All program directors will communicate to their trainees that participation in the iCOMPARE surveys will have no effect on their trainee assignments or evaluations. Program directors will be masked to survey completion status and to responses of individual trainees and faculty.

13.3.4. Intern sleep and alertness

To mitigate risks of fatigue, all trainees will be required to receive structured education in sleep deprivation and fatigue management in June 2015.

13.4. Confidentiality

The Medicare claims data files received from ResDAC will be Research Identifiable Files (RIF); these files contain beneficiary level protected health information. ResDAC requires a Data Use Agreement and review of the application for the files by the CMS Privacy Board to ensure that the beneficiary's privacy is protected and the need for identifiable data is justified. Prior to approval to receive the data, ResDAC also reviews and must approve the iCOMPARE data management plan for protecting the confidentiality of the files at the recipient's site. iCOMPARE will not be allowed to purchase the files from ResDAC without approval of the data use agreement, approval from the Privacy Board, and approval of the data management plan. Per that plan, individual-level data for patients will be kept confidential and stored only on the highly secure servers available at the Children's Hospital of Philadelphia; storage on personal computers or laptops is prohibited. Only authorized project personnel will have access to the data as overseen by the DCC staff at CHOP.

---

14. Dissemination and data sharing

14.1. Data sharing

Data to test the iCOMPARE hypotheses will come from interns, PGY2 and PGY3 residents, faculty, program directors, and patients and will include data collected directly by iCOMPARE as well as data collected by other sources originally for other purposes and now leveraged by iCOMPARE for a new purpose (e.g., Medicare data will be used to assess patient mortality; ITE test scores for trainees will be obtained from the ACP and will be used to assess education outcomes; end of year questionnaires for trainees and faculty will be obtained from the ACGME and used to assess training quality). Some of these data will be at the individual level and some will be group level data.

Where iCOMPARE collects the data by survey, the survey will include a statement that de-identified data from the survey will be deposited in a public repository at the end of the study. A respondent may opt out of the survey if unwilling to accept the terms of use. The consent statements for the Time and Motion Substudy and for the Sleep and Alertness Substudy will include consent to share de-identified data. Where data collected by another group are provided to iCOMPARE, de-identified data will be requested.

All iCOMPARE investigators will be given access to cleaned datasets of data by the end of the trial funding. The DCC will prepare de-identified datasets by the end of the funding period for deposit at the NHLBI BioLINCC repository (https://biolincc.nhlbi.nih.gov/home/).

14.2. Dissemination of study results

We will aim for dissemination of results through the traditional academic channels of journal publication and presentation at scientific meetings as well as through news media regardless of the direction of the results. We will also partner with the Leonard Davis Institute of Health Economics — an institute at the University of Pennsylvania that connects its School of Medicine (Perelman) to its business school (Wharton) and Schools of Nursing, Law, and Communication (Annenberg) — to extend the reach of our findings to members of Congress and leaders in health care who are unlikely to receive or read academic journals but who would value the results of this trial and are in positions to create change in other relevant areas.

In addition to public dissemination through media outlets, we will post the summary results on clinicaltrials.gov.

The ACGME is not expected to participate in study publications but may assist with dissemination of results, once they are determined and published.

15. Tables

15.1. Trial organization

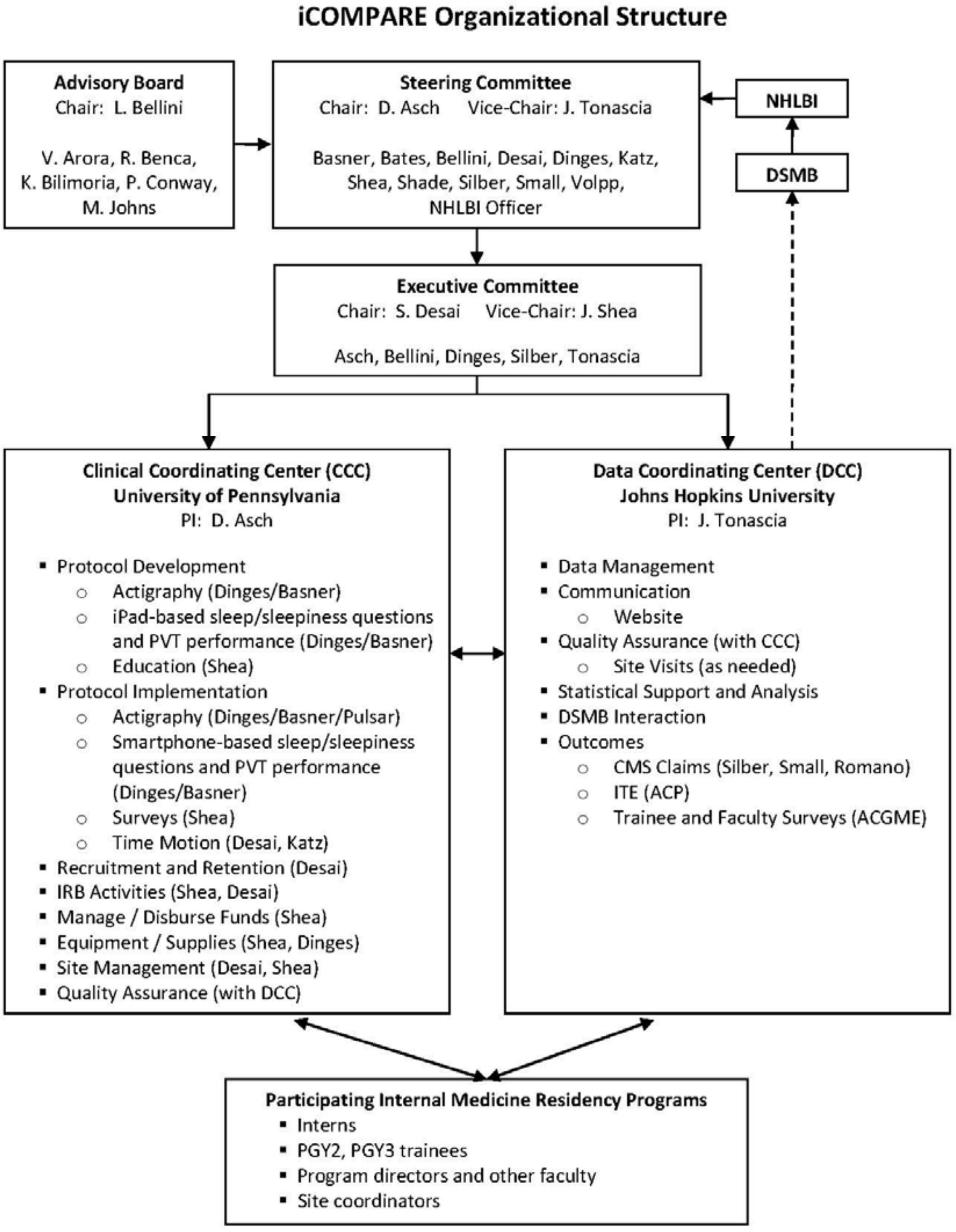

Note: The ACGME provides funding to iCOMPARE but does not participate in its conduct nor data analysis.

15.2. Committees and centers

Steering Committee

- Major decision making body of iCOMPARE
- Provides oversight in study planning, conduct and dissemination of findings
- Votes on all important decisions and approves the final protocol and any subsequent amendments
- Maintains relationship with the iCOMPARE Advisory Board and funding agencies
- Consists of core study team members
- Chaired by the PI of the Clinical Coordinating Center (CCC); PI of the Data Coordinating Center is vice-chair
- Meets monthly

Executive Committee

- Manages day-to-day major issues of iCOMPARE and makes decisions between Steering Committee meetings
- Organizes and sets agenda for Steering Committee meetings
- Provides oversight of study operations
- Consists of leaders of operations, education, and safety sub-teams and the PIs of the CCC and DCC
- Chaired by the operations team leader; vice chair is the education team leader

Clinical Coordinating Center (CCC)

- Based at the University of Pennsylvania and led by David Asch
- Responsible for protocol implementation and data capture
- Fiscal and analytic firewall between CCC and DCC

Data Coordinating Center (DCC)

- Based at Johns Hopkins University and led by James Tonascia
- Responsible for data management and analysis
- Fiscal and analytic firewall between CCC and DCC

Research Group

- Conducts the iCOMPARE trial per the protocol approved by the Steering Committee
- Provides input and feedback to the Steering Committee
- Consists of the participating Internal Medicine program leaders, the trainees providing data in the trial, all members of all iCOMPARE committees, all CCC staff and all DCC staff

Advisory Board

- Advisory to the Steering Committee and appointed by the Steering Committee
- Provides input and feedback on study design and outcomes to the Steering Committee
- Consists of leaders in the field of graduate medical education
- Chaired by Lisa Bellini

15.3. Design synopsis

Trial name

- individualized Comparative Effectiveness of Models Optimizing Patient Safety and Resident Education (iCOMPARE)

Overall objective and approach

- Conduct a cluster randomized trial to compare 2 duty hour schedules with respect to:
  - Patient safety and costs outcomes
  - Trainee education outcomes
  - Intern sleep and alertness outcomes
- Randomize Internal Medicine (IM) training programs to duty hour schedule
- Collect new data directly during the trial and leverage data collected by other sources (e.g., ACGME, ACP, APDIM, Medicare) to test the trial's hypotheses

Treatment groups

- **Current (Curr, control)**: IM programs randomized to the currently mandated duty standards (maximum work duration of 16 hours for interns and 28 hours for PGY2-3); this schedule may involve night float
- **Flexible (Flex, intervention)**: IM programs randomized to intervention will be allowed to construct flexible duty hour schedules that comply with 3 rules, each averaged over 4 weeks:
  - No more than 80 hours of work per week
  - 1 day off in 7
  - In-house call no more frequently than every 3rd night
- The control and intervention schedules apply to all trainees (PGY1-3)
- The ACGME has granted a waiver allowing IM programs participating in iCOMPARE to follow the intervention schedule; the waiver encompasses all trainees on the IM teams, including trainees rotating from other departments

Randomization features

- **Randomization unit**
  - IM training program (cluster randomization)
  - Each trainee will follow the iCOMPARE duty hour schedule to which their IM program is randomized

Treatment assignment ratio: 1:1

Outcome ascertainment approaches

- Leverage other sources for data to test trial hypotheses (e.g., ACGME, ACP, APDIM, Medicare)
- Direct data collection from trainees, program directors, and program faculty via survey
- Direct data collection from interns participating in the Time and Motion Substudy via observation and interview
- Direct data collection from interns participating in the Sleep and Alertness Substudy via observation and interview

Outcomes (source)

- Patient safety and costs
  - 30-day mortality (Medicare data)
  - 7-day and 30-day readmission rates (Medicare data)
  - Complication rates defined by selected AHRQ Patient Safety Indicators (Medicare data)
  - Rate of prolonged length of stay (Medicare data)
  - Overall costs, as indicated by total Medicare payments (Medicare data)
- Trainee education and process outcomes
  - Intern work intensity, ownership, and continuity measures (iCOMPARE surveys)
  - Intern time in direct patient care and other activities (Time and Motion Substudy)
  - Trainee satisfaction, burnout, and attitudes (iCOMPARE and ACGME surveys)
  - Intern knowledge (ACP ITE score)
  - Faculty satisfaction with training and teaching experience (ACGME survey)
  - Program director satisfaction and perceptions of training safety, teamwork and supervision (iCOMPARE and APDIM surveys)
- Intern sleep and alertness outcomes (Sleep and Alertness Substudy)
  - Sleep-wake times (wrist actigraphy)
  - Onset and offset times of sleep periods in past 24h (interview)
  - Perceived sleepiness (Karolinksa Sleepiness Scale)
  - Behavioral alertness (psychomotor vigilance performance)

Sample size justification for mortality outcome

- Planned sample size: 58 graduate medical education training programs in Internal Medicine selected from ACGME list of candidate IM programs; the planned total number of hospitals across the training program clusters is 100+ (some programs span more than one hospital)
- Sample size determined to be adequate to test the hypothesis that 30-day mortality among Medicare beneficiaries in defined high risk DRGs (30-day mortality = 11%) in the intervention flexible schedule is not inferior to the corresponding mortality in the current 16 hour limit (control) schedule by a margin no greater than 1%
- Unit of analysis: IM training program
- Primary outcome measure: Difference (trial year vs. pre-trial year) in 1-year 30-day mortality
- Power: > 0.80
- Type I error (alpha): 0.05
- Primary analysis method: one-sided two-sample t-test for a noninferiority margin of 1%
- Software for sample size calculations: PASS 11

Recruitment goals

- 58 IM programs encompassing 100+ hospitals

Selection criteria for programs to be randomized

- Resident to bed ratio > 0.105 (excluded bottom 50% of hospitals by resident to bed ratio)
- Sufficient patient volume (excluded bottom 25% of hospitals by patient volume)
- Consent to participate
- Current ACGME accreditation

Consent issues

- ACGME has provided a waiver allowing programs to participate in COMPARE
- Programs: consent to randomization and obtain local IRB approval

- Hospitals: programs must provide evidence of buy in/consent of their hospitals
- Patients
  – They do not consent
  – They are not informed about the trial
  – Their data (in files provided by Medicare) are identified for analysis on the basis of the hospital seen at, their diagnosis, their calendar time of treatment (and possibly other factors)
- Trainees
  – Do not consent to randomization
  – Do not consent to use of ACGME and ACP data – data will be provided aggregated at the program level
  – Do provide consent for participation in Time and Motion Substudy
  – Do provide consent for participation in Sleep and Alertness Substudy
  – Completion of iCOMPARE surveys will be described as voluntary and participation will reflect tacit consent
- Program directors and faculty
  – Faculty do not consent to randomization; program directors consent to randomization on behalf of their program and institution and program directors are required to provide documentation of institutional official approval
  – Do not consent to use of ACGME survey responses – Data will be provided aggregated at the program level
  – Completion of iCOMPARE surveys will be described as voluntary and participation will reflect tacit consent

Key dates

- Spring 2014 – initiate recruitment of IM programs
- Fall 2014 - randomize IM programs
- 1Jul2015 - interns in participating IM programs begin to follow the COMPARE assigned duty hour schedule
- Winter 2016 – receive 2013 and 2014 CMS claims (baseline years)
- 30Jun2016 - trial ends
- Oct2016 - receive 1st release of 2015 CMS claims
- Jan2017 - receive final release of 2015 CMS claims
- Oct2017 - receive 1st release of 2016 CMS claims
- Jan2018 - receive final release of 2016 CMS claims
- Mar2019 - Primary outcome paper/dissemination of results and implications
- Jun2019 - End of funding

Mode of support

- Grant from NHLBI
- Grant from ACGME

Research group members

- Participating IM programs, program directors and program coordinators
- Trainees in the participating IM programs
- Faculty at the participating IM programs
- CCC
- DCC
- Advisory Board

15.4. Derivation of the study population (CONSORT diagram)

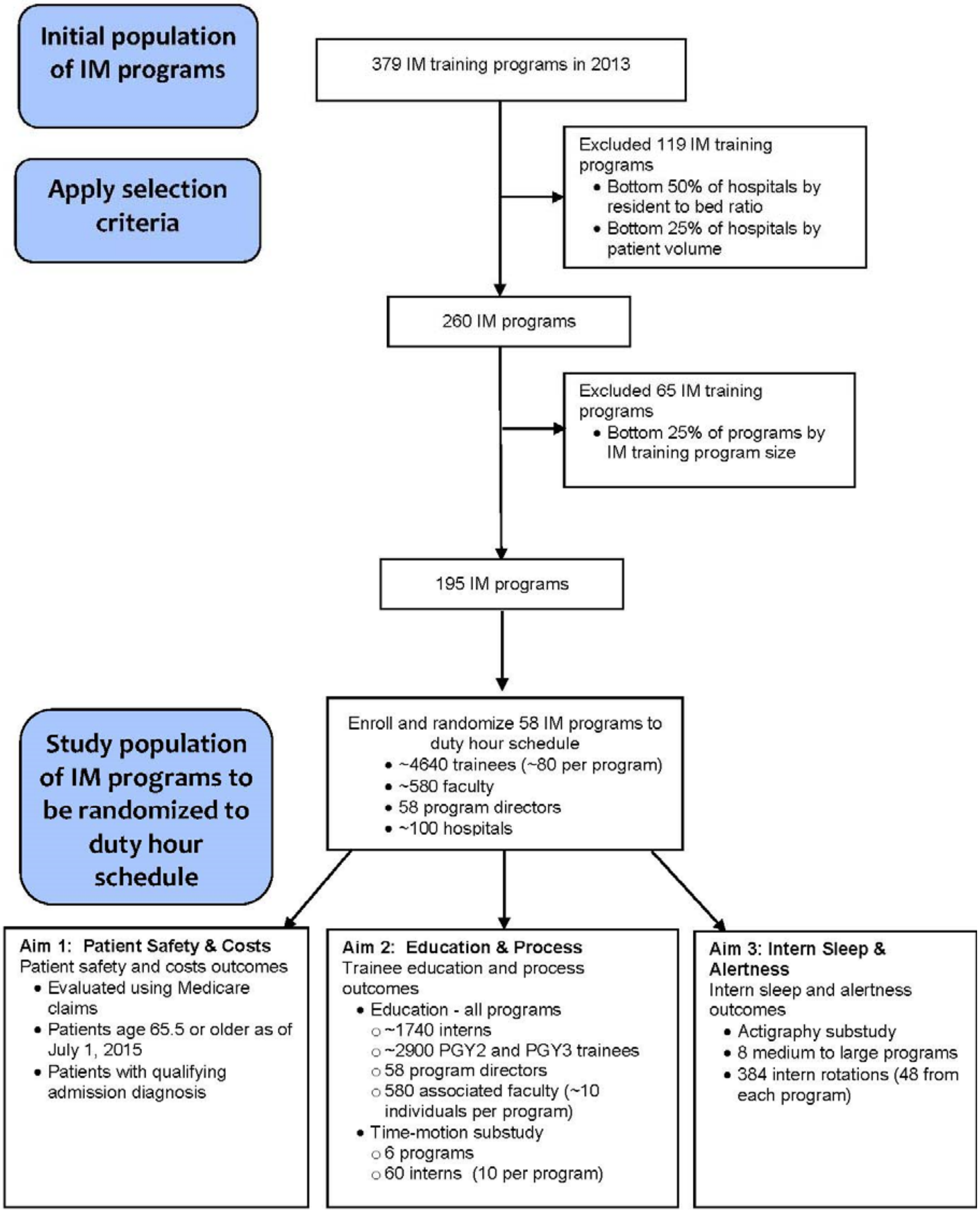

15.5. ICD-9 codes for qualifying principal diagnosis on hospital admission

Pneumonia:

481	Pneumococcal Pneumonia [Streptococcus Pneumoniae Pneumonia]
482	Other Bacterial Pneumonia
482.1	Pneumonia Due to Pseudomonas
482.2	Pneumonia Due to Hemophilus Influenzae (H. Influenzae)
482.3	Pneumonia Due to Streptococcus
482.31	Pneumonia Due to Streptococcus Group A
482.32	Pneumonia Due to Streptococcus Group B
482.39	Pneumonia Due to Other Streptococcus
482.41	Methicillin Susceptible Pneumonia Due to Staphylococcus Aureus
482.42	Methicillin Resistant Pneumonia Due to Staphylococcus Aureus
482.49	Other Staphylococcus Pneumonia
482.82	Pneumonia Due to Escherichia Coli [E.Coli]
482.83	Pneumonia Due to Other Gram-Negative Bacteria
482.84	Pneumonia Due to Legionnaires' Disease
482.89	Pneumonia Due to Other Specified Bacteria
482.4	Pneumonia Due to Staphylococcus
482.9	Bacterial Pneumonia Unspecified
483	Pneumonia Due to Other Specified Organism
483.1	Pneumonia Due to Chlamydia
483.8	Pneumonia Due to Other Specified Organism
485	Bronchopneumonia Organism Unspecified
486	Pneumonia Organism Unspecified

Stroke:

430	Subarachnoid Hemorrhage
431	Intracerebral Hemorrhage
432.0	Nontraumatic Extradural Hemorrhage
432.1	Subdural Hemorrhage
432.9	Unspecified Intracranial Hemorrhage
433.01	Occlusion and Stenosis of Basilar Artery with Cerebral Infarction
433.11	Occlusion and Stenosis of Carotid Artery with Cerebral Infarction
433.21	Occlusion and Stenosis of Vertebral Artery with Cerebral Infarction
433.31	Occlusion and Stenosis of Multiple and Bilateral Precerebral Arteries with Cerebral Infarction
433.81	Occlusion and Stenosis of Other Specified Precerebral Artery with Cerebral Infarction
433.91	Occlusion and Stenosis of Unspecified Precerebral Artery with Cerebral Infarction
434.01	Cerebral Thrombosis with Cerebral Infarction
434.11	Cerebral Embolism with Cerebral Infarction
434.91	Cerebral Artery Occlusion Unspecified with Cerebral Infarction
436	Acute but Ill-Defined Cerebrovascular Disease

AMI:

410.01	Acute Myocardial Infarction of Anterolateral Wall Initial Episode of Care
410.11	Acute Myocardial Infarction of Other Anterior Wall Initial Episode of Care
410.21	Acute Myocardial Infarction of Inferolateral Wall Initial Episode of Care
410.31	Acute Myocardial Infarction of Inferoposterior Wall Initial Episode of Care
410.41	Acute Myocardial Infarction of Other Inferior Wall Initial Episode of Care
410.51	Acute Myocardial Infarction of Other Lateral Wall Initial Episode of Care
410.61	True Posterior Wall Infarction Initial Episode of Care
410.71	Subendocardial Infarction Initial Episode of Care
410.81	Acute Myocardial Infarction of Other Specified Sites Initial Episode of Care
410.91	Acute Myocardial Infarction of Unspecified Site Initial Episode of Care

GI Bleed:

456.0	Esophageal Varices with Bleeding
530.7	Gastroesophageal Laceration-Hemorrhage Syndrome
530.82	Esophageal Hemorrhage
531.00	Acute Gastric Ulcer with Hemorrhage Without Obstruction
531.01	Acute Gastric Ulcer with Hemorrhage with Obstruction
531.20	Acute Gastric Ulcer with Hemorrhage and Perforation Without Obstruction
531.21	Acute Gastric Ulcer with Hemorrhage and Perforation with Obstruction
531.40	Chronic or Unspecified Gastric Ulcer with Hemorrhage Without Obstruction
531.41	Chronic or Unspecified Gastric Ulcer with Hemorrhage with Obstruction
531.60	Chronic or Unspecified Gastric Ulcer with Hemorrhage and Perforation Without Obstruction
531.61	Chronic or Unspecified Gastric Ulcer with Hemorrhage and Perforation with Obstruction
532.00	Acute Duodenal Ulcer with Hemorrhage Without Obstruction
532.01	Acute Duodenal Ulcer with Hemorrhage with Obstruction
532.20	Acute Duodenal Ulcer with Hemorrhage and Perforation Without Obstruction
532.21	Acute Duodenal Ulcer with Hemorrhage and Perforation with Obstruction
532.40	Chronic or Unspecified Duodenal Ulcer with Hemorrhage Without Obstruction
532.41	Chronic or Unspecified Duodenal Ulcer with Hemorrhage with Obstruction
532.60	Chronic or Unspecified Duodenal Ulcer with Hemorrhage and Perforation Without Obstruction
532.61	Chronic or Unspecified Duodenal Ulcer with Hemorrhage and Perforation with Obstruction
533.00	Acute Peptic Ulcer of Unspecified Site with Hemorrhage Without Obstruction
533.01	Acute Peptic Ulcer of Unspecified Site with Hemorrhage with Obstruction
533.20	Acute Peptic Ulcer of Unspecified Site with Hemorrhage and Perforation Without Obstruction
533.21	Acute Peptic Ulcer of Unspecified Site with Hemorrhage and Perforation with Obstruction
533.40	Chronic or Unspecified Peptic Ulcer of Unspecified Site with Hemorrhage Without Obstruction
533.41	Chronic or Unspecified Peptic Ulcer of Unspecified Site with Hemorrhage with Obstruction
533.60	Chronic or Unspecified Peptic Ulcer of Unspecified Site with Hemorrhage and Perforation Without Obstruction
533.61	Chronic or Unspecified Peptic Ulcer of Unspecified Site with Hemorrhage and Perforation with Obstruction
534.00	Acute Gastrojejunal Ulcer with Hemorrhage Without Obstruction
534.01	Acute Gastrojejunal Ulcer with Hemorrhage with Obstruction
534.20	Acute Gastrojejunal Ulcer with Hemorrhage and Perforation Without Obstruction
534.21	Acute Gastrojejunal Ulcer with Hemorrhage and Perforation with Obstruction
534.40	Chronic or Unspecified Gastrojejunal Ulcer with Hemorrhage Without Obstruction

534.41	Chronic or Unspecified Gastrojejunal Ulcer with Hemorrhage with Obstruction
534.60	Chronic or Unspecified Gastrojejunal Ulcer with Hemorrhage and Perforation Without Obstruction
534.61	Chronic or Unspecified Gastrojejunal Ulcer with Hemorrhage and Perforation with Obstruction
535.01	Acute Gastritis with Hemorrhage
535.11	Atrophic Gastritis with Hemorrhage
535.21	Gastric Mucosal Hypertrophy with Hemorrhage
535.31	Alcoholic Gastritis with Hemorrhage
535.41	Other Specified Gastritis with Hemorrhage
535.51	Unspecified Gastritis and Gastroduodenitis with Hemorrhage
535.61	Duodenitis with Hemorrhage
537.83	Angiodysplasia of Stomach and Duodenum with Hemorrhage
562.02	Diverticulosis of Small Intestine with Hemorrhage
562.03	Diverticulitis of Small Intestine with Hemorrhage
562.12	Diverticulosis of Colon with Hemorrhage
562.13	Diverticulitis of Colon with Hemorrhage
569.3	Hemorrhage of Rectum and Anus
569.85	Angiodysplasia of Intestine with Hemorrhage
578.0	Hematemesis
578.1	Blood In Stool
578.9	Hemorrhage of Gastrointestinal Tract Unspecified

CHF:

398.91	Rheumatic Heart Failure (Congestive)
402.01	Malignant Hypertensive Heart Disease with Heart Failure
402.11	Benign Hypertensive Heart Disease with Heart Failure
402.91	Unspecified Hypertensive Heart Disease with Heart Failure
404.01	Hypertensive Heart and Chronic Kidney Disease Malignant with Heart Failure with Chronic Kidney Disease Stage I Through Stage Iv or Unspecified
404.03	Hypertensive Heart and Chronic Kidney Disease Malignant with Heart Failure with Chronic Kidney Disease Stage V or End Stage Renal Disease
404.11	Hypertensive Heart and Chronic Kidney Disease Benign with Heart Failure with Chronic Kidney Disease Stage I Through Stage Iv or Unspecified
404.13	Hypertensive Heart and Chronic Kidney Disease Benign with Heart Failure with Chronic Kidney Disease Stage V or End Stage Renal Disease
404.91	Hypertensive Heart and Chronic Kidney Disease Unspecified with Heart Failure with Chronic Kidney Disease Stage I Through Stage Iv or Unspecified
404.93	Hypertensive Heart and Chronic Kidney Disease Unspecified with Heart Failure with Chronic Kidney Disease Stage V or End Stage Renal Disease
428	Heart Failure
428.0	Congestive Heart Failure Unspecified
428.1	Left Heart Failure
428.20	Unspecified Systolic Heart Failure
428.21	Acute Systolic Heart Failure
428.22	Chronic Systolic Heart Failure
428.23	Acute on Chronic Systolic Heart Failure
428.30	Unspecified Diastolic Heart Failure
428.31	Acute Diastolic Heart Failure
428.32	Chronic Diastolic Heart Failure
428.33	Acute on Chronic Diastolic Heart Failure

428.40	Unspecified Combined Systolic and Diastolic Heart Failure
428.41	Acute Combined Systolic and Diastolic Heart Failure
428.42	Chronic Combined Systolic and Diastolic Heart Failure
428.43	Acute on Chronic Combined Systolic and Diastolic Heart Failure
428.9	Heart Failure Unspecified

Septicemia:

038	Septicemia
038.0	Streptococcal Septicemia
038.1	Staphylococcal Septicemia
038.9	Unspecified Septicemia

Kidney Failure:

584	Acute Kidney Failure
584.9	Acute Kidney Failure Unspecified

Cardiac:

427.31	Atrial Fibrillation
427.41	Ventricular Fibrillation
427.5	Cardiac Arrest

COPD:

490	Bronchitis Not Specified as Acute or Chronic
491.21	Obstructive Chronic Bronchitis with (Acute) Exacerbation
491.22	Obstructive Chronic Bronchitis with Acute Bronchitis

Pancreatitis:

577.0	Acute Pancreatitis
577.9	Unspecified Disease of Pancreas

Acute Respiratory Failure:

518.81	Acute Respiratory Failure
518.84	Acute and Chronic Respiratory Failure
518.89	Other Diseases of Lung Not Elsewhere Classified
519.11	Acute Bronchospasm

Chest Pain:

786.5	Chest Pain
786.59	Other Chest Pain

Cellulitis:

682	Other Cellulitis and Abscess
682.6	Cellulitis and Abscess of Leg Except Foot

Coronary Atherosclerosis:

414.01	Coronary Atherosclerosis of Native Coronary Artery
414.1	Aneurysm and Dissection of Heart

Pulmonary Embolism:

415	Acute Pulmonary Heart Disease
415.12	Septic Pulmonary Embolism
415.19	Other Pulmonary Embolism and Infarction

Syncope:

780.0	Alteration of Consciousness
780.2	Syncope and Collapse
780.3	Convulsions
780.01	Coma

Intestinal Infection:

008	Intestinal Infections Due to Other Organisms
008.45	Intestinal Infection Due to Clostridium Difficile
008.49	Intestinal Infection Due to Other Organisms

Obstructive Asthma:

493.9	Asthma Unspecified
493.22	Chronic Obstructive Asthma with (Acute) Exacerbation

Bronchitis:

494.1	Bronchiectasis with Acute Exacerbation

15.6. Education and process outcomes

Hypothesis	What	Who	When	Why	Who Collects?
2a	Time-motion	PGY1	Jan-Feb 2016	Type of activities engaged in	CCC
2b	Just-in-Time surveys	PGY1 in target IM rotations	Random daily samples	Work intensity, ownership, continuity	CCC
2b	Satisfaction	PGY1-3	May 2015 (baseline)<br>May 2016	Attitudes	CCC
2b	Maslach Burnout Inventory	PGY1-3	May 2015 (baseline)<br>May 2016	Burnout	CCC
2b	ACGME year-end trainee survey	PGY1-3	May 2015 (baseline)<br>May 2016	Attitudes, perceptions of training	ACGME
2c	ACGME core faculty survey	Core faculty	May 2015 (baseline)<br>May 2016	Perceptions of safety, teamwork, supervision	ACGME
2c	PD satisfaction	PD	May 2015 (baseline)<br>May 2016	Clinical teaching satisfaction, costs	CCC
2c	PD Perceptions	PD	Fall 2015<br>Fall 2016	Morale, continuity, education, schedules	APDIM
2d	In-Training Examination	PGY1	Early PGY2 year 2015 (baseline)<br>Early PGY2 year 2016	Knowledge	ACP

15.7. Data collection schedule

Data item	Group doing the primary data collection	Who provides data	When (raw) data are/were collected	When data are received by iCOMPARE
Program characteristics survey	iCOMPARE	PDs	Summer-Fall 2015	Summer-Fall 2015
Trainee satisfaction and burnout year-end survey	iCOMPARE	PGY1-PGY3	May 2015 (baseline)<br>May 2016 (post intervention)	May 2015<br>May 2016
ACGME trainee year-end survey	ACGME	PGY1-PGY3	May 2015 (baseline)<br>May 2016 (post intervention)	Winter 2016<br>Winter 2017
Program director satisfaction year-end survey	iCOMPARE	PDs	June 2015 (baseline)<br>June 2016 (post intervention)	June 2015<br>June 2016
APDIM program director survey	APDIM	PDs	Fall 2015 (baseline)<br>Fall 2016 (post intervention)	Winter 2016<br>Winter 2017
ACGME core faculty year-end survey	ACGME	Faculty	May 2015 (baseline)<br>May 2016 (post intervention)	Winter 2016<br>Winter 2017
In-Training Examination (ITE) score	ACP	PGY2	Fall 2015 (baseline)<br>Fall 2016 (post intervention)	Winter 2016<br>Winter 2017
Just-in-time (JIT) surveys of activities, perceptions	iCOMPARE	PGY1-PGY3 at programs with IRB approval for JIT, group receiving surveys expands as IRB approvals are obtained	q14 days,<br>Aug 2015-June 2016	Aug 2015-June 2016
Sleep and Alertness Substudy data (actigraphy, PVT, other alertness questions)	iCOMPARE	Consenting PGY1 at S+A sites	Nov 2015 thru May 2016, 14 days of data per intern	Nov 2015 thru May 2016

Data item	Group doing the primary data collection	Who provides data	When (raw) data are/were collected	When data are received by iCOMPARE
Time and Motion Substudy data (observation data recorded by trained observer)	iCOMPARE	Consenting PGY1 at T+M sites	Mar 2016 thru May 2016	Mar 2016 thru May 2016
Patient data	Medicare	CMS (ResDAC)		
Pre baseline period 1, Jul 2013-Jun 2014:				
CY 2013 data			CY 2013	To be requested from CMS in Fall 2015
CY 2014 data			CY 2014	To be requested from CMS in Fall 2015
Pre baseline period 2, Jul 2014-Jun 2015:				
CY 2014 data			CY 2014	To be requested from CMS in Fall 2015
CY 2015 data			CY 2015	To be requested from CMS in Fall 2016
Intervention year, Jul 2015-Jun 2016:				
CY 2015 data			CY 2015	To be requested from CMS in Fall 2016
CY 2016 data			CY 2016	To be requested from CMS in Fall 2017

15.8. Data collection by when raw data accrue and when raw data are collected, requested, received by iCOMPARE

Data collection by time when raw data accrue

	CY 2013				CY 2014				CY 2015				CY 2016				CY2017				CY2018				CY2019			
	Q1	Q2	Q3	Q4	Q1	Q2	Q3	Q4	Q1	Q2	Q3	Q4	Q1	Q2	Q3	Q4	Q1	Q2	Q3	Q4	Q1	Q2	Q3	Q4	Q1	Q2	Q3	Q4
iCOMPARE program characteristics survey										B																		
iCOMPARE trainee satisfaction and burnout year-end survey										B				P														
ACGME trainee year end survey										B				P														
iCOMPARE PD satisfaction year-end survey										B				P														
APDIM program director survey											B				P													
ACGME core faculty survey										B				P														
ITE examination											B				P													
JIT surveys											■	■	■	■														
Sleep and Alertness Substudy data												■	■	■														
Time and Motion Substudy data													■	■														
Medicare claims data			B1	B1	B1	B1	B2	B2	B2	B2	■	■	■	■														

Legend:
■ = Data accrual ongoing during intervention year
B = Baseline (pre intervention) data collection
B1, B2 = Two baseline periods are constructed for the claims data analyses
P = Post intervention data collection

Data collection by time when raw data are collected/requested/received by iCOMPARE

	CY 2013				CY 2014				CY 2015				CY 2016				CY2017				CY2018				CY2019			
	Q1	Q2	Q3	Q4	Q1	Q2	Q3	Q4	Q1	Q2	Q3	Q4	Q1	Q2	Q3	Q4	Q1	Q2	Q3	Q4	Q1	Q2	Q3	Q4	Q1	Q2	Q3	Q4
iCOMPARE program characteristics survey										R																		
iCOMPARE trainee satisfaction and burnout year-end survey										R				R														
ACGME trainee year end survey													R			R												
iCOMPARE PD satisfaction year-end survey										R				R														
APDIM program director survey													R				R											
ACGME core faculty survey													R			R												
ITE examination													R				R											
JIT surveys											R	R	R	R														
Sleep and Alertness Substudy data												R	R	R														
Time and Motion Substudy data													R	R														
Medicare claims data												R	R			R	R			R	R			R	R			

Legend: R = iCOMPARE receives data item

16. References

1. Friedman, R.C., J.T. Bigger, and D.S. Kornfeld, *The intern and sleep loss.* N Engl J Med, 1971. **285**(4): p. 201-3.
2. Asch, D.A. and R.M. Parker, *The Libby Zion case. One step forward or two steps backward?* N Engl J Med, 1988. **318**(12): p. 771-5.
3. Philibert, I., P. Friedmann, and W.T. Williams, *New requirements for resident duty hours.* JAMA, 2002. **288**(9): p. 1112-4.
4. Prasad, M., et al., *Effect of work-hours regulations on intensive care unit mortality in United States teaching hospitals.* Crit Care Med, 2009. **37**(9): p. 2564-9.
5. Press, M.J., et al., *The impact of resident duty hour reform on hospital readmission rates among Medicare beneficiaries.* J Gen Intern Med, 2011. **26**(4): p. 405-11.
6. Rosen, A.K., et al., *Effects of resident duty hour reform on surgical and procedural patient safety indicators among hospitalized Veterans Health Administration and Medicare patients.* Med Care, 2009. **47**(7): p. 723-31.
7. Silber, J.H., et al., *Prolonged hospital stay and the resident duty hour rules of 2003.* Med Care, 2009. **47**(12): p. 1191-200.
8. Volpp, K.G., et al., *Mortality among patients in VA hospitals in the first 2 years following ACGME resident duty hour reform.* JAMA, 2007. **298**(9): p. 984-92.
9. Volpp, K.G., et al., *Mortality among hospitalized Medicare beneficiaries in the first 2 years following ACGME resident duty hour reform.* JAMA, 2007. **298**(9): p. 975-83.
10. Volpp, K.G., et al., *Did duty hour reform lead to better outcomes among the highest risk patients?* J Gen Intern Med, 2009. **24**(10): p. 1149-55.
11. Patel, M.S., et al., *Association of the 2011 ACGME resident duty hour reforms with morality and readmissions among hospitalized Medicare patients.* JAMA, 2014. **312**: p. 2364-2373.
12. Ulmer, C., D. Miller Wolman, and M.M.E. Johns, in *Resident Duty Hours: Enhancing Sleep, Supervision, and Safety*. 2009, 2009 by the National Academy of Sciences: Washington DC.
13. Nasca, T.J., S.H. Day, and E.S. Amis, Jr., *The new recommendations on duty hours from the ACGME Task Force.* N Engl J Med, 2010. **363**(2): p. e3.
14. Block, L., et al., *In the wake of the 2003 and 2011 duty hours regulations, how do internal medicine interns spend their time?* J Gen Intern Med, 2013. **28**(8): p. 1042-7.
15. Desai, S.V., et al., *Effect of the 2011 vs 2003 duty hour regulation-compliant models on sleep duration, trainee education, and continuity of patient care among internal medicine house staff: a randomized trial.* JAMA Intern Med, 2013. **173**(8): p. 649-55.
16. Sen, S., et al., *Effects of the 2011 duty hour reforms on interns and their patients: a prospective longitudinal cohort study.* JAMA Intern Med, 2013. **173**(8): p. 657-62; discussion 663.
17. Drolet, B.C., D.A. Christopher, and S.A. Fischer, *Residents' response to duty-hour regulations--a follow-up national survey.* N Engl J Med, 2012. **366**(24): p. e35.
18. Drolet, B.C., M.T. Khokhar, and S.A. Fischer, *The 2011 duty-hour requirements--a survey of residency program directors.* N Engl J Med, 2013. **368**(8): p. 694-7.
19. Drolet, B.C., L.B. Spalluto, and S.A. Fischer, *Residents' perspectives on ACGME regulation of supervision and duty hours--a national survey.* N Engl J Med, 2010. **363**(23): p. e34.
20. Shea, J.A., et al., *A randomized trial of a three-hour protected nap period in a medicine training program: sleep, alertness, and patient outcomes.* Acad Med, 2014. **89**(3): p. 452-9.
21. Volpp, K.G., et al., *Effect of a protected sleep period on hours slept during extended overnight in-hospital duty hours among medical interns: a randomized trial.* JAMA, 2012. **308**(21): p. 2208-17.
22. Silber, J.H., et al., *Conditional length of stay.* Health Serv Res, 1999. **34**(1 Pt 2): p. 349-63.

23. Silber JH, R.P., Even-Shoshan O, Shabout M, Zhang X, Bradlow ET, Marsh RR, *Length of stay, conditional length of stay and prolonged stay in pediatric asthma.* Health Serv Res, 2003. **38**: p. 867-86.
24. Kaida, K., et al., *Validation of the Karolinska sleepiness scale against performance and EEG variables.* Clinical Neurophysiology, 2006. **117**: p. 1574-1581.
25. Basner, M., D. Mollicone, and D.F. Dinges, *Validity and sensitivity of a brief psychomotor vigilance test (PVT-B) to total and partial sleep deprivation.* Acta Astronautica, 2011. **69**: p. 949-959.
26. *HCUP Nationwide Inpatient Sample (NIS)*, H.C.a.U.P. (HCUP), Editor. 2011.
27. Du, X.L., C.R. Key, and L. Dickie, *External validation of Medicare claims for breast cancer chemotherapy compared iwth medical chart reviews.* Med Care, 2006. **44**: p. 124-131.
28. Virnig, B. and A. Madeira, *Strengths and limitations of CMS administrative data in research*. 2012, Research Data Assistance Center
29. Volpp, K.G., et al., *Teaching hospital five-year mortality trends in the wake of duty hour reforms.* J Gen Intern Med, 2013. **28**(8): p. 1048-55.
30. Rothmann, M.D., B.L. Wiens, and I.S.F. Chan, *Design and Analysis of Non-Inferiority Trials.* Design and Analysis of Non-Inferiority Trials, 2012: p. 1-433.
31. Shea, J.A., et al., *Internal medicine trainees' views of training adequacy and duty hours restrictions in 2009.* Acad Med, 2012. **87**(7): p. 889-94.
32. Shea, J.A., et al., *Anticipated consequences of the 2011 duty hours standards: views of internal medicine and surgery program directors.* Acad Med, 2012. **87**(7): p. 895-903.
33. Borman, K.R., et al., *Sleep, supervision, education, and service: views of junior and senior residents.* J Surg Educ, 2011. **68**(6): p. 495-501.
34. Borman, K.R., A.T. Jones, and J.A. Shea, *Duty hours, quality of care, and patient safety: general surgery resident perceptions.* J Am Coll Surg, 2012. **215**(1): p. 70-7; discussion 77-9.

3. Summary of iCOMPARE protocol changes

The iCOMPARE protocol first received IRB review and approval in Oct 2014. At that time, the protocol was documented in the submission to the University of Pennsylvania IRB and encompassed these activities:

Enrollment and randomization of internal medicine training programs
Analysis of data collected by the ACGME, ACP, and APDIM and provided to iCOMPARE investigators
Surveys of trainees and program directors administered by iCOMPARE

This October 2014 protocol included randomizing programs to flexible or standard duty hour standards and hypotheses regarding patient safety and education. It did not include hypotheses about sleep and alertness outcomes.

Although proceeding with the October 2014 protocol, the iCOMPARE investigators decided to submit a revised grant application to NHLBI in November of 2014. This grant application was ultimately funded in summer 2015. It added the Time-Motion Substudy and Sleep and Alertness Substudy to the October 2014 protocol and expanded the number of surveys of trainees to be administered by iCOMPARE. NHLBI required that the trial include a Data and Safety Monitoring Board (DSMB). A stand alone protocol document was written; the DSMB first reviewed this expanded protocol in October 2015; after minor revisions, the DSMB approved the Protocol in December 2015. No amendments have been implemented since that initial DSMB approval.

iCOMPARE

individualized Comparative Effectiveness Models Optimizing Patient Safety and Resident Education

Statistical Analysis Plan

22 December 2015

Table of Contents

1 Introduction ... 3
2 Data sources ... 4
3 Sample size and power ... 4
4 Data analysis ... 5
4.1 Patient safety hypothesis H1a – 30-day mortality ... 5
4.1.1 Risk adjustment using aggregate predicted mortality ... 6
4.1.2 Risk adjustment using aggregate predicted mortality ... 7
4.1.3 Hierarchical models ... 7
4.1.4 Potential effect modification ... 8
4.2 Other patient safety hypotheses – H1b-H1e ... 8
4.3 Trainee education hypotheses – H2a-H2d ... 9
4.4 Intern sleep and alertness hypotheses – H3a-H3b ... 10
5 Missing data ... 10
6 Intern safety outcomes ... 10
7 Interim monitoring ... 10

1 Introduction

iCOMPARE is a cluster randomized trial comparing the current duty hour standards for interns (**Curr**) with a more flexible duty hour schedule (**Flex**) in at least 58 Internal Medicine training programs. Training programs randomized to **Flex** must schedule intern duty hours in conformance with 3 rules, each averaged over 4 weeks:

1. Work no more than 80 hours per week
2. Call no more frequent than every 3rd night
3. 1 day off in 7

Current duty hour standards (**Curr**) require that interns work no more than 16h of duty periods in a day.

iCOMPARE has one primary hypothesis:

H1a: 30-day patient mortality under **Flex** will not exceed (will not be inferior to) mortality under **Curr**.

iCOMPARE will test related and complementary secondary hypotheses regarding:

Patient safety and costs:

H1b: 7-day and 30-day hospital readmission rates under **Flex** will not exceed (will not be inferior to) the rates under **Curr**.

H1c: Complication rates, defined by selected AHRQ Patient Safety Indicators, under **Flex** will not exceed (will not be inferior to) complication rates under **Curr**.

H1d: The rate of prolonged length of stay, defined as a stay that exceeds the Hollander-Proschan point or that length of stay for a given condition at which the discharge rate begins to decline (1, 2), under **Flex** will not exceed (will not be inferior to) the rate of prolonged length of stay under **Curr**.

H1e: Overall costs, as indicated by total Medicare payments, under **Flex** will not exceed (will not be inferior to) overall costs under **Curr**.

Trainee education:

H2a: Interns in **Flex** will spend greater time in direct patient care and education compared to interns in **Curr**.

H2b: Trainees in **Flex** will report greater satisfaction with their educational experience (greater ownership, greater continuity and lower burnout) than trainees in **Curr**.

H2c: Faculty in **Flex** will report greater satisfaction with their clinical teaching experiences and greater perceptions of safety, teamwork and supervision than faculty in **Curr**.

H2d: Standardized test scores for interns in **Flex** will not be less than (inferior to) those for interns in **Curr**.

and Intern sleep and alertness:

H3a: Average daily sleep obtained by interns in **Flex** will not be less than (will not be inferior to) that of interns in **Curr**, as determined by a 14-day period of sleep monitoring using actigraphy and daily sleep diaries.

H3b: Interns in **Flex** will not have (will not be inferior to) greater average subjective sleepiness via Karolinska Sleepiness Score (KSS) (3), or lower average behavioral alertness via psychomotor vigilance test (PVT) (4, 5) than interns in **Curr**, as determined by a 14-day period of morning sleepiness-alertness monitoring.

The iCOMPARE primary outcome (30-day mortality) was chosen to ensure that any policy change in resident duty hours will not result in inferior patient safety. However, additional patient safety measures, as well as costs, education and fatigue management, are critically important considerations which our study addresses. The results of iCOMPARE will help the ACGME in its ongoing deliberations about optimal resident duty hour schedules. Changes in ACGME policies affect every teaching hospital in the United States, and as a consequence, every patient.

2 Data sources

Data on patient outcomes and costs of health care will come from Medicare claims records. These records will be obtained through application to and purchase from the Research Data Assistance Center located at the University of Minnesota School of Public Health (ResDAC; http://www.resdac.org/). All requests for Medicare data proceed through ResDAC. We will obtain claims data from Medicare for calendar years 2013 through 2016.

Data collected directly from trainees and program directors through iCOMPARE surveys, actigraphy, and/or observation will be supplemented with data collected on trainees, program directors and faculty by national organizations such as the ACGME, the American College of Physicians (ACP) and the Association of Program Directors in Internal Medicine (APDIM). The time period of direct data collection by iCOMPARE survey, actigraphy, or observation will be May 2015 through June 2016. The data obtained from the ACGME, APDIM, and ACP includes responses to their 2015 and 2016 surveys (ACGME, APDIM) and ITE scores (ACP) from fall 2015 and fall 2016.

The Medicare claims data will be received, analyzed and stored at Children's Hospital of Philadelphia. Surveys are administered by the CCC located at University of Pennsylvania and responses are collected by the CCC; files will be transferred to the DCC periodically for review and analysis. Sleep and Alertness Substudy data will be collected by Pulsar, transferred to the Sleep and Alertness Substudy group for post collection processing, and then transferred periodically to the DCC for review and analysis. The data received by the DCC will be devoid of personal identifiers such as name, phone number, and email address. Time and Motion Substudy data will be collected by the Time and Motion Substudy group and transferred periodically to the DCC for review and analysis. The data received by the DCC will be devoid of personal identifiers such as name, phone number, and email address. Backup files of the database at the DCC will be generated and stored at regular intervals in a secure, off-site location, to permit regeneration of the database in the event that it is destroyed. Freeze dates for data sets created for interim and publication analyses will be documented.

3 Sample size and power

We approached the statistical design by designating the non-inferiority mortality hypothesis H1a (30-day patient mortality under **Flex** will not exceed (will not be inferior to) mortality under **Curr)** as the primary hypothesis for which sample size calculations were based. The 30-day mortality outcome measure is defined, for each IM program, as the difference between the 30-day mortality rate in the trial year minus the 30-day mortality rate in the pre-trial year. Section 4.1 includes details on the definition and analysis plan for the primary outcome.

The 30-day mortality rate in the iCOMPARE target population was estimated to be 11% [11.1% in 2007 and 11.5% in 2008; personal communication from Dr. Silber] and the pooled standard deviation (SD) for

the pairs of rate differences was estimated to be 1.5%. The consensus non-inferiority mortality margin among the iCOMPARE investigators was 1%. We used the PASS 11 software (http://www.ncss.com/) to calculate the sample size required for hypothesis H1a. The PASS 11 software accommodates the complex statistical calculations needed to allow for superiority or non-inferiority hypotheses, and correlations in responses such as those we will see due to clustering on the internal medicine programs.

We performed sample size calculations with both 80% and 90% power to gauge any gains in power by recruiting beyond the N=58 IM programs required for 80% power. The results of the calculations for mortality non-inferiority are as follows, where Type-1 error (alpha) is based on a one-sided t-test as is appropriate for a non-inferiority design.

		Non-inferiority Margin	Actual Difference	Significance Level		Standard Deviation 1 (Curr)	Standard Deviation 2 (Flex)
Power	N1 (Curr) /N2 (Flex)	(NIM)	(D)	(Alpha)	Beta	(SD1)	(SD2)
0.8059	29/29	0.01	0	0.05	0.1941	0.015	0.015
0.9050	40/40	0.01	0	0.05	0.0950	0.015	0.015

Although sample size calculations were based on the mortality outcome, this number of programs will give excellent power for other study hypotheses. The 58 randomized programs are expected to include 4640 internal medicine residents: 1740 interns (approximately 30 interns per program) and approximately 1450 PGY2 trainees (approximately 25 PGY2 per program) and 1450 PGY3 trainees (approximately 25 PGY3 per program). Each program will include one program director (total of 58) and approximately 10 associated faculty (total of about 580 faculty).

The sample size for the Time and Motion Substudy of 6 programs was a practical choice; this Substudy addresses hypothesis H2a. Preliminary data (6, 7) suggest that the mean percent time spent in direct patient care and education in **Curr** will be about 13% (SD = 4%). With 60 interns (10 each from the 6 IM programs), we will be able to detect a 3% difference in the time spent outcome between **Curr** and **Flex** with greater than 80% power.

Hypothesis H3a was designated as the primary hypothesis for the Sleep and Alertness Substudy. For H3a, with 90% power, one-sided Type I error of 0.05, and a non-inferiority margin of 0.5 hours, the required sample size is 290 interns. The proposed sample size is higher: 384 interns (48 at each of 8 programs) for H3a.

4 Data analysis

4.1 Patient safety hypothesis H1a – 30-day mortality

The primary outcome will be the difference between the pre-trial year and trial year 30-day mortality rates as described above.

The model (model A1) is:

$Y_i = \gamma + \beta_1 x_i + \varepsilon_i, \; i = 1, \dots, n_p \; where$
$Y_i =$ Outcome measure in IM program i =*Trial year rate - Pre-trial year rate,*
γ = the intercept in the reference group (**Curr**),
β_1 = the difference in intercepts between **Flex** and **Curr**,
x_i = 1 if the *i*th IM program is in **Flex**, 0 if the *i*th IM program is in **Curr**
ε_i = i.i.d. random Gaussian errors with mean 0 and variance σ^2
n_p = Number of clusters (IM programs)

We will test β_1 (or $\beta_1 - nim$, where *nim* is the non-inferiority margin)≤ 0; β_1 is the expected difference in outcome: **Flex** vs. **Curr**. All randomized programs will be included in this model and we expect no missing data.

The primary outcome measure for this cluster-randomized trial is the difference in 30-day mortality rates during the trial year versus the prior, which is 58 rate differences, 1 per randomized program, with a two-sample t-test for non-inferior patient mortality (difference no greater than 1%), comparing the **Flex** vs. **Curr** schedules. This approach permits the use of a simple model (two-sample non-inferiority t-test) for the set of at least N=29 pairs of trial year vs. pre-trial year differences in each group (**Curr** vs. **Flex**) in annual 30-day mortality rates following the paradigm elaborated by Hayes and Moulton (8) in their book on cluster randomized trials (CRTs). The model obviates the need for complex risk adjustment models, since it adjusts for secular trends in 30-day mortality that were common to all hospitals (e.g., severe flu season, technological improvements), as well as removing variation due to IM program population risk profiles (e.g., geography, patient characteristics) that are likely to cancel out by comparing successive years within IM program.

While these rate differences should adjust for most program to program differences in patient and institutional mix, we will also perform an important series of sensitivity analyses using risk-adjustment modeling methods that we developed (9) and which we will apply to the iCOMPARE data to confirm the findings of the primary analysis or to add a risk-adjustment caution to the statements of findings.

4.1.1 Risk adjustment using aggregate predicted mortality

Briefly, sensitivity analyses will use iCOMPARE program-level risk-adjusted data on 30-day mortality by aggregating the patient-level risks derived from our fitted models. We will compute each patient's predicted 30-day mortality in the pre-trial period as we did in previous work (9-11). We will then average the predicted 30-day mortality for all patients in a program in the pre-trial period to obtain program *i*'s expected 30-day mortality rate in the pre-trial period, call it RA_{pi}, and average the predicted 30-day mortality for all patients in a program in the trial period to obtain what program *i*'s expected 30-day mortality rate would have been had these same patients been treated in the pre-trial period, which we call RA_{ti}. Then we update model A1 (at the program level) to include a term for the difference (RA_{ti} - RA_{pi}) so that the coefficient on treatment assignment is our estimate of being assigned to treatment versus not being assigned on the change in the outcome (e.g., mortality rate), adjusting for any changes in the risks of the patients at the program.

The model (Model B) is:

$Y_i = \beta_0 + \beta_1 x_{1i} + \beta_2 x_{2i} + \varepsilon_i, \; i = 1, \dots, n_p, \; where$
Y_i
$=$ Mean outcome measure in IM program i (e.g., Trial year 30-day mortality - Pre-trial 30-day mortality)

x_{1i} = 1 if the ith IM program is in **Flex**, 0 if the ith IM program is in **Curr**
$x_{2i} = (RA_{ti} - RA_{pi})$
β_0 = Intercept for the IM programs
ε_i = i.i.d. random Gaussian errors with mean 0 and variance σ^2
n_p = Number of clusters (IM programs)

The adjusted non-inferiority tests will be t-tests based using the same non-inferiority margin (1%) and the estimates and standard errors for β_1 from the regression models. The model estimates, 95% CIs, and p-values will be derived using Stata, R or SAS.

4.1.2 Risk adjustment using aggregate predicted mortality

A key set of secondary analysis will use multivariable risk adjustment modeling used by our DCC group in past analyses of Medicare data for non-randomized comparisons of resident duty hour policy changes (9, 10). These secondary analyses related to our primary aim of whether 30-day mortality under the Flex duty hour schedule is not inferior to 30-day mortality under the **Curr** duty hour schedule and will use multivariable program-level and patient-level risk adjusted analyses appropriate for multiple time series research designs like iCOMPARE. The model will be a multivariate risk adjusted logistic models for patient level 30-day mortality in relation to **Flex** vs. **Curr**, year, **Flex** vs. **Curr** by year interaction, and program-level and patient-level risk factors for mortality. These designs, also known as "Difference-in-Differences" designs, reduce potential biases from unmeasured variables. The multiple time series research design compares each program with itself (comparison of an outcome in the year before the duty hour intervention versus the outcome in the year of the duty hour intervention), making adjustments for observed differences in patient risk factors and adjustments for changes in outcomes over time (trends) that were common to all programs. This design prevents bias from 3 possible sources. First, a difference between programs that is stable over time cannot be mistaken for an effect of the trial, because each program is compared with itself. Because of this, program indicators for fixed effects are used in the logistic model. Second, changes over time that affect all programs similarly (e.g., technological improvements) cannot be mistaken for an effect of the trial. Because of this, year indicators are used in the logistic model. Third, if the mix of patients is changing in different ways at different programs, and if these changes are accurately reflected in measured risk factors, this cannot be mistaken for an effect of the duty hour schedule because the logistic model adjusts for these measured risk factors. For example, this approach addresses concern about local differences in, for example, influenza rates that may affect the distribution of patient diagnoses or outcomes year-to-year inside the same hospital. However, we will be including the principal diagnosis and secondary diagnoses in the Difference-in-Differences model, so the distribution of diagnoses will be accounted for in the adjustment model, eliminating concerns about this type of potential bias.

4.1.3 Hierarchical models

We will do another sensitivity analysis using hierarchical logistic regression models for patient-level 30-day mortality in relation to fixed training program-level and patient-level risk factors **Flex** vs. **Curr**, year (trial vs pre-trial), **Flex** vs. **Curr** by year interaction and random effects to account for within-program correlations consisting of programs, hospitals, and patients. Hierarchical models are a different approach to risk adjustment and adjustment for within cluster correlation. We summarized our approach above. The key issues with such models relate to assumptions about the random effects (we assume Gaussian deviations in effects) and which effects are fixed, which are random, and which are both. These are difficult choices to make at the design phase.

However, hierarchical models do have appeal. For example, it is possible that there are things that affect a whole program or hospital in a specific year, e.g., a new program director is hired one year. The fixed

effects take account of program effects that are constant across all years of study, but not effects that are just present for one particular year. While we believe that single year effects will be rare and limited to a few hospitals, one approach would be to use robust standard errors that are clustered on training programs. This is the approach suggested by Angrist and Pischke (12) when there are at least 42 clusters, which is our setting. However, fitting a random effects model with random effects for program, hospital, attending, and patient would also be a way to account for these program*year effects, but probably harder to fit. Another issue relates to the few cases where one hospital has multiple programs in it so that there would be crossed random effects between program and hospital. In rare cases, a patient can be seen by two different programs at different times, this would also create crossed random effects. In principle, these configurations make the approach suggested above using robust standard errors that are clustered on program not fully valid as it would not take into account this crossing. Crossed random effect models are considerably harder to fit than nested random effect models, but it might be possible.

Despite, potential difficulties, we believe that hierarchical models will give us exploratory insights into our data and may help to understand effects or lack of effects arising from iCOMPARE.

4.1.4 Potential effect modification

Despite the sub-optimal power for comparisons of variability in non-inferiority of **Flex** versus **Curr** across subgroups of programs, we will explore the extent to which the effect of the **Flex** versus **Curr** schedule on the outcomes listed above differs in several subgroups of interest. We will compare the treatment effects in subgroups by testing for treatment (**Flex** vs. **Curr**) by subgroup interactions in the models described above. The pre-specified key subgroups of interest are 1) nonteaching versus teaching hospitals (2, 9), 2) resident to bed ratio (10) (categorized into tertiles), 3) percentage of inpatients (2) (categorized into tertiles), 4) geography (northeast, southeast, midwest, southwest, west). Other subgroups of interest may be examined in response to journal reviews or other, as yet unstated, reasons.

4.2 Other patient safety hypotheses – H1b-H1e

The following outcomes are the remaining outcomes for the patient safety and cost hypotheses:

a) Patient safety and costs hypothesis H1b:
- Measure: 7-day and 30-day hospital readmission rates
- Non-inferiority margin: 1%

b) Patient safety and costs hypothesis H1c:
- Measure: complications rates, defined by selected AHRQ Patient Safety Indicators
- Non-inferiority margin: 1%

c) Patient safety and costs hypothesis H1d:
- Measure: The rate of prolonged length of hospital stay defined as a stay that exceeds the Hollander-Proschan point or that length of stay for a given condition at which the discharge rate begins to decline (1)
- Non-inferiority margin: 1%

d) Patient safety and costs hypothesis H1e:
- Measure: Overall resources utilized and Medicare payments for patient care
- Non-inferiority margin: 1%

Analyses for H1b-e will use the same approach described for model A1 with either linear, logistic, or Poisson models depending on whether the outcome measure is a rate, measured/ordered, a proportion, or a count.

4.3 Trainee education hypotheses – H2a-H2d

The following outcomes are the outcomes for the trainee education hypotheses:

a) Education hypothesis H2a:
- Measure: Direct patient care and education measured from Time and Motion Substudy, specifically percent of time spent by the intern in direct patient care
- Minimum important difference is 3% (0.75 SD)

b) Education hypothesis H2b:
- Measure: Trainee satisfaction with their educational experience measured from surveys, primarily the trainee's perception of having an 'appropriate balance for education', measured on an ordinal scale with expected mean of 3.7 (SD 0.7) in the **Curr** schedule (13-16)
- Minimum important difference is 0.175

c) Education hypothesis H2c:
- Measure: Faculty satisfaction with their clinical teaching experiences measured from surveys, primarily the faculty ranking on 'residents workload exceeds capacity to do the work' from the ACGME survey, measured on an ordinal scale with expected mean of 4.1 (SD 0.7) in the **Curr** schedule (13-16)
- Minimum important difference is 0.175

d) Education hypothesis H2d:
- Measure: Standardized test scores for interns on the In-Training Examination (ITE), measured as the percent correct with expected mean of approximately 65 (SD 18) in the **Curr** schedule [Lisa Bellini, personal communication].
- Non-inferiority margin is 2%

The trainee education analyses will be modeled using the model (Model A2):

$Y_{ij} = \gamma_i + \beta_1 x_{ij} + \varepsilon_{ij},\ i = 1, \dots, n_p;\ j = 1, \dots, n_i, \quad where$

$Y_{ij} =$ Mean outcome measure in IM for intern (or faculty or director) j in program i ,

$x_{ij} = 1$ if the ith IM program is in **Flex**, 0 if the ith IM program is in **Curr**

$\gamma_i =$ i.i.d. random Gaussian intercept for the ith IM program with mean β_0 and variance σ_1

$\beta_1 =$ difference in intercepts in **Flex** and **Curr**

$\varepsilon_{ij} =$ i.i.d. random Gaussian errors with mean 0 and variance σ^2

$n_p =$ Number of clusters (IM programs)

$n_i =$ Number of interns in ith IM program

Note that γ_i is the random intercept needed to account for clustering. Model A2 is a multilevel mixed effects model that may be estimated using the Stata software mixed command with REML estimates, R (lme4 package) or SAS (PROC MIXED). The hypotheses will be tested using model A2 with either

linear, logistic, or Poisson mixed effects models depending on whether the outcome measure is measured/ordered, a proportion, or a count.

4.4 Intern sleep and alertness hypotheses – H3a-H3b

The following outcomes are the outcomes for the intern sleep and alertness hypotheses:

a) Sleep hypothesis H3a:
- Measure: Average daily sleep measured by a 14-day period of sleep monitoring using actigraphy (verified by daily sleep diaries) with expected average sleep in **Curr** of 6.946 hours (SD=1.451 hours) [David Dinges, personal communication].
- Non-inferiority margin is 0.5 hours.

b) Sleep hypothesis H3b:
- Measures: Average subjective sleepiness measured by Karolinska Sleepiness Scale (KSS) (3) and behavioral alertness measured by the psychomotor vigilance test (4, 5)
- Non-inferiority margin: 1 unit on KSS Likert scale

The intern sleep and alertness hypotheses will be tested using Model A2 described above.

5 Missing data

The data for the primary outcome will be requested from ResDAC for each randomized program. We expect no missing data for any of the patient safety outcomes.

For the survey data, time and motion data, and sleep and alertness data, we will employ recommended strategies to prevent missing data, based on published research (17-20) and previous experience of the Data Coordinating Center.

If data are missing for reasons related to the study, i.e., informative censoring, the treatment effect estimate may be biased and we will perform sensitivity analyses using methods that have been described (21)such as multiple imputation techniques for missing data, best and worst-case scenarios, and use of the drop out event as a study end-point.

6 Intern safety outcomes

We will compare the rates of serious adverse events (SAEs) by duty hour group using negative binomial models. The following events will be considered SAEs: death or hospitalization of an intern, removal of an intern from their schedule or rotation because of mental of physical condition potentially related to their duty hours, motor vehicle accident in which the intern was the driver, needle stick experienced by intern, and other on the job injury to intern.

7 Interim monitoring

iCOMPARE data and safety will be monitored by the Steering Committee and by an independent Data and Safety Monitoring Board (DSMB) as required by NIH guidelines for multicenter trials. The Steering

Committee and the DSMB will monitor accumulating safety and performance data. No interim analyses are planned because the intervention phase will be completed before patient mortality data are available. Many of the education outcomes also will not be available until after completion of the intervention phase. However, during the intervention phase, the DSMB may monitor performance data, sleep and alertness outcomes, time and motion outcomes (as available), and reports of safety concerns relating to trainees, faculty or patients as provided by program directors or otherwise brought to the attention of the iCOMPARE investigators. The DSMB is a multidisciplinary group with a written charge, with members appointed by NHLBI. The DSMB will be advisory to the NHLBI. Details on the DSMB responsibilities, meetings and reports can be found in the DSMB charter.

References

1. Silber JH, Rosenbaum PR, Koziol LF, Sutaria N, Marsh RR, Even-Shoshan O. Conditional length of stay. Health Services Research. 1999;34:349-63.

2. Rajaram R, Chung JW, Jones AT, Cohen ME, Dahlke AR, Ko CY, et al. Association of the 2011 ACGME resident duty hour reform with general surgery patient outcomes and with resident examination performance. Jama. 2014;312:2374-84.

3. Akerstedt T, Gillberg M. Subjective and objective sleepiness in the active individual. Int J Neurosci. 1990;52:29-37.

4. Basner M, Dinges DF. Maximizing sensitivity of the psychomotor vigilance test (PVT) to sleep loss. Sleep. 2011;34:581-91.

5. Dorrian J, Rogers NL, Dinges DF, Kushida CA. Psychomotor vigilance performance: neurocognitive assay sensitive to sleep loss. Sleep Deprivation: Clinical Issues, Pharmacology and Sleep Loss Effects. New York, N.Y.: Marcel Dekker, Inc.; 2005. p. 39-70.

6. Fletcher KE, Visotcky AM, Slagle JM, Tarima S, Weinger MB, Schapira MM. The composition of intern work while on call. J Gen Intern Med. 2012;27:1432-7.

7. Block L, Habicht R, Wu AW, Desai SV, Wang K, Silva KN, et al. In the wake of the 2003 and 2011 duty hours regulations, how do internal medicine interns spend their time? J Gen Intern Med. 2013;28:1042-7.

8. Hayes RJ, Moulton LH. Cluster Randomized Trials: Chapman and Hall; 2009.

9. Patel MS, Volpp KG, Small DS, Hill AS, Even-Shoshan O, Rosenbaum L, et al. Association of the 2011 ACGME resident duty hour reforms with morality and readmissions among hospitalized Medicare patients. Jama. 2014;312:2364-73.

10. Volpp KG, Rosen AK, Rosenbaum PR, Romano PS, Even-Shoshan O, Wang Y, et al. Mortality among hospitalized Medicare beneficiaries in the first 2 years following ACGME resident duty hour reform. Jama. 2007;298(975-983).

11. Volpp KG, Rosen AK, Rosenbaum PR, Romano PS, Even-Shoshan O, Canamucio A, et al. Mortality among patients in VA hospitals in the first 2 years following ACGME resident duty hour reform. Jama. 2007;298:984-2.

12. Angrist JD, Pischke J-S. Mostly harmless econometrics: An empiricist's companion. 1st ed: Princeton University Press 2009.

13. Borman KR, Jones AT, Shea JA. Duty hours, quality of care, and patient safety: general surgery resident perceptions. J Am Coll Surg. 2012;215:70-7.

14. Borman KR, Biester TW, Jones AT, Shea JA. Sleep, supervision, education, and service: views of junior and senior residents. J Surg Educ. 2011;68:495-501.

15. Shea JA, Weissman A, McKinney S, Silber JH, Volpp KG. Internal medicine trainees' views of training adequcy and duty hours restrictions in 2009. Acad Med. 2012;87:889-94.

16. Shea JA, Willet LL, Borman KR, Itani KM, McDonald FS, Call SA, et al. Anticipated consequences of the 2011 duty hours standards: views of internal medicine and surgery program directors. Acad Med. 2012;87:895-903.

17. Booker CL, Harding S, Benzeval M. A systematic review of the effect of retention methods in population-based cohort studies. BMC Public Health. 2011;11:249.

18. The Prevention and Treatment of Missing Data in Clinical Trials. In: Panel on Handling Missing Data in Clinical Trials CoNS, Division of Behavioral and Social Sciences and Education, editor. Washington DC: The National Academies Press; 2010.

19. Mills E, Wilson K, Rachlis B, al e. Barriers to participation in HIV drug trials: a systematic review. Lancet Infectious Diseases. 2006;6:32-8.

20. Ross S, Grant A, Counsell C, al e. Barriers to participation in randomized controlled tirals: a systematic review. Journal of Clinical Epidemiology. 1999;52:1143-56.

21. Shih W. Problems in dealing with missing data and informative censoring in clinical trials. Curr Control Trials Cardiovasc Med. 2002;3:4.

5. Summary of changes to the iCOMPARE Statistical Analysis Plan

The DSMB first reviewed a draft statistical analysis plan in October 2015; after minor revisions, the DSMB approved the statistical analysis plan in December 2015. No amendments have been implemented since that initial DSMB approval.
